# Update on Inflammatory Biomarkers and Treatments in Ischemic Stroke

**DOI:** 10.3390/ijms17121967

**Published:** 2016-11-25

**Authors:** Aldo Bonaventura, Luca Liberale, Alessandra Vecchié, Matteo Casula, Federico Carbone, Franco Dallegri, Fabrizio Montecucco

**Affiliations:** 1 First Clinic of Internal Medicine, Department of Internal Medicine, University of Genoa, 6 viale Benedetto XV, 16132 Genoa, Italy; aldobon85@gmail.com (A.B.); lucamonticello@hotmail.it (L.L.); alessandra.vecchie@gmail.com (A.V.); matte.tel@hotmail.it (M.C.); federico.carbone@edu.unige.it (F.C.); dalle@unige.it (F.D.); 2 IRCCS Azienda Ospedaliera Universitaria San Martino—IST Istituto Nazionale per la Ricerca sul Cancro, Genova, 10 Largo Benzi, 16132 Genoa, Italy; 3 Centre of Excellence for Biomedical Research (CEBR), University of Genoa, 9 viale Benedetto XV, 16132 Genoa, Italy

**Keywords:** ischemic stroke, inflammation, biomarkers, neutrophils, injury, auto-antibodies

## Abstract

After an acute ischemic stroke (AIS), inflammatory processes are able to concomitantly induce both beneficial and detrimental effects. In this narrative review, we updated evidence on the inflammatory pathways and mediators that are investigated as promising therapeutic targets. We searched for papers on PubMed and MEDLINE up to August 2016. The terms searched alone or in combination were: ischemic stroke, inflammation, oxidative stress, ischemia reperfusion, innate immunity, adaptive immunity, autoimmunity. Inflammation in AIS is characterized by a storm of cytokines, chemokines, and Damage-Associated Molecular Patterns (DAMPs) released by several cells contributing to exacerbate the tissue injury both in the acute and reparative phases. Interestingly, many biomarkers have been studied, but none of these reflected the complexity of systemic immune response. Reperfusion therapies showed a good efficacy in the recovery after an AIS. New therapies appear promising both in pre-clinical and clinical studies, but still need more detailed studies to be translated in the ordinary clinical practice. In spite of clinical progresses, no beneficial long-term interventions targeting inflammation are currently available. Our knowledge about cells, biomarkers, and inflammatory markers is growing and is hoped to better evaluate the impact of new treatments, such as monoclonal antibodies and cell-based therapies.

## 1. Introduction

**Figure 1:**
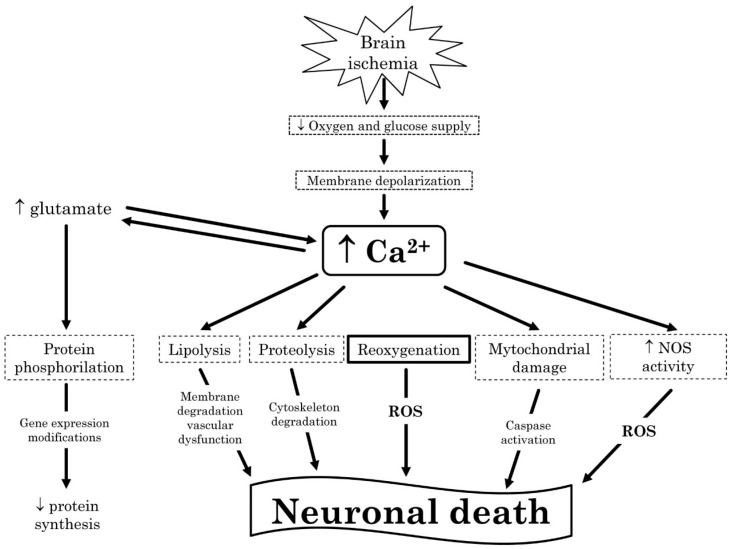
Mechanisms of neuronal death in acute ischemic stroke. After brain ischemia occurs, a reduction in the oxygen and glucose supply is carried out leading to the depolarization of membranes and finally to a great increase in intracellular calcium concentration and release of glutamate. Glutamate receptors, once activated, are responsible for the further increase in calcium concentration. The detrimental effects of this huge calcium concentration are proteolysis, lipolysis, mitochondrial damage, and increase in nitric oxide synthase activity. Despite the re-oxygenation should favor the recovery, it triggers reactive oxygen species production, amplifying the damage, ultimately leading to death of neuronal cells. Ca^2+^: calcium. NOS: nitrix oxide synthase. ROS: reactive oxygen species.

The World Health Organization defined “stroke” as a clinical syndrome characterized by a rapid onset of focal (or global in the case of coma) cerebral deficit lasting more than 24 h or leading to death, due to a vascular ischemic cause [1]. Ischemic stroke (IS) accounts for the majority of strokes and includes cryptogenic, lacunae, and thromboembolic strokes and it occurs usually when the blood supply to an area of the brain is interrupted [2]. Classical risk factors include age, cigarette smoking, diabetes, hypertension, and obesity. Prone-to-embolism diseases, such as cardiac valve disease and atrial fibrillation, increase the risk for IS, with the latter representing the most frequent condition. In IS, inflammation plays a pivotal role exerting both beneficial and detrimental effects (Figure 1). In fact, activation of resident cells, such as microglia, astrocytes, and endothelial cells is neuroprotective and promotes brain regeneration and recovery, whilst the recruitment of immune cells expressing inflammatory mediators and leading to blood-brain barrier (BBB) disruption is responsible for neuronal death, brain edema, and hemorrhagic transformation [3]. The sudden blockage of blood flow to the brain causes tissue hypoxia and triggers an inflammatory cascade leading to impairment of ion homeostasis, neuronal excitotoxicity, intracellular calcium overload, free radical generation, and lipid peroxidation ultimately determining neuronal injury [4]. The aim of the therapy is to mechanically or pharmacologically restore the blood flow as soon as possible to limit tissue damage. However, strangely, blood restoration is often associated with an exacerbation of tissue injury and a deep inflammatory response called “reperfusion injury” [5,6]. In this narrative review, based on papers found on PubMed and MEDLINE up to August 2016 (searched terms: ischemic stroke, inflammation, oxidative stress, ischemia reperfusion, innate immunity, adaptive immunity, autoimmunity; articles were also found through searches of reference lists and the authors’ files), we have updated the evidence regarding inflammatory cells, biomarkers, and pathways during and after an IS and speculate about new, potential therapeutic perspectives in order to reduce the detrimental effects carried by ischemia/reperfusion (I/R) in the brain following an IS.

## 2. Inflammatory Cells Involved in Post-Ischemic Brain Injury and Repair

The ischemic and reperfusion periods are both characterized by pathophysiological alterations, involving the modulation of inflammatory cell function [7]. In the following sub-paragraphs, we will update evidence on the major inflammatory cell subsets.

### 2.1. Neutrophils

Already few hours after the stroke onset, the number of circulating polymorphonuclear neutrophils (PMNs) rise in a stroke severity-dependent manner [8] as a result of different mechanisms: an increased release from the bone marrow and spleen reserves, an enhanced production, and a reduced apoptosis [9]. PMN rolling and adhesion have been described early after stroke onset in arterial vessels of the brain [10] due to the up-regulation of circulating and endothelial chemoattractants and adhesion molecules [11]. Early adhesion molecule-mediated PMN migration through brain vessels and parenchymal infiltration at ischemia site is now under discussion [11]. Recently, in experimental models and in human specimens it has been demonstrated that during the acute phase of reperfusion, PMNs are not found in the ischemic parenchyma, but remain in the neurovascular unit (NVU) and in the leptomeningeal spaces [12,13]. Here, they are attracted by accumulated signals released by ischemic brain in the interstitial fluid and then drained through perivascular spaces of cortical arterioles toward leptomeninges [12]. Accordingly, in vitro, ischemia alone was not able to induce PMN migration through BBB [13]. Only after prolonged ischemia (12 h), PMNs have been detected in cortical infarcted parenchyma [10].

**Figure 2:**
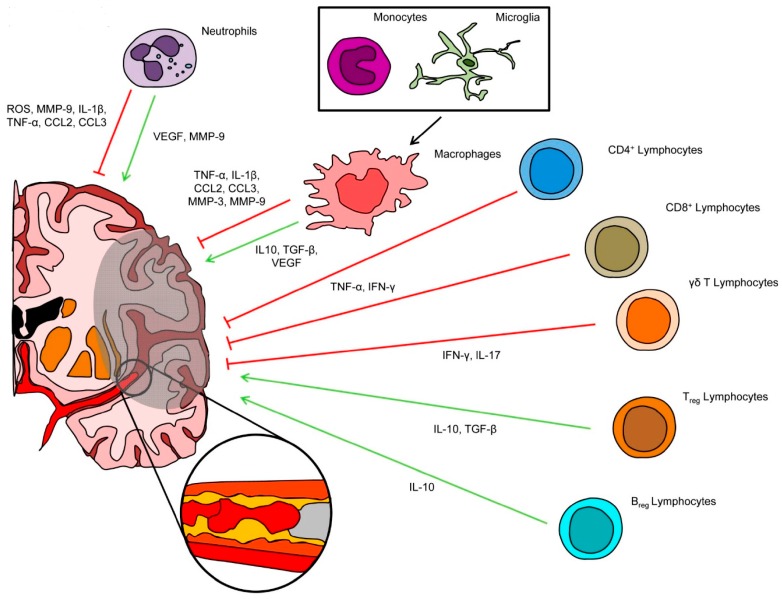
Inflammatory cells in post-ischemic brain injury and reparation. Inflammatory mediators released by the ischemic zone promote neutrophil activation and recruitment with a resulting effect on the blood-brain barrier and brain parenchyma. Resident macrophages of the brain (microglia) and circulating monocytes are early involved in the inflammatory response through an M1-switching with production of inflammatory mediators, such as reactive oxygen species, matrix metalloproteinases (MMPs), cytokines, and chemokines. Both neutrophils and macrophages (in particular M2 phenotype) are involved in late resolution through the production of anti-inflammatory and pro-angiogenic mediators. The adaptive immunity contribution is partly mediated by lymphocytes. CD4^+^, CD8^+^, and γδ T cells play a detrimental role, while T_reg_ and B_reg_ lymphocytes are involved in the resolution phase. IL: interleukin; TNF-α: tumour necrosis factor α; CCL: C-C motif chemokine ligand; VEGF: vascular endothelial growth factor; TGF-β: transforming growth factor β; IFN-γ: interferon γ.

Innate immune system answers to the cerebral damage through PMN activation attempting beneficial effects (e.g., contributing to resolution of inflammation, scar formation, and neo-angiogenesis) [14,15,16], but in this way contributing to BBB impairment and cerebral edema (Figure 2) [17]. PMN accumulation in the damaged tissue correlates with stroke severity and worst stroke outcome [18]. Activated PMNs enhance ischemic damage mainly through three mechanisms, such as reactive oxygen species (ROS) and protease production [19], cytokine-mediated enhanced inflammation, and complement activation [20]. On the basis of these findings, several studies tried to improve stroke outcomes by blocking PMN activation and recruitment, reducing their adhesion to endothelial cells or blocking mediators of BBB damage [21]. The complex relationship between PMNs, ischemic stroke, reperfusion injury, and the augmented infective risk following anti-neutrophil treatment explain the high rate of treatment failure and discrepancies hanging over these attempts.

### 2.2. Microglial Cells and Circulating Monocytes/Macrophages

Soon after IS onset, microglia (including Brain Res.ident macrophages) is activated and enhances circulating monocyte recruitment by releasing pro-inflammatory mediators, such as tumor necrosis factor (TNF)-α, nitric oxide (NO), and superoxide (Figure 2) [3]. Among different possible microglia-activating stimuli, released adenosine triphosphate from damaged cells seems to play an important role in animal models [22]. Microglia activation exerts both beneficial and detrimental effects on stroke outcomes. Once activated, in fact, resident macrophages can polarize toward different phenotypes in response to local ischemic *milieu* ranging from classically activated, pro-inflammatory macrophages type 1 (M1) to alternatively activated macrophages type 2 (M2), which are mainly involved in the resolution of inflammation and tissue healing (Figure 2) [23]. On the other hand, microglia inhibition in experimental models of brain injury leads to contradicting results in terms of neuronal survival [24,25,26,27].

In contrast to early microglial response, monocyte-derived macrophages from bloodstream reach the damaged site most abundantly 3–7 days after ischemia onset during the chronic phase of IS [28]. Early after brain injury, an increased number of total monocytes in the blood circulation has been described in humans [29]. About different subtypes, classical monocyte subset (CD14^++^ CD16^−^) significantly increased, while the non-classical monocyte subset (CD14^+^ CD16^++^) significantly decreased [29]. Progression and severity of brain ischemia directly correlate with CD14^++^ CD16^−^ increase and CD14^+^ CD16^++^ reduction [29]. As already known in another model [30], the splenic monocyte reserves were critical in the pathophysiology of brain infarction [31,32]. Although different studies reported that splenectomy or spleen irradiation reduced ischemic cerebral injury in animal models [33,34,35], some concerns rise from a work by Kim and colleagues [36]. In this study, spleen contraction after brain injury was accompanied by decreased number of Ly-6C^high^ and Ly-6C^low^ subsets in the spleen and temporally coincided with respective increases in the ischemic brain. Despite the reduced accumulation of both subsets in injured brain, splenectomy failed to reduce infarct size and swelling. Subset-specific studies and more homogeneity in stroke model (e.g., permanent vs. transient artery occlusion) are needed to fully address spleen function in brain infarction.

Peripheral monocyte heterogeneity corresponds to the presence of different macrophage subsets in the infarcted parenchyma, where these cells can shift from a phenotype to another depending on the local milieu [37]. Although exact contribution of different cellular subsets on lesion evolution is still elusive, the early monocyte recruitment at stroke hemisphere has been demonstrated to mainly involve pro-inflammatory Lys6C^high^ cells. Once in the cerebral tissue, these cells were shown to differentiate into anti-inflammatory patrolling monocytes [38]. Moreover, selective depletion of Lys6C^high^ was associated with increased hemorrhagic transformations through the rupture of neo-vessels within infarct peripheral zone [38]. Accordingly, Chu and co-workers recently demonstrated that pro-inflammatory monocytes infiltrating ischemic lesion were able to differentiate into M2 anti-inflammatory monocyte-derived macrophages, inducing the same translation to adjacent monocyte-derived macrophages or microglia [39]. A deeper spatial, temporal, and functional characterization of different mononuclear cell subtypes is needed to provide more clear translational evidences guiding future potential interventions.

### 2.3. T and B Lymphocytes

Antigen-specific and antigen-independent mechanisms were shown to activate T lymphocyte-mediated response after an IS [40,41]. T cells have been also detected in the infarction boundary zones early within 24 h after reperfusion, then they accumulate until day 7 and decrease in number after 14 days [42]. Among different T lymphocyte subsets, both CD4^+^ and CD8^+^ seem to play a harmful role in the infarcted parenchyma (Figure 2) [43]. Moreover, Shichita and colleagues showed in a murine stroke model that interleukin (IL)-17 plays an important role in the delayed phase of I/R injury during which apoptotic neuronal death occurs in the penumbra [44]. γδ T cells have been identified as the main source of this cytokine [44]. Similarly, also human infarcted brain showed high levels of IL-17 [45]. Thus, γδ T cells and IL-17 are interesting therapeutic targets to reduce neuronal death in I/R brain injury.

T regulatory (T_reg_) cells were suggested to play an opposite role from other T cells subsets. These cells are important regulators of immune homeostasis through direct interaction with other cells and production of anti-inflammatory cytokines, such as IL-10 and transforming growth factor (TGF)-β. The selective depletion of T_reg_ cells was associated with an increase in infarct size and behavioral deficits after brain ischemia in an experimental model of stroke, whilst beneficial effects of T_reg_ cells were mediated by IL-10 via abrogation of interferon (IFN)-γ-mediated pathways (Figure 2) [46].

B cell effects on I/R brain injury are mainly related to autoantibody production as detailed in the following paragraphs. Regulatory B cells showed the capacity to reduce inflammation and neurological deficits in a murine stroke model, while abrogation of this cell subset was accompanied by an enhanced brain infiltration by different leukocyte subpopulations (Figure 2) [47].

## 3. Soluble Mediators of Post-Ischemic Brain Injury

### 3.1. Cytokines

A variety of cytokines triggers the inflammatory response to ischemic brain injury. The most important, pro-inflammatory cytokines are TNF-α, IL-1, and IL-6, which were detected within the brain, the cerebrospinal fluid (CSF), and the blood of patients suffering from acute IS and are responsible for the extension of the infarct zone in animal models [48]. Many studies demonstrated that cytokine-mediated effects on ischemic area are strictly related to a huge increase of their levels, considering that infarcts reach their final volume many hours before cytokines peak [49,50]. It is very interesting to underline that even smaller increases in cytokine levels were shown to impact on the tissue injury evolution [48]. TNF-α owns a dual role: it can both exacerbate and reduce infarct evolution. TNF-α blocking with specific monoclonal antibodies confers neuroprotection [48]. On the other hand, TNF-α produced by microglial cells was protective on ischemic neurons [51].

IL-1 is a pro-inflammatory cytokine, which exists in two forms, IL-1α and IL-1β. Both forms signal through the IL-1 receptor type I, which can be competitively blocked by the receptor antagonist (IL-1Ra) [52]. Both IL-1α and IL-1β levels are elevated in the first hours following an IS. IL-1α is mainly secreted by microglia, while IL-1β is released by different compartments of the NVU [53]. It is surprising to note that IL-1 is not directly toxic, but it is able to activate astrocytes and endothelium favoring astrogliosis, release of chemokines, activation of metalloproteinase (MMP)-9, and release of vascular cell adhesion molecule (VCAM)-1 and intercellular adhesion molecule (ICAM)-1 [54,55,56].

IL-10 and TGF-β behave as anti-inflammatory cytokines, favoring the tissue recovery [57]. In particular, IL-10 was demonstrated to act as a down-regulator of the negative effects carried by pro-inflammatory cytokines modulating neuronal vulnerability to excitotoxicity induced by ischemia [58,59]. Blood levels of TGF-β increased the day after IS and remained elevated in astrocytes and microglia/macrophages for at least one week in animal models [60,61].

With regard to cytokine levels in CSF and bloodstream, the peak of TNF-α in CSF at 24 h after IS correlated with clinical outcomes. Other studies also indicated that increased levels at 6 h correlated with stroke severity [62,63]. On the contrary, IL-1β levels were increased in CSF after 6 h, but they did not correlate with infarct size or clinical outcome [63]. In the bloodstream, data on TNF-α and IL-1β levels were controversial [64].

The high heterogeneity of results on cytokine levels indicates that systemic inflammation has a poor clinical value to predict IS clinical evolution.

### 3.2. Chemokines

The three most relevant chemokines that might play a pathophysiological role after IS are: stromal cell-derived factor (SDF)-1, also known as C-X-C motif chemokine (CXCL)12; fractalkine, also known as C-X3-C motif chemokine ligand (CX3CL)1; and monocyte chemoattractant protein (MCP)-1, also known as C-C motif chemokine ligand (CCL)2.

The blockade of CXCL12 binding to its receptor (C-X-C chemokine receptor type 4) was demonstrated to reduce inflammation and infarct size in animal models. In particular, leukocyte infiltration and cytokine release were markedly reduced and BBB disruption was also limited [65,66]. Besides, in animal models CXCL12 expression demonstrated to be useful for the recovery [67]. CXCL12 was also shown to promote bone marrow-derived cell targeting to injured cells and improve local blood flow facilitating post-ischemic brain recovery [68].

Results from IS models demonstrated that the abrogation of the binding between CX3CL1 and its receptor (CX3CR1) on microglial cells leads to a reduced post-ischemic damage in terms of excitotoxicity, ROS release, BBB disruption, and leukocyte infiltration [69,70,71]. However, other studies highlighted beneficial effects correlating high plasmatic concentrations of CX3CL1 and good clinical outcomes [70,72]. Interestingly, Rosito and co-workers proposed that the release of chemokines, such as CX3CL1, CXCL16, and CCL2 affects neurons, microglia, and astrocytes during brain ischemia [73]. These molecules might be considered as endogenous self-protective compounds limiting the cell damage in the ischemic penumbra [73].

Antagonism and neutralization of the binding between CCL2 and its receptor (CCR2) was associated with a decrease in leukocyte infiltration within the infarcted area and a BBB impairment [74,75]. Strecker and colleagues hypothesized that the reduced permeability of BBB in *Ccl2*-knockout mice could be linked to transcriptional effects of CCL2 on BBB-related genes, such as occludin, zonula occludens-1, and zonula occludens-2 [76]. CCL2/CCR2 interaction was demonstrated to stop the hemorrhagic transformation via the recruitment of bone marrow-derived monocytes/macrophages and especially by preserving the integrity of the NVU via a TGF-β-triggered pathway [38]. Moreover, CCL2 was found to promote the migration of neuroblasts from neurogenic regions to ischemic damaged areas [77]. As for other chemokines, CCL2 owns detrimental properties, too. Indeed, CCL2 is involved in the recruitment of inflammatory cells exacerbating the brain injury [78,79].

### 3.3. Reactive Oxygen Species

**Figure 3:**
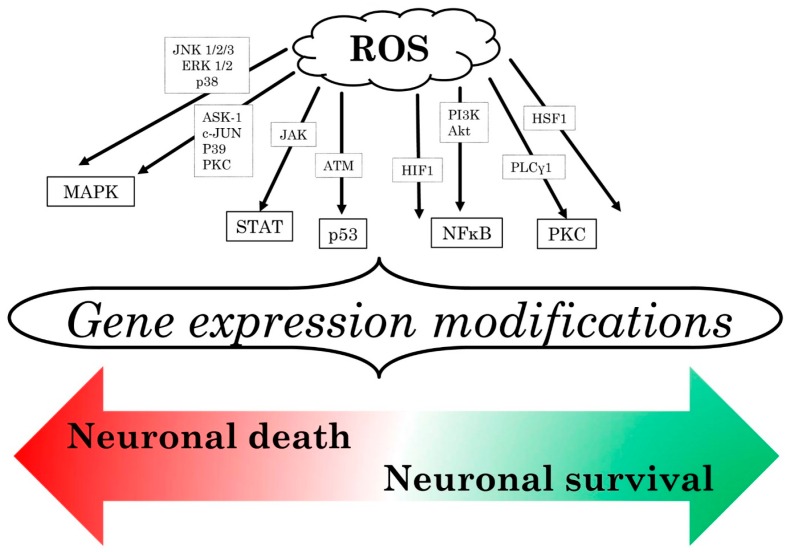
Estimated pathways mediated by oxidative stress. ROS can activate a great variety of cell signaling pathways within a neuronal cells with contrasting properties. Pathways involved in the promoting of cell death are represented by p38, c-Jun N-terminal kinase, p53, and extracellular signal-regulated kinase, while protective pathways are those mediated by phosphoinositide 3-kinase/Akt, hypoxia-inducible factor 1, and heat shock transcription factor 1. ROS: reactive oxygen species. JNK: c-Jun N-terminal kinase. ERK: extracellular signal-regulated kinase. MAPK: mitogen-activated protein kinase. ASK-1: apoptosis signal-regulating kinase-1. PKC: protein kinase C. JAK: Janus kinase. STAT: signal transducer and activator of transcription. ATM: ataxia telangectasia mutated. PI3K: phosphatidylinositol 3-kinase. NFκB: nuclear factor kappa-light-chain-enhancer of activated B cell. PCLγ1: phospholipase C gamma 1. HSF1: heat shock transcription factor 1. HIF1: hypoxia-inducible factor 1.

After an IS, the up-regulation of antioxidant enzymes was not described to efficiently counteract the huge increase in reactive oxygen species (ROS) generation. This redox unbalance triggers early inflammatory responses, involving both microglial and neuronal cells, as well as circulating leukocytes (Figure 3) [80]. ROS are essential mediators in the inflammatory environment whose major source is nicotinamide adenine dinucleotide phosphate hydrogen oxidase (NOX) [81] together with immune cells infiltrating ischemic tissue [82]. ROS generation stimulates ischemic neurons to release in turn additional ROS via other enzyme systems [83]. Different isoforms of NOX have been described in mammalians, which are the NOXs (NOX1-, NOX2-, NOX3-, NOX4-, and NOX5-containing oxidases) and the dual oxidases (DUOX1- and DUOX2-containing enzymes) [84].

NOX1 and NOX2 showed to enhance vascular pathology, while NOX4 contributed to vascular protection [85,86]. NOX1- and NOX2-derived ROS contribute to vascular disease mainly because of NO inactivation and oxidative damage. In fact, ROS are responsible for the inactivation of NO mediated by superoxide and also for the formation of peroxynitrite, causing irreversible damages to macromolecules, such as DNA, proteins, and lipids [86]. Moreover, peroxynitrite can interact with the endothelial nitric oxide synthase (eNOS) cofactor, tetrahydrobiopterin; in the absence of tetrahydrobiopterin, eNOS is enzymatically uncoupled and generates superoxide, contributing to increase the oxidative stress [84]. NOX2 deletion in mice was demonstrated to reduce the infarct size and the effect might involve both circulating neutrophils and Brain Res.ident cells [87]. The vasoprotective effect of NOX4 is not yet fully understood, but it might depend on the production of hydrogen peroxide. This product does not react with NO at the same manner as superoxide (produced by NOX1 and NOX2) and consequently does alter significantly its bioavailability. Moreover, NOX4 is considered an important signaling mediator capable to modify the function of some proteins by reversibly oxidizing reactive cysteine residues [88]. Finally, NOX4 overexpression was demonstrated to augment eNOS expression and activity in a catalase-dependent way promoting angiogenesis and recovery from hypoxia [89]. Nevertheless, post-stroke inhibition of NOX4 in mice has proved to be exceptionally neuroprotective, resulting in improved long-term neurological functions and reduced mortality [90].

Through a variety of upstream molecules, ROS activated different pathways leading to dual actions, either detrimental or beneficial. Once generated, ROS can cause microvascular occlusion and cross the vessel wall reacting with macromolecules finally leading to lipid peroxidation, protein oxidation, and DNA damage [91]. The phagocytic burst triggered by ROS is also responsible for the derangement of BBB explaining the typical post-ischemic complications, such as brain edema and hemorrhagic transformation. Neutrophils release a relevant amount of hydroxyl radical, which is blocked by hyperbaric oxygenation and by oxidant scavenger [92]. ROS of neutrophil origin are responsible for I/R injury; hydroxyl radical is able to sensitize the mitochondrial permeability transition pore activity enhancing calcium overload leading to the blocking of the mitochondrial respiratory chain and finally to neuronal death. Moreover, ROS are directly responsible for neuronal cell apoptosis via different inflammatory pathways as displayed in Figure 2. Microglial cells can generate ROS via NOX1 and NOX2 activation [93,94]. Astrocytes can reduce oxidative stress by the activation of the nuclear factor-erythroid 2-related factor 2-dependent pathway [95]. Finally, brain microvascular cells are capable of releasing ROS and triggering BBB dysfunction by lipid peroxidation [96].

### 3.4. Damage-Associated Molecular Patterns (DAMPs)

These endogenous molecules are released from injured cells after the ischemic trigger and induce the activation of Toll-like receptors (TLRs) and some others, such as receptor for advanced glycation end products (RAGE), and c-type lectin receptors, finally amplifying inflammatory mediator expression and tissue damage [97]. Mitochondrial DNA released by ischemic neurons is detected from immune cells as DAMPs and is capable to activate TLR9 [98]. Mitochondrial DAMPs include formyl peptides and mitochondrial DNA itself and are able to activate human neutrophils through formyl peptide receptor-1 and TLR9. Moreover, they stimulate neutrophil calcium influx and phosphorylation of MAPK, allowing neutrophil migration and degranulation both in vitro and in vivo. Maeda and colleagues demonstrated that intact mitochondria are released from cells undergoing necroptosis induced by TNF-α [99]. Human macrophages and dendritic cells have been shown to overwhelm mitochondria from necroptotic cells leading to macrophage secretion of cytokines and dendritic cell maturation [99]. Self RNA and DNA alongside with the LL37 peptide are able to activate an immune response through TLR7 and TLR9 [100]; TLR7 was also associated with a greater deterioration in IS with regard to TLR9 [101]. Both purines and their receptors, such as the P2Y receptor G protein-coupled receptor 17, are to be considered danger signals activating responses to tissue damage [102]. Also adenosine triphosphate can stimulate the activation of inflammasomes, whose activation leads to the amplification of post-ischemic injury and finally to neuronal death [103].

During lipid peroxidation, final products are yielded, such as phosphatidylcholine, malondialdehyde, and 4-hydroxynonenal. They are reactive aldehydes forming covalent adducts with primary amines of proteins and amino groups of lipids, such as phosphatidylethanolamine, thereby leading to the so-called oxidation-specific epitopes (OSEs). OSEs are usually recognized by different receptors in a hapten-specific manner activating adaptive immunity responses and for this reason have been proposed as a new type of DAMPs [104]. Phospholipids after oxidization have been considered DAMPs; they are CD36 ligand and have been described to promote inflammation via TLR2 in ischemic brain [105]. Oxidized phospholipids can also activate the inflammasome and two different pathways both ROS-dependent and ROS-independent have been implicated [106,107].

High mobility group box 1 (HMGB1) and peroxiredoxin (Prx) family proteins are two relevant DAMPs involved in brain ischemia. Typically, HMGB1 was described at the very early phases of stroke, whilst Prx was found at the subacute phase, mainly in the penumbral area. HMGB1 is a nuclear protein functioning as nucleosome stabilizer and transcription-factor like-protein. HMGB1 is actively secreted by macrophages, dendritic cells, and natural killer cells after acetylation through a mechanism partially depending on lipopolysaccharide (LPS)-mediated signaling through the TLR4-CD14 complex, TNF-α, and TGF-β. HMGB1 can also be passively released by necrotic cells following an injurious stimulus [108]. HMGB1 is used to interact with RAGE on endothelial cells, activating them and increasing VCAM-1, ICAM-1, and E-selection expression, thus facilitating neutrophil and monocyte adhesion to the endothelium and further neutrophil recruitment [108]. However, RAGE expression by endothelial cells can promote leukocyte recruitment through myeloid cells by the β2-integrin macrophage receptor 1 (CD11b). Also activated macrophages express HMGB1 facilitating their recruitment by binding RAGE at cell surface membrane [109]. HMGB1-RAGE signaling pathway has been confirmed as a critical player after brain injury as its inhibition was shown to improve the systemic metabolic and behavioral consequences of brain ischemia and reduce mortality without affecting the size of brain lesions [97]. Interestingly, Hu and co-workers investigated the HMGB1-RAGE pathway in diabetic rats, showing its increase in brain ischemia together with a decreased functional outcome [110]. Nonetheless, the injection of bone marrow stromal cells significantly reduced the expression of HMGB1 and RAGE and enhanced functional outcome, as confirmed by in vitro data [110].

Prx has been explained to own two opposing functions, both inside and outside brain cells. Ischemia increases Prx expression inside neural cells, favoring the survival of these cells by the ROS catabolism. When ischemic stress finally ends in necrosis, Prx is released from necrotic cells into the extracellular compartment and strongly stimulates macrophage infiltration via TLR2 and TLR4 inducing the inflammatory cytokine expression and the following ischemic injury. Over 24 h after stroke onset, IL-23 induces IL-17 production from T cells aggravating ischemic damage. In this view, HMGB1 can be considered a hyperacute DAMP, whereas Prx could act as secondary DAMP in the acute phase of stroke [111].

S100A8, S100A9, and cold-inducible RNA binding protein were described as DAMPs, even if with a lesser relevance, through the activation of TLR2, TLR4, and RAGE [112,113]. Heat shock protein gp96 is an endoplasmic reticulum chaperone for cell-surface TLR and was also considered as DAMP [114].

### 3.5. Autoantibodies

Very little is known about autoantibodies in the setting of IS. Most autoantibodies produced after a central nervous system (CNS) injury are considered “natural” autoantibodies that could bind CNS antigens. In fact, B1 cells are able to synthesize immunoglobulin (Ig)M autoantibodies binding multiple antigens with low affinity, which are responsible for the clearing of aging and damaged cells [115]. Moreover, natural IgM autoantibodies have been described to improve re-myelination and block neuronal apoptosis in animal models of multiple sclerosis [116]. Many autoantigens and autoantibodies have been taught to cause or exacerbate neuronal injury during brain diseases. A possible explanation for the production of autoantibodies following IS can derive from the neurological damage induced by ischemia triggering a secondary immune response toward injured tissues or their components. The prerequisite for antibodies to contribute to brain damage is the access to CNS, which could occur during events accounting for a dysfunction of BBB, such as IS, hypertensive crisis, or lacunar stroke. Bornstein and co-workers showed that autoantibodies to specific antigen, such as neurofilaments (NF) but not to the ubiquitous antigen cardiolipin rose significantly in the first three months following an IS [117]. This would be possible because antibodies toward brain-specific antigens could be preferentially induced after IS on the basis of a different degree of immunologic tolerance. The clinical relevance of antibodies to NF is not fully understood, but they have been demonstrated to play a role in the development of human primary degenerative dementia, so that a similar role could be played in the stroke-induced dementia [118]. Antibodies binding to subunits (NR2A/2B) of *N*-methyl-d-aspartate receptor, too, have been found to be increased in patients with IS and transient ischemic attack [119]. Interestingly, Kimura and colleagues showed a significant correlation between anti-heat shock protein (Hsp) 60 antibody titer and the severity of white matter hyperintensities on brain magnetic resonance, suggesting a potential role for Hsp60 as an active factor underlying cerebral small-vessel disease [120]. Myelin basic protein (MBP) is a myelin-associated protein found in oligodendrocytes. A powerful immune response toward MBP was triggered by Th1 lymphocytes following an IS and was demonstrated to correlate with a poor clinical outcome at 90 days, even when adjusted for National Institute of Health Stroke Scale (NIHSS) score and age [121]. Shibata and colleagues evaluated Ig titers in patients with IS, noting that stroke was associated with a quick decrease in the titer of anti-tetanus toxoid IgG antibodies; that patients with white matter disease showed higher IgG antibodies titers to MBP and proteolipid protein; and finally confirmed the above-mentioned findings by Becker and co-workers [121], showing worse long-term outcome in association with high antibody titers to MBP. This latter evidence is very interesting suggesting the real possibility that some antibodies do play a pivotal role in the pathophysiology of IS [122].

### 3.6. Miscellaneous: Osteoprotegerin, Adipokines, and Osteopontin

Osteoprotegerin (OPG) is a secreted protein binding to receptor activator of nuclear factor-κB ligand (RANKL) and inhibits RANK/RANKL pathway [123]. The OPG/RANK/RANKL axis plays a role in bone metabolism and arterial calcification deposition [124]. Furthermore, it is expressed in different immune cells suggesting an involvement in inflammation regulation [125,126]. OPG plasma levels are higher in patients with IS compared with healthy individuals [127]. Moreover, OPG levels are positively correlated with stroke severity and worse prognosis [127,128]. Guldiken and co-workers pointed out a difference in OPG levels between IS subtypes, being significantly more elevated in large-vessel disease than in small-vessel disease group [129].

Adipokines were shown to play a central role in vascular inflammation and seem to be involved in the pathophysiology of several cardiovascular events, even in IS [130]. Serum levels of adiponectin and leptin had differential association patterns with IS subtypes [130]. In a recent study by our group, leptin levels increased rapidly after an IS. According to a previous study [131], our group was able to suggest a neuroprotective effect of leptin in terms of clinical and radiological outcomes in humans [132]. These clinical observations were supported by experiments using a rodent model of IS, in which microglia and macrophages of the penumbra were shown to release leptin [133]. Leptin may reduce cerebral injury after ischemia throughout other already identified mechanisms. Firstly, leptin administration increased glucose uptake in neurons by the activation of phosphatidylinositol 3-kinase (PI3K)/Akt signaling pathway [134]. Secondly, an increase in neurogenesis and angiogenesis in the perilesion cortex was observed after leptin treatment [135]. Thirdly, leptin was found to reduce oxidative stress caused by the overproduction of ROS after reperfusion [136]. After peripheral leptin administration, an increase in the level of anti-apoptotic Bcl-2 protein and a decrease in the level of pro-apoptotic caspase-3 protein were seen [136].

Also adiponectin (APN) seems to have a neuroprotective role after IS. In a prospective study by Efstathiou and co-workers, patients with lower APN levels showed worse outcomes after IS irrespective of the presence of other adverse factors [137]. On the other hand, APN levels were inversely correlated with the extent of infarcted area [137]. In animal models, APN overexpression reduced cortical atrophy after an IS and increased focal angiogenesis by promoting adenosine monophosphate-activated protein kinase signaling [138,139]. Finally, APN overexpression was demonstrated to improve neurobehavioral outcomes, even in aged mice [138,139].

Osteopontin (OPN) is a secreted glycoprotein involved in the bone turnover and metabolism [140]. However, in the last decades, it has been investigated for its pro-inflammatory activity in chronic inflammatory and cardiovascular diseases [140]. Several clinical studies on IS patients found a positive correlation between OPN plasma levels and adverse long-term outcomes in terms of neurological disabilities and infarct volume [141,142]. Although an active role of OPN in post-ischemic injury was suggested, some in vivo and in vitro studies indicate a neuroprotective effect [143,144]. OPN was shown to reduce ischemic cell death by the phosphorylation of Akt and the activation of PI3K/Akt pathway [143]. In a recent study, the main source of OPN expression has been identified in monocytes/macrophages infiltrating the infarct border area. Indeed, OPN was shown to play a role in astrocyte polarization toward the lesion core, a key phase for BBB repair and resolution of vasogenic edema [145].

## 4. Inflammatory Mediators as Potential Diagnostic or Prognostic Biomarkers

### 4.1. Clinical Evidence

**Figure 4:**
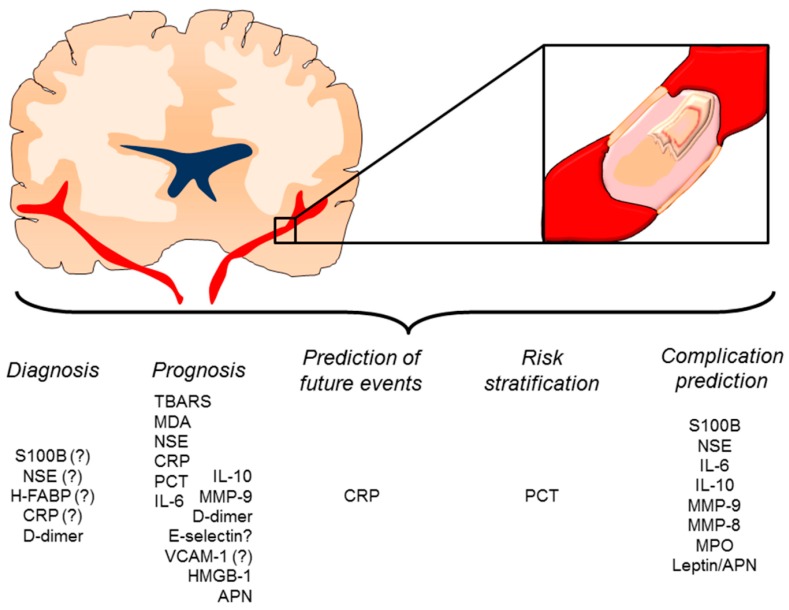
Diagnostic and prognostic markers in ischemic stroke. APN: adiponectin. CRP: C-reactive protein. H-FABP: heart-type fatty acid binding protein. HMGB: high mobility group box. IL: interleukin. MDA: malonildyaldeide. MMP: matrix metalloproteinase. MPO: myeloperoxidase. NSE: neuron-specific enolase. PCT: procalcitonin. TBARS: thiobarbituric acid reactive substances. VCAM: vascular cell adhesion molecule. “?” stands for putative or uncertain role.

As early diagnostic accuracy of brain computed tomography scan is only about 85%, many studies in the last few years attempted at investigating the prognostic ability of inflammatory biomarkers toward IS diagnosis as well as response to treatment and prognosis. However, an individual biomarker validated for clinical use has not been identified yet. To be clinical useful an IS biomarker must be sensitive to early ischemia and relatively specific for the brain. A good diagnostic biomarker should also distinguish between different IS etiologies and exclude other conditions mimicking stroke, such as hypoglycemia, focal seizures, migraine, and psychogenic diseases. Furthermore, specific challenges regarding biomarkers of central nervous tissue include the penetration of BBB and the lack of correlation between functional symptoms and volume (as opposed to location) of injured tissue. According to pathophysiological processes underlying IS, potential inflammatory biomarkers may be classified as released by: (i) astroglial activation and neuronal injury (Table 1) [146,147,148,149,150,151,152,153,154,155,156,157]; (ii) systemic inflammatory response (Table 2) [158,159,160,161,162,163,164,165,166,167,168,169,170,171,172,173,174,175,176,177,178,179]; (iii) dysfunctional endothelium (Table 3) [180,181,182,183,184,185,186] (Figure 4). 

BBB breakdown after IS allows the permeability of proteins released by astrocytes and neurons into the bloodstream [187]. Therefore, the measurement of these proteins might potentially be useful as specific biomarker of IS (Table 1). Among them, the release of calcium-binding proteins S100B has been traditionally ascribed to astrocyte dysfunction, but recent studies rather indicate a role as a marker of BBB injury [188,189]. Serum levels of S100B increase at 8–10 h from symptom onset, reach the peak after 72 h and then drop in levels at 96 h [146]. However, a recent meta-analysis of 10 pooled case-control studies failed to validate S100B as diagnostic marker of IS [147]. Instead, S100B was shown to predict hemorrhagic transformation (HT) and long-term functional outcome [146,148]. Lipid peroxidation and oxidative stress have been described as early events of neuroinflammation and then investigated as prognostic markers of IS. The thiobarbituric acid reactive substances (TBARS) and the direct assay of malonildyaldeide (MDA) have been identified as predictors of poor neurological outcome [149,150]. However, TBARS and MDA lack of specificity and their measurement is highly imprecise, likely resulting in an overestimation of oxidative stress. Furthermore, the reliability of redox balance assay is weakened by confounding factors (lifestyle, comorbidity, drug administration) and methodological limitations involving sample handling, storage, and preparation [190]. The intracytoplasmic glycolytic enzyme neuron-specific enolase (NSE) and the heart-type fatty acid binding protein (H-FABP) are characterized by great specificity for brain tissue and early rise during the first 3 h after the symptom onset [191,192,193]. However, the diagnostic accuracy of NSE and H-FEBP is affected by a low sensitivity and their use is not recommended in clinical practice. NSE also correlated with stroke severity at admission [151,152,154] and HT [155], whereas data on long-term outcomes are controversial [153,156].

Inflammatory molecules secreted by NVU may be released in the bloodstream, thus activating the immune system. Therefore, also systemic inflammatory mediators may contribute to IS diagnosis and risk stratification (Table 2). Increased C-reactive protein (CRP) concentration represents a well-known predictor of first/recurrent cerebrovascular events [168,194], but whether it also serves as a prognostic factor has not yet been established. Early increase of CRP occurs in IS and in many other inflammatory conditions as well, thus reflecting the low specificity of this biomarker. Furthermore, various studies suggested a potential role of CRP as predictor of worse neurological outcome, but this evidence remains controversial [158,159,160,161,162,163,164,167,168,195]. In this regard, a recent systematic review from 46 studies recognized CRP as independent predictor of long-term functional outcome in only 13 studies [196]. The high variability in the study design and the lack of classification/discrimination over clinical variables further challenge the clinical use of CRP, at least as single biomarker. Noteworthy, procalcitonin showed better accuracy in predicting 1-year mortality after IS as compared to CRP [163,166]. As this association was confirmed even after adjusting for stroke severity, it seems that procalcitonin may provide additional general prognostic information. Indeed, procalcitonin was demonstrated to correlate with IS severity at admission as well as to predict worse long-term neurological outcome [165,166]. Similarly, the predictive value of cytokine release remains to be fully understood. Up-regulation of TNF-α, IL-1β, IL-6, and IL-10 has been largely investigated in experimental studies, whereas over time variations in human serum have been described in several cohorts. However, data on peripheral cytokine release are extremely variable, likely due to the difference in IS severity, location, age, co-morbidities, and systemic inflammatory status at admission. Furthermore, this variability is of paramount importance in case-control study design [197]. According to those limitations, IL-6 has been suggested as promising biomarker of short- and long-term worse neurological outcome in different studies [169,170,175]. However, a recent meta-analysis pooling 24 longitudinal studies emphasized the low prognostic accuracy of IL-6 [171]. Interestingly, IL-6 has also been shown to predict the occurrence of infection in patients suffering from an IS [172,174]. Conversely, the anti-inflammatory cytokine IL-10 showed a different time course, characterized by a delayed increase over 72 h post-IS [198]. Consistent with experimental evidence, high levels of IL-10 has been recently associated to good functional outcome [176], but also an increased risk of post-IS infections has been described [177]. Despite those promising evidence, the assay of single inflammatory mediator does not reflect the heterogeneity and complexity of systemic immune response. Rather, development of panels including different biomarkers might be more representative and then useful for clinical practice. Furthermore, few studies have assessed the time line of cytokine release and how their balance affects neurological outcome. Understanding this balance might be critical for better defining the time window for cytokine assay.

Only recently the impact of neutrophil degranulation has been investigated [199]. Inzitari and co-workers first indicated the pre- and post-thrombolysis variations of serum MMP-9 as predictor of HT and worse long-term outcome, defined as death and poor modified Rankin scale score [179]. More recently, our research group confirmed the neutrophil burst induced by thrombolysis, also linking the neutrophil release of MMP-9, MMP-8, and myeloperoxidase (MPO) to the occurrence of HT in the first day after IS [178].

As athero-thrombotic disease, both coagulation/fibrinolysis cascade and endothelial dysfunction contribute to IS pathophysiology [180]. Especially D-dimer was effective in identifying IS etiology [181,182] and showed good prognostic accuracy as predictor of short- and long-term worse outcome [182,183,185,186]. Similar results were also reported for E-selectin and VCAM-1, but those data required further validation in additional large-cohort studies [184].

### 4.2. New Candidate Inflammatory Biomarkers

The release of endogenous alarmins—also referred to as DAMPs—from necrotic cerebral cells has been indicated as early promoter of local and systemic inflammatory cascade [200]. Among them, several studies in the last years investigated the HMGB1 in human stroke patients as a pro-inflammatory biomarker (Table 4) [132,201,202,203,204,205]. Persistent increase of serum HMGB1 occurs early after IS and lasts over 30 days. HMGB1 correlates with circulating leukocytes and promotes the release of inflammatory mediators [206,207].

Similarly, adipocytokines recently emerged as inflammatory mediators potentially associated with the risk of stroke [208,209], but whether they have a role in stroke pathophysiology has not yet been established. In two studies, APN failed to predict long-term outcome, but was shown to correlated with IS etiology and short-term neurological disability [204,205]. Instead, our research group highlighted the potential role of leptin/APN ratio as potential strategy for prediction of early HT and long-term worse outcome [132].

## 5. Anti-Inflammatory Treatments in IS: Evidence from Pre-Clinical Studies

**Figure 5:**
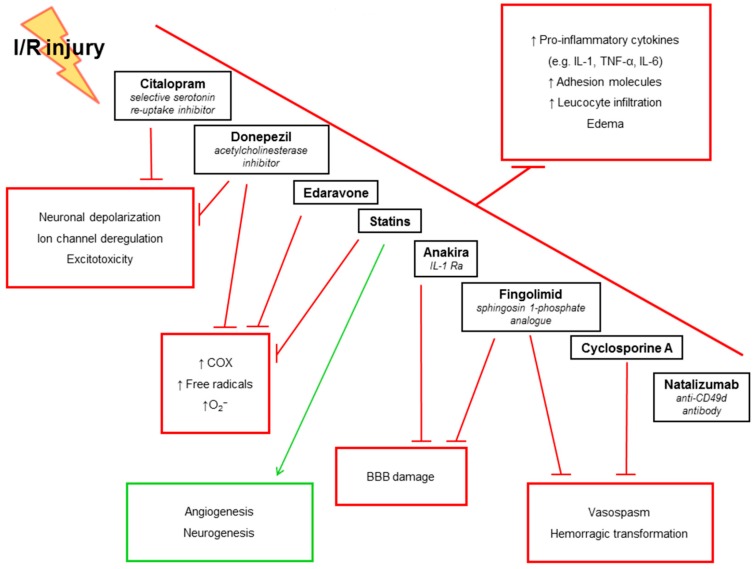
Anti-inflammatory treatments in stroke pathophysiology. Inflammatory and immune-mediated mechanisms of neuronal injury have been fully investigated in last years. As a result, many drugs modulating these pathways have been tested initially in animal models of stroke and then in humans. Neuronal depolarization and excitotoxicity are main targets for citalopram and donepezil. Edaravone mainly acts as a reactive oxygen species scavenger, while statins share several mechanisms with other drugs and furthermore promote angiogenesis. BBB: blood-brain barrier; CD: cluster of differentiation; COX: cyclooxygenase; IL: interleukin; O2^−^: superoxyde ion; TNF: tumor necrosis factor; Ra: receptor agonist.

As inflammatory and immune-mediated mechanisms of neuronal injury have received greater attention, many drugs have been developed or tested if used in other settings (Figure 5). Several treatments modulating inflammatory and immune responses to brain ischemia have entered pre-clinical trials with different and sometimes contrasting results (Table 5) [10,210,211,212,213,214,215,216,217,218,219,220,221,222,223,224,225,226,227,228,229,230,231,232,233,234,235,236,237,238,239,240,241,242,243,244,245,246,247,248,249,250,251,252,253].

### 5.1. IL-1Ra

IL-1 has been shown to play a central role in brain injury since long time. Beneficial effects of IL-1Ra in stroke are not fully cleared, but existing data suggest neuroprotective and vasculoprotective effects. In particular, neuroprotective effects are greater after co-administration with excitotoxins in the rat brain, whilst vasculoprotective effects are mainly linked to the prevention of BBB impairment and the blocking of neutrophil migration [254].

Treatment with recombinant IL-1Ra in rats with permanent middle cerebral artery occlusion (MCAO) was described to be protective, both after central and peripheral administration [255,256]. In a temporary MCAO model of rats, the intra-cerebroventricular injection of the anti-IL-1β antibody was also found to limit the ischemic injury [247]. Interestingly, delayed administration of IL-1Ra in rats was noted to reduce infarct volume after permanent MCAO, as peripheral administration did, too [236,257]. Transgenic mice further increased the evidence of an essential role for IL-1 in stroke; in fact, the genetic deletion of IL-1α and IL-1β in rodents assured a marked reduction in lesion volume during brain ischemia in experimental stroke [258]. The first meta-analysis and systematic review on IL-1Ra effectiveness in IS dates back to 2009, confirming protective effects and underscoring the lack of studies using systemic administration, delayed treatment, and animals with important comorbidities [259]. These concerns were addressed and IL-1Ra after delayed subcutaneous administration in corpulent rats confirmed its efficacy [234]. Apart from corpulent rats, those with pneumonia or treated with LPS and aged animals were tested, but these comorbidities did not alter the heterogeneity, the efficacy of IL-1Ra being maintained; nevertheless, the efficacy must still be tested in animals with hypertension and other than rodents [260]. IL-1Ra was also demonstrated to enter the brain parenchyma at therapeutic doses after systemic administration both after transient and permanent MCAO in rats, providing long-term functional recovery [234]. Interestingly, IL-1Ra was recently confirmed to maintain its protective effects for more than 20 years [261]. Finally, three phase II randomized clinical trials (RCTs) have been completed, one is still ongoing, and another is planned to start in 2018; no phase III trials are currently under development. The two completed studies showed it is well tolerated and no safety concerns were highlighted [261,262]. Current phase II trials in the United Kingdom are investigating subcutaneous IL-1Ra, which maintained its efficacy [260].

### 5.2. Statins

Since long time, statins were demonstrated to exert neuro-protective effects on ischemic brain by the selective up-regulation of eNOS. Prophylactic therapy with statins increased cerebral blood flow, reduced infarct size, and enhanced clinical outcomes in normocholesterolemic mice. The blood flow and neuroprotective effects of statins did not take place in eNOS-deficient mice, indicating that improved eNOS activity by statins represents the main mechanism of neuroprotection [219]. In stroke-prone spontaneously hypertensive rats chronically treated with cerivastatin, the incidence and the size of stroke were significantly reduced as well as stroke-associated symptoms and early mortality; interestingly, statin treatment significantly decreased superoxide production from non-stroke parenchyma of brain and infiltration of inflammatory cells to injured tissues, confirming the neuroprotective effects of this drug class [222]. These findings were confirmed also with mevastatin and rosuvastatin upregulating eNOS and enhancing cerebral blood flow [211,235,263]. Moreover, in a mouse model of embolic focal ischemia after long-term (14 days) simvastatin, atorvastatin or vehicle treatment, ischemic lesion volume and neurologic deficits were significantly decreased by both simvastatin and atorvastatin, which increased eNOS and tissue-type plasminogen activator (t-PA) mRNA levels without changes in mRNA levels of plasminogen activator inhibitor-1. While in eNOS knockout mice atorvastatin was shown to reduce the volume of ischemic injury and enhance neurologic outcomes after arterial embolic occlusion, statins were not protective in t-PA knockout mice, confirming the beneficial effects of statins in IS by both eNOS and t-PA [212]. Atorvastatin and simvastatin were described to enhance functional outcomes and brain plasticity in IS rats [215]. In particular, atorvastatin treatment exhibited significant increases in vascular endothelial growth factor, cyclic guanosine monophosphate, synaptophysin and stimulated angiogenesis, endogenous cell proliferation, and neurogenesis, indicating an essential role for atorvastatin in inducing post-stroke brain plasticity. Treatment with simvastatin in rats after MCAO blocked the increase in brain infarct volume after 24 h and induced a consistent reduction after 48 h; the same beneficial effect was observed when simvastatin was administered before the induction of focal ischemia and confirmed by magnetic resonance imaging (MRI). The neuroprotective effects of simvastatin were coupled to an increase in eNOS immunoreactivity found in simvastatin-treated rat brain [242]. In simvastatin-pre-treated cultured human brain microvascular endothelial cells (BMECs) then subjected to oxygen glucose deprivation (OGD), the expression of MMP-2 was reduced, whilst MMP-9 synthesis rate was low and unaffected by simvastatin treatment. Tissue inhibitor metalloproteinase (TIMP)-1 and TIMP-2 gene expression and protein secretion were both strongly induced, demonstrating the positive effect of simvastatin on MMP metabolism in human BMECs and experimental stroke [238]. A systematic review and meta-analysis by Baryan and co-workers confirmed the neuroprotective effects of statins in animal post-stroke recovery in terms of both reduction of infarct size and improvement of neurological outcome [264]. A more recent meta-analysis involving non-industry-sponsored preclinical studies confirmed the previous data, showing that the effect of statins was significantly larger for studies sponsored by non-industry sources than studies sponsored by industry [265].

### 5.3. Fingolimod (FTY720)

Fingolimod is a sphingosine 1-phosphate receptor analogue preventing lymphocyte way out from lymphoid organs [266]. Studies on mouse models showed that fingolimod was able to decrease cerebral lymphocyte infiltration, limit the hemorrhagic transformation and the BBB breakdown, and reduce the levels of ICAM-1, IFN-γ, and IL-17 [214,239]. A systematic review advised to consider with caution fingolimod as a potential drug for IS as more good quality experimental studies evaluating its safety as well as its efficacy in old animals mimicking human comorbidities were needed [267]. Induction of lymphocytopenia and reduction of microvascular thrombosis seemed to be the main mechanisms of action of fingolimod [268].

### 5.4. Donepezil

Donepezil is a central acetylcholinesterase inhibitor. In different animal models, donepezil was demonstrated to decrease ischemic injury probably via the nicotinic acetylcholine-receptor signaling and the Kv2.1 potassium channels [231,244,252]. Other neuroprotective properties of donepezil have been described, such as the attenuation of the excitotoxic damage induced by membrane depolarization, the improvement of cognitive dysfunction after chronic cerebral hypoperfusion, and the reduction of systemic inflammatory responses via the blocking of cytokine release and cyclooxygenase-2 expression in the brain [210,225,269].

### 5.5. Citalopram

Citalopram is a selective serotonin re-uptake inhibitor mainly used as antidepressant drug. In mice, citalopram was demonstrated to improve neurovascular regeneration and functional recovery following IS [220]. The main explanation of these effects could be its anti-inflammatory effects, as demonstrated in a focal brain ischemia rat model in which citalopram was able to down-regulate various inflammatory cytokines, such as IL-1β and TNF-α [270]. Delayed degeneration of dopaminergic neurons following brain ischemia was demonstrated to be reversed by citalopram [223]. Citalopram was described to reduce glutamate and d-serine release from LPS-activated microglia leading to an improvement in the survival of OGD-injured cortical neurons [271].

### 5.6. Natalizumab (Anti-CD49d Antibody)

Natalizumab is a humanized monoclonal antibody recognizing the α4 integrin currently used in multiple sclerosis and Crohn’s disease. In 2001, for the first time, beneficial effects of anti-CD49 d antibody both before or immediately after MCAO were reported showing a reduction of the volume of the ischemic lesion and an improvement of the neurological outcome [213,237]. In stroke models, very late antigen (VLA)-4 blockade before or after 3 h after induction of cerebral ischemia was found to improve the outcome because of a reduced lymphocyte invasion in the brain and neurotoxic cytokine production. Furthermore, VLA-4 inhibition resulted in a reduction of the post-ischemic VCAM-1 up-regulation. These findings supported the concept that invading T cells via their humoral secretion (IFN-γ) and cytotoxic mechanisms (perforin) played a pivotal role in the pathways leading to delayed post-ischemic tissue injury [226]. On the contrary, Langhauser and colleagues demonstrated that pharmacological inhibition of the VLA-4/VCAM-1 axis with anti-CD49d antibody in experimental stroke failed and raised doubts on the effectiveness of anti-CD49d as a stroke treatment [224]. Different studies supported the pathogenic role of infiltrating T-cells as their blockage could be a turning point for the neuroprotective effects of anti-CD49d antibody without considering other cell types as possible relevant players. For this reason, Neumann and colleagues showed that the beneficial effects of post-stroke anti-CD49d therapy could be brought by the inhibition of both neutrophils and lymphocytes trafficking into the brain parenchyma [232]. Llovera and co-workers performed a preclinical randomized controlled multicenter trial in order to evaluate the efficacy of anti-CD49d treatment and found different effects according to different stroke models [230]. In particular, the blockage of CD49d decreased infarct size in animals suffering from permanent MCAO, but did not influence the lesion in animals suffering from a transient MCAO [230].

### 5.7. Cyclosporine A

Cyclosporine A owns immunosuppressive activity, but can also reduce infarct size in animal models of brain ischemia, markedly ameliorating delayed neuronal damage as it passes the BBB, maybe with the involvement of calcineurin [217,243,250,253]. Cyclosporine A was demonstrated to block the way of the nuclear factor of activated T cell family group of transcription factors to the nucleus leading to a specific inhibition of IL production in T cells [272]. In rodent models, cyclosporine A was found to reduce ischemic cerebral lesions, ROS production, and cerebral vasospasm following subarachnoid hemorrhage [217,273]. Moreover, cyclosporine A was described to block T-cell activation and cytokine production.

### 5.8. Edaravone (MCI-186)

Edaravone is an antioxidant and ROS scavenger. In a rat transient focal ischemia model, edaravone showed a minor infarct volume and edema compared with control group after 24 h from stroke. Besides, it was able to decrease IL-1β and MMP-9 levels as well as many others, such as fractalkine, IL-1α, IL-1Ra, IL-6, IL-10, macrophage inhibitory protein (MIP)-1α and MIP-3α [221]. These beneficial effects in terms of infarct volume and hemorrhagic transformation decrease were demonstrated also in IS rats with hyperglycemia [274]. Edaravone was found to greatly accelerate thrombolysis by alteplase in an experimental thrombosis model [248]. In another experimental thrombotic stroke, Sun and colleagues found that t-PA at 0.5 or 1 h reduced infarction volume showing both synergistic and additive benefits with edaravone treatment [275]. The acute, combined treatment led to more than 50% mortality reduction, nearly 80% infarct size decrease, strong white-matter protection, vascular reperfusion improvement, oxidative stress, inflammatory cytokine, and matrix metalloproteinase activity reduction [275]. In a rodent model of stroke, tempol and edaravone were tested to evaluate the extent of glutamate and aspartate release. Tempol, but not edaravone, reduced the amino acid release by 60%–80% as well as an intra-cerebroventricular injection of tempol, but not edaravone, decreased infarction size by nearly 50% improving neurobehavioral outcomes. Interestingly, in vitro assays demonstrated that tempol was superior in eliminating superoxide anion, whilst edaravone was more efficient in scavenging hydrogen peroxide, hydroxyl radical, and peroxynitrite [276]. In a rat model of transient cerebral I/R, the combination of edaravone and borneol significantly improved ischemic injury demonstrating a synergistic effect, which led to reduced levels of pro-inflammatory mediators and free radicals. This combination showed a therapeutic time window of 6 h in the present model and significantly ameliorated injury also in permanent ischemia model. Surprisingly, the combination triggered long-term effect, such as improvement in elemental vital signs, sensorimotor functions, and spatial cognition [245].

## 6. Anti-Inflammatory Treatments in IS: Evidence from Clinical Trials

In light of the beneficial effects of some anti-inflammatory treatments experimented in animal models, some of these compounds were tested in humans in phase II or III clinical trials in the setting of IS (Table 6) [277,278,279,280,281,282,283,284,285,286,287,288,289,290,291,292,293,294,295].

### 6.1. IL1-Ra

Anakinra is the recombinant form of human IL-1Ra and has a half-life of 5–6 h. In a phase II RCT, patients within 6 h of the onset of symptoms of IS were randomized to anakinra or placebo intravenously by a 100 mg loading dose over 60 s, followed by a 2 mg/kg/h infusion over 72 h [283]. Neutrophil and total white cell counts, CRP, and IL-6 concentrations were lower in anakinra-treated patients; moreover, among patients with cortical infarcts, clinical outcomes at 3 months in the anakinra-treated group were better than in placebo-treated. Over the follow-up period, no adverse events were recorded. In secondary analyses, Smith and colleagues investigated whether the administration of IL-1Ra to patients with IS affected innate cellular immune responses [294]. Induction of TNF-α, IL-1β, IL-6, IL-8, and IL-10 by LPS was significantly reduced in patients at admission, prior to treatment with anakinra or placebo, with respect to controls. While in the placebo group the cytokine induction remained suppressed over the entire week of follow-up, in anakinra-treated patients the suppression of this induction was reversed. The authors concluded that IL-1Ra might be able to reverse peripheral innate immune suppression in the acute phase of IS by blocking IL-1-correlated pathways. Currently, the Subcutaneous interleukin-1 receptor antagonist (SC IL-1RA) in Stroke Study, a phase II RCT, is investigating the effects of anakinra subcutaneously in the setting of IS (available at: http://www.isrctn.com/ISRCTN74236229). The recruitment started in November 2013 and ended in April 2016 with participants receiving anakinra subcutaneously twice daily for 72 h or placebo; the first injection of IL-1Ra was given within 6 h from stroke onset with 5 more doses at 12 h intervals for 3 days. The primary endpoint was to evaluate the reduction in inflammatory biomarker levels between 6 h and 5–7 days after stroke. Finally, canakinumab is a human monoclonal antibody selectively targeting IL-1β with a half-life of 21–28 days, which promisingly gains great interest as treatment for IS patients [296].

### 6.2. Statins

Beneficial effects of statins in atherosclerosis are by now well known. Beyond cholesterol lowering, statins have been shown to own many anti-inflammatory and antioxidant activities by modulating inflammatory pathways involving kinase phosphorylation and protein prenylation [297]. In a *post* hoc analysis of the Scandinavian Simvastatin Survival Study, simvastatin was demonstrated to reduce by 30% the rate of strokes and transient ischemic attacks [277]. Pravastatin was found to reduce by nearly 30% stroke incidence after myocardial infarction, with a reduction involving all kind of strokes and a similar treatment effect when adjusted for age, sex, history of hypertension, cigarette smoking, diabetes, left ventricular ejection fraction, and baseline cholesterol and triglyceride levels [290]. In a more recent study, simvastatin was tested in the acute phase of IS (3–12 h from symptom onset) showing improvement in neurological scales at several time-points, even if no clear mechanism was found due to the lack of effect on analyzed biomarkers (IL-6, IL-8, IL-10, MCP-1, ICAM-1, VCAM-1, CRP, selectins, and TNF-α) [288]. Collins and co-workers showed that simvastatin 40 mg daily was highly effective in reducing the rate of ischemic strokes in patients with cerebrovascular disease or another occlusive arterial disease or diabetes; the reduction in stroke occurrence was significant by the end of the second year [298]. Recently, Montaner and co-workers did not show any improvement in neurological or functional outcome when patients were treated with simvastatin [299]. In addition, in this study, the combination therapy of simvastatin with tPA in acute IS was proven to be safe, showing low rates of bleeding complications [299]. In the Anglo-Scandinavian Cardiac Outcomes Trial-Lipid Lowering Arm, 19,342 hypertensive patients with normal lipids and at least three cardiovascular risk factors were administered atorvastatin 10 mg daily or placebo, but the trial was early stopped after 3.3 years because of great benefits in the treatment arm, that is a 27% reduction of fatal and non-fatal strokes [292]. In the Stroke Prevention by Aggressive Reduction in Cholesterol Levels trial, patients with recent stroke or transient ischemic attack without known coronary heart disease were administered atorvastatin 80 mg daily showing a reduction in stroke incidence and overall cardiovascular events, by 16% and 10% respectively, despite a small increase in the incidence of hemorrhagic stroke [278]. The Long Term Intervention with Pravastatin in Ischemic Stroke Study included patients with myocardial infarction/unstable angina and an initial total cholesterol of 155 to 270 mg/dL under pravastatin 40 mg daily or placebo. After a 6 year-follow-up, pravastatin demonstrated a 23% relative reduction in risk from non-hemorrhagic stroke [300]. In the Myocardial Ischemia Reduction with Aggressive Cholesterol Lowering sub-study, intensive treatment with atorvastatin over 16 weeks in patients with acute coronary syndromes was found to decrease the overall stroke rate by half without causing hemorrhagic stroke [301]. The PROspective Study of Pravastatin in the Elderly at Risk trial and the GREek Atorvastatin and Coronary-heart-disease Evaluation trial showed more weak results [293,302]. In a predetermined pooled analysis of almost 2000 patients with evidence of atherosclerosis and elevated lipid levels, pravastatin was found to reduce by 62% the stroke rate [303]. In the JUPITER trial, apparently healthy people without hyperlipidemia and with elevated high-sensitivity CRP levels were administered rosuvastatin 20 mg daily highlighting a significant reduction in the incidence of major cardiovascular events (33% reduction in the primary end point); in particular, the effect for stroke was very consistent (hazard ratio 0.52, 95% confidence interval 0.34–0.79, *p* = 0.002) and was observed in all subgroups evaluated [291]. Recently, rosuvastatin 20 mg daily demonstrated to have poor effects in reducing the recurrence of IS in statin-naïve patients. No difference between rosuvastatin and placebo was found for the frequency of new ischemic lesions and infarct volume growth at 5 days on MRI, although hemorrhagic complications occurred less frequently in the rosuvastatin group [304]. Premorbid statin users have been shown both less frequent plaque enhancement and lower enhancement volume, which is a marker of plaque instability. Interestingly, lower degree of plaque enhancement together with antecedent statin use were mainly correlated to a higher frequency of non-embolic lesion pattern, suggesting that statin therapy could reduce large cortical infarcts by stabilizing intracranial atherosclerotic plaque. Moreover, the proportion of IS due to plaque rupture was lower among premorbid statin users. Unfortunately, no evaluation was done with regard to the impact of statins on the reduction of the incidence of IS in patients with intracranial atherosclerosis and no clinical improvement was highlighted among premorbid statin-treated patients at 90 days after IS [305]. Many observations demonstrated that pre-treatment with statins improves stroke outcome at 90 days and this benefit is evident across all the stroke subtypes [306,307], as confirmed by the meta-analysis by Biffi et al., especially in patients with small vessel stroke disease, and Ní Chróinín et al. [308]. As further proof of it, patients discontinuing statins even for short periods have been described to have a worse outcome and increased mortality [309].

The systematic review by Squizzato and co-workers concluded that data from RCTs were insufficient to consider statins as safe and effective drugs for IS [310]. A more recent systematic review by Hong et al. dealt with different aspects of statin treatment in IS [311]. Although no evidence of a benefit from statin treatment globally emerged from RCTs, the available data provided some interesting elements. First of all, pre-stroke statin therapy was confirmed to be beneficial in terms of reduced ischemic brain damage at the onset, functional outcome, and short-term mortality. Secondly, immediate post-stroke statin treatment could reduce functional disability and short-term mortality and supported the worsening of the outcome in case of withdrawal; the effect on short-term mortality could be related to the prevention of recurrent ischemic brain events. Thirdly, patients treated with thrombolysis gained a better outcome if treated with statins in spite of a certain increase in hemorrhagic complications. For this reason, some concerns have been raised by neurologists for statin use in IS patients undergoing thrombolysis [312]. The meta-analysis by Hong et al. [311] showed an increased risk for hemorrhagic transformation, as shown before, but also confirmed an improvement of the functional outcome with statin therapy, in contrast with previous meta-analyses [308,313,314], concluding that statins are not to be considered a contraindication for thrombolysis [311].

### 6.3. Fingolimod (FTY720)

Oral fingolimod (FTY720) is currently under investigation in a phase II randomized, open-label study including patients within 72 h of IS or spontaneous intracranial hemorrhage (available at: https://clinicaltrials.gov/ct2/show/NCT02002390). Primary outcome measures were clinical improvement up to 90 days, while secondary outcome measures were change in image and in immunology function up to 90 days. Considering the side effects of fingolimod, such as infections and cardiac rhythm alterations, the translational use of this drug could be limited in IS patients [315].

### 6.4. Donepezil

Exploiting the promising effects of donepezil in pre-clinical studies, the Mayo Acute Stroke Trial for Enhancing Recovery was designed to test the safety and tolerability of donepezil within 24 h from IS [280]. Donepezil was demonstrated to be safe and well tolerated with no treatment-related serious adverse events registered. Moreover, donepezil was shown to lead to cognitive improvements maybe through the increase in basal forebrain cholinergic systems, but these results are needed to be considered with caution in absence of a placebo-controlled comparison group. Donepezil was successfully tested as therapeutic choice for the treatment of Wernicke aphasia following a recent infarction in the right middle cerebral artery in a 53-year woman [316]. Donepezil has been proven effective in the re-organization of the cognitive neural network in patients with post-stroke cognitive impairment using functional MRI by increasing the activation of pre-frontal areas, inferior frontal lobes, and the left inferior parietal lobe [317].

### 6.5. Citalopram

TALOS is an ongoing, multicentre, randomized- and placebo-controlled, double-blind trial of citalopram in patients with IS comparing citalopram 10–40 mg daily and placebo within 7 days of first IS over a 6-month follow-up period (available at: https://clinicaltrials.gov/ct2/show/NCT01937182) [318]. The primary end point includes a composite outcome with vascular death, transient ischemic attack/stroke, and myocardial infarction and a functional outcome at 6 months evaluated by the modified Rankin scale. The secondary end point includes vascular mortality, all-cause death, stroke, bleeding, cognitive and organic cerebral impairment, post-stroke depression, pathological crying, and lesion size. No result is yet available, even if the restriction to first-ever stroke and the exclusion of patients with dementia could reduce the translational impact of this work.

### 6.6. Natalizumab (Anti-CD49d Antibody)

The ACTION trial (available at: https://clinicaltrials.gov/ct2/show/NCT01955707) is a multicenter, double-blind, placebo- and randomized-controlled study to evaluate the safety and efficacy of intravenous natalizumab on the reduction of infarct volume in IS. The final results were recently disclosed at the International Stroke Conference in Los Angeles in February 2016 showing the safety of this therapy. In IS patients, a single dose of natalizumab (300 mg intravenously) between 0 and 9 h did not change infarct volume from baseline to day 5 on MRI, but surprisingly functional outcomes measured by modified Rankin scale and Barthel index improved at day 90 [319].

### 6.7. Cyclosporine A

The Neuroprotection Impact of Cyclosporine A in Cerebral Infarction (available at: https://clinicaltrials.gov/ct2/show/NCT01527240) is a phase II study testing a single dose of cyclosporine A intravenously versus placebo on MRI infarct volume at 30 days in thrombolysed patients within 4.5 h from stroke symptom onset; the trial was completed but no result is still available. In a phase II, pilot study, cyclosporine A intravenously (2.0 mg/kg) combined with thrombolysis was evaluated in 127 IS patients. The primary end point was infarct volume on MRI at 30 days, whilst secondary end points were infarct volume according to the site of occlusion and recanalization after thrombolysis. Cyclosporin A demonstrated to be safe but failed to reduce the final infarct size; only in the subgroup of patients with proximal occlusion and successful recanalization, cyclosporine A did reduce infarct volume [289].

### 6.8. Edaravone (MCI-186)

Currently, MCI-186 is the one neuroprotectant candidate approved for the treatment of IS patients in Japan, but its use is restricted as grade B recommendation by Japanese guidelines [320]. This recommendation was based on a small Japanese RCT including edaravone 30 mg twice daily within a 72 h window [282]. Edaravone 30 mg twice daily was compared to ozagrel and showed non-inferiority in terms of functional outcomes in a multicenter, phase 4 randomized, open-label trial [321]. Edaravone was tested in an European double-blind, placebo-controlled RCT using two dosing regimens (loading dose 0.08 mg/kg + 0.2 mg/kg/h infusion or loading dose 0.16 mg/kg + 0.4 mg/kg/h infusion). No concerns on safety and tolerability were raised and therapeutic range was reached consistently during the infusion period [284]. Edaravone was found to abrogate MMP-9 levels in patients with IS [322,323]. As previously found in experimental models [274], in diabetic patients edaravone showed to significantly decrease NIHSS and Barthel index; moreover, the incidence of hemorrhage transformation, pulmonary infection, recurrent stroke, and seizures was markedly reduced [324]. In light of pre-clinical evidence [248,275], the combined therapy with recombinant t-PA and edaravone improved the recanalization rate, reduced the intracranial hemorrhage incidence, and ameliorated functional prognosis [295].

## 7. Conclusions

IS remains a great burden in modern society. Despite great clinical progresses having been made in recent years in order to improve diagnosis and treatment, beneficial long-term interventions, especially with regard to recovery, are still not available. We now know that the abrupt, dramatic inflammatory response immediately following IS can be evaluated as central or peripheral based on the brain or peripheral tissue origin. In recent years, our knowledge about macrophages has grown considerably as we now can consider M1 and M2 macrophage responses in the post-ischemic period. The spleen has gained great attention as a reservoir of inflammatory cells and cytokines released in the bloodstream following IS. Apart from widely known cytokines, chemokines, DAMPs, and autoantibodies as well as OPG and OPN represent important inflammatory mediators in the ischemic milieu and need to be considered in a therapeutic perspective. In addition to old therapies, such as statins, new treatments are being introduced, such as monoclonal antibodies, fingolimod, cyclosporine A, and edavarone showing promising results in pre-clinical studies and partly in phase 2 and 3 studies; nevertheless, much more efforts are needed. Notably, even if still under investigation, cell-based therapies may potentially become useful for other neurological diseases other than stroke. Bone marrow, human umbilical cord blood, and neural cells as well as adipose tissue-derived stromal cells could be therapeutic targets for the future, exploiting common mechanisms, such as paracrine release and immunomodulation [325]. We also need to increase our knowledge about currently known mediators, such as cytokines, given that the current approach is based on blocking of pro-inflammatory cytokines or supplement of anti-inflammatory cytokines [326]. Further trials are suggested in order to obtain more information on cytokine profile in order to appropriately select therapy according to each patient. Finally, inflammation is also needed for appropriate post-stroke recovery. Therefore, the potential risk of abrogating inflammation might per se raise some concerns on the safety of this therapeutic strategy. Future clinical studies investigating these aspects are welcomed.

## Figures and Tables

**Table 1:** Brain-specific inflammatory biomarkers potentially useful in stroke diagnosis, response to treatment, and outcome.

Author	Year	Study Design	Biomarker	Outcome	Results
Astroglial activation					
Dassan et al. [146]	2009	Systematic review (13 longitudinal studies)	S100B	IS diagnosis HT mRS	S100B may be useful in predicting clot lysis (*p* = 0.001) and HT after thrombolysis (*p* = 0.017) with sensitivity and specificity of 46% and 82%, respectively. S100B also predict final infarct volume and eventually functional outcome (sensitivity 87%, specificity 78%).
Ye et al. [147]	2015	Meta-analysis (10 pooled case-control studies enrolling 773 patients with IS and 438 healthy controls)	S100B	IS diagnosis	Serum levels of S100B were higher in IS patients as compared to controls (SMD = 1.71 [95% CI 0.62–2.79]; *p* = 0.002). Subgroup analysis based on ethnicity revealed that S100B predict IS progression in Asians but not in Caucasians. However, no statistical significance was observed in large samples.
Kazmierski et al. [148]	2015	Prospective observational (458 IS patients)	S100B	HT	HT was associated with higher serum concentrations of S100B (AUC = 0.746; sensitivity 92.9%, specificity 48.1%).
Tsai et al. [149]	2014	Case-control (100 IS patients and 80 healthy subject)	TBARS thiol	3-month NIHSS	As compared to controls, IS patients had higher TBARS and low free thiol. Furthermore, serum levels of thiol were lower in large- than small-vessel disease. TBARS at day 7 was identified as independent predictor of poor neurological outcome (OR 1.37 [95% CI 1.14–1.65]; *p* = 0.001).
Lorente et al. [150]	2015	Case-control (50 IS patients and 100 healthy controls)	MDA	30-day mortality	MDA levels were significantly higher in IS patients as compared to healthy controls, as well as in non-surviving IS patients than in survivors (*p* < 0.001 for both). Furthermore, MDA predicted 30-day mortality (OR 7.23 [95% CI 1.84–28.73]; *p* = 0.005) with a sensitivity of 65% and a specificity of 75% (AUC of 0.77).
Neuronal cell injury					
Bharosay et al. [151]	2012	Case-control (150 IS patients and 101 controls)	NSE	NIHSS at days 1–7	NSE was higher in IS patients (*p* < 0.001), also correlating with stroke severity at admission (*r* = 0.919; *p* < 0.001) and after 7 days (*r* = 0.706; *p* < 0.001).
Singh et al. [152]	2013	Case-control (100 IS patients and 101 controls)	NSE	NIHSS at admission	Serum NSE was higher in IS group, also correlating with IS severity (*r* = 0.800; *p* < 0.001).
Zaheer et al. [153]	2013	Prospective observational (75 IS patients)	NSE	30-day mRS	A positive correlation was found between NSE infarct size (*r* = 0.955, *p* < 0.001), whereas a negative relationship with GCS was demonstrated (*r* = −0.806, *p* < 0.001). Finally, there was a positive correlation between NSE and neurological outcome (*r* = 0.744, *p* < 0.001).
Kim et al. [155]	2014	Prospective observational (83 IS patients)	NSE	HT	In patients with HT, NSE time course was characterized by two peak levels. This specific pattern was significantly associated with the occurrence of HT (OR 6.84 [95% CI 1.12–41.70]; *p* = 0.04).
Lu et al. [156]	2015	Prospective observational (74 IS patients)	NSE	3-month mRS	NSE sowed predictive accuracy toward poor neurological outcome (77.1% sensitivity and 59.4% specificity). However, the adjusted RR for NSE was not effective in predicting poor neurological outcome.
Haupt et al. [157]	2016	Prospective observational (31 IS patients)	NSE	mRS days 7 and 10	NSE peak at day 4 in the good outcome patients, whereas a continuous increase was observed in those with poor outcome. Sensitivity of NSE analysis showing an increase over time to >90% at day 4.
Park et al. [154]	2013	Case-control (111 IS patients and 127 controls)	H-FABP	Stroke diagnosis	H-FABP was significantly higher in the IS group (OR 1.08 [95% CI 1.02–1.13]; *p* < 0.001). However, H-FABP was not sensitive enough to discriminate stroke from control group or IS subtype.

HT: hemorrhagic transformation; IS: ischemic stroke; SMD: standardized mean differences; CI: confidence interval; AUC: area under the curve; TBARS: thiobarbituric acid-reactive substances; OR: odds ratio; MDA: malonildyaldeide; NSE: neuron-specific enolase; NIHSS: National Institute of Health Stroke Scale; mRS: modified Rankin Scale; H-FABP: heart-type fatty acid binding protein.

**Table 2:** Non-specific inflammatory biomarkers potentially useful in stroke diagnosis, response to treatment, and outcome.

Author	Year	Study Design	Biomarker	Outcome	Results
Ozkan et al. [158]	2013	Prospective observational (62 IS patients)	CRP	Stroke subtype 3 months NIHSS	CRP was unable to predict IS subtype and functional disability at 3 months after IS.
Taheraghdam et al. [159]	2013	Prospective observational (102 IS patients)	CRP	3 months mRS	Early CRP measurement failed to predict IS outcome.
VanGilder et al. [160]	2014	Systematic review (5 longitudinal/case-control studies)	CRP	3 months mRS	In all studies, acutely elevated CRP was positively associated with long-term (30 days to 3 months) unfavorable outcome (OR ranging from 2.3 to 3.5; *p* < 0.05).
Karlinski et al. [161]	2014	Prospective observational (301 IS patients undergoing thrombolysis)	CRP	HT 3 months mRS	CRP measurement failed to independently predict the outcome of IS patients treated with thrombolysis.
Pandey et al. [162]	2014	Case control (880 IS patients, 32 HS and 50 healthy controls)	CRP	Day 7 NIHSS	CRP was significantly higher in stroke patients as compared to controls (*p* < 0.001 for both). When categorized on the basis of NIHSS, high serum levels of CRP were found in severe stroke group (*p* < 0.001 for both).
Li et al. [163]	2015	Prospective observational (374 IS patients)	PCT CRP	1-year mortality	Serum PCT levels were higher in non-survival patients (*p* < 0.001). Long-term mortality was independently predicted by both PCT (OR 3.64 [95% CI 1.54–5.88]) and CRP (OR 12.33 [95% CI 2.44–37.66]). As compared to CRP, PCT was a better predictor of mortality with a sensitivity of 81.5% and a specificity of 84.7% (AUC 0.887).
Rocco et al. [164]	2015	Prospective observational (1242 IS patients)	CRP	3 months mRS	Follow-up CRP, assessed during the first 7 days showed significant predictive value toward worse mRS (OR 2.67 [95% CI 1.76–4.06]) and mortality (OR 2.53 [95% CI 1.50–4.25]), with a c-statistic of 0.71 and 0.70, respectively.
Deng et al. [165]	2015	Case control (378 IS patients and 200 controls)	PCT	3-month mRS	Serum PCT was higher in IS group and correlated with lesion size and NIHSS (*p* < 0.001 for all). PCT predict worse functional outcome (OR 3.45 [95% CI 2.29–4.77]; *p* < 0.001) with a sensitivity of 75.4% and a specificity of 80.7% (AUC 0.845).
Wang et al. [166]	2016	Case-control (376 IS patients and 200 controls)	PCT CRP	1-year mRS 1-year mortality	Serum PCT was higher in patients with IS, and correlated with lesion size (*p* < 0.001). Both PCT and CRP correlated with and NIHSS (*p* < 0.001 for both). PCT predict worse functional outcome (OR 4.31 [95% CI 1.58–9.12]; *p* < 0.001). Mortality was independently predicted by both PCT (OR 1.10 [95% CI 1.05–1.15]) and CRP (OR 1.31 [95% CI 1.03–1.74]).
Matsuo et al. [167]	2016	Prospective observational (3653 IS patients)	CRP	3 months mRS	At multivariate analysis, CRP was associated with a poor outcome (OR 2.03 [95% CI 1.55–2.67]).
Geng et al. [168]	2016	Prospective observational (301 IS patients)	CRP	Discharge mRS Recurrent IS	At multivariate analysis, poor outcome at discharge was independently predicted by CRP (OR 4.89 [95% CI 3.06–7.81]).
Whiteley et al. [169]	2009	Systematic review (4 longitudinal studies)	IL-6	3-month mRS	Il-6 was identified as independent predictor of poor neurological outcome after IS (OR 1.05 [1.01–1.09]).
Park et al. [170]	2013	Prospective observational (175 IS patients)	IL-6	3-month mRS	In multivariate analysis IL-6 was independently associated with poor outcome (OR 1.75 [1.25–2.25]; *p* = 0.001).
Bustamante et al. [171]	2014	Meta-analysis (24 pooled longitudinal studies enrolling 4523 patients)	IL-6	1 to 6 months mRS	The highest quartile of IL-6 was an independent predictor of poor outcome (OR 2.35 [1.81–3.03], *p* < 0.001), but its additional predictive value was modest in terms of AUC (0.840 to 0.847).
Pusch et al. [172]	2015	Case-control study (76 patients with IS, 44 with carotid stenosis and 66 with Parkinson disease)	IL-6	Post-IS infection	High concentration of IL-6, MCP-1, and S100B at 6 h, and increase of P-selectin during the first 72 h were associated with post-stroke infections. Specifically, IL-6 predict the occurrence of post-stroke infection with an AUC of 0.920.
Lehmann et al. [173]	2015	Case-control study (95 patients with IS and 96 controls)	IL-6 CRP MMP-9	Stroke subtype	As compared to controls, LAAS, LAC and CEI had higher serum levels of IL-6, CRP, and MMP-9 (*p* < 0.05 for all).
Worthmann et al. [174]	2015	Prospective observational (56 IS patients)	IL-6 IL-10 CRP	Post-IS infection	IL-10, IL-6 and CRP show a different time course in patients with and without post-stroke infection. Furthermore, post-stroke infection is independently predicted by serum IL-10 (AUC 0.76) and CRP (AUC 0.74).
Fahmi et al. [175]	2016	Case-control (50 IS patients and 20 healthy controls)	IL-6	15-day NIHSS	At multivariate regression analysis, IL-6 was identified as independent predictor of short-tern neurological outcome (β = 0.451; *p* < 0.001).
Rodríguez-Yáñez et al. [176]	2013	Prospective observational (184 thrombolysed IS patients)	IL-10	3-month mRS	High levels of IL-10 predicted good functional outcome with a specificity of 88% and a sensibility of 86% (OR 2.86 [1.06–7.82]).
Ashour et al. [177]	2016	Case-control (60 IS patients and 30 healthy control)	IL-10	Post-IS infection	The occurrence of infectious was independently predicted by increased levels of IL-10 (OR 6.01 [1.53–23.51]; *p* = 0.01).
Inzitari et al. [179]	2013	Prospective observational (327 thrombolysed IS patients)	MMP-9	HT 3-months death 3-month mRS	Overtime MMP-9 variations (during 24 h across thrombolysis) significantly predicted HT (OR 1.40 [1.02–1.92]) and death (OR 1.58 [1.11–2.26]).
Carbone et al. [178]	2015	Case-control (60 thrombolysed IS patients and 30 not)	MMP-9	HT	Peak of MMP-9 (and also MMP-8 and MPO) at day 1 in thrombolysed patients was associated with increased rate of early HT (*p* = 0.023).

IS: ischemic stroke; NIHSS: National Institute of Health Stroke Scale; CRP: C-reactive protein; mRS: modified Rankin Scale; OR: odds ratio; HT: hemorrhagic transformation; PCT: procalcitonin; CI: confidence interval; AUC: area under the curve; IL: interleukin; MMP-9: matrix metalloproteinase; LAAS: large artery atherosclerotic stroke; LAC: lacunar stroke; CEI: cardio-embolic stroke; MPO: myeloperoxidase.

**Table 3:** Vascular biomarkers potentially useful in stroke diagnosis, response to treatment, and outcome.

Author	Year	Study Design	Biomarker	Outcome	Results
Wiseman et al. [180]	2014	Meta-analysis (42 pooled case-control studies enrolling 2196 lacunar IS and 2500 healthy controls)	t-PA, PAI-1, vWF d-dimer E-, P-selectins ICAM-1 VCAM-1	Stroke subtype	As compared to other subtypes, lacunar IS was characterized by higher markers of coagulation/fibrinolysis (t-PA, PAI-1, and d-dimer) and lower marker of endothelial dysfunction (vWF, E- and P-selectins, ICAM-1 and VCAM-1).
Liu et al. [181]	2016	Case-control (317 IS of different subtypes)	d-dimer	Stroke subtype	d-dimer was different in each group with the highest levels in the CE group (<0.001). d-dimer also independently predicted CE (OR 6.83 [95% CI 2.96–15.77]).
Yuan et al. [182]	2014	Prospective observational (300 IS patients)	d-dimer	Stroke subtype Day 14 NIHSS	Serum levels of d-dimer were higher in the CE group; Furthermore they correlated with neurological improvement (*r* = −0.410; *p* = 0.013).
Yang et al. [183]	2014	Prospective observational (220 IS patients)	d-dimer	3-month mRS 3-month mortality	Admission d-dimer was higher in patients with poor prognosis also in adjusted analysis (OR 2.18 [95% CI 1.55–2.83]) with high prognostic accuracy (AUC: 0.830). d-dimer also predicted mortality analysis (OR 3.22 [95% CI 2.05–6.43]).
Richard et al. [184]	2015	Prospective observational (100 IS patients)	E-, P-selectin ICAM-1 VCAM-1	3-month mRS	Early after SI, E-selectin was found to be an independent predictor of poor outcome (OR = 24.95 [95% CI 2–354]; *p* = 0.022 and AUC = 0.780), as was VCAM-1 during the third week after onset (OR = 8 [95% CI 2–37]; *p* = 0.01 and AUC 0.730).
Wang et al. [185]	2016	Case-control (1173 IS patients)	d-dimer	30-day mRS	d-dimer was effective in predicting poor neurological score (OR = 1.60 [95% CI 1.36–1.89]; *p* < 0.001).
Hsu et al. [186]	2016	Retrospective observational (307 thrombolysed IS patients)	d-dimer	3-month mRS HT	At adjusted analysis, higher levels of d-dimer at admission predicted poor outcome (OR = 1.90 [95% CI 1.27–2.86]; *p* = 0.002) and HT (OR = 2.97 [95% CI 1.15–7.70]; *p* = 0.025).

t-PA: tissue-type plasminogen activator; PAI: plasminogen activator inhibitor; vWF: Von Willebrand factor; ICAM: intracellular adhesion molecule; VCAM: vascular cell adhesion molecule; CE: cardio-embolic; OR: odds ratio; CI: confidence interval; IS: ischemic stroke; NIHSS: National Institute of Health Stroke Scale; mRS: modified Rankin Scale; AUC: area under the curve; ADMA: asymmetric dimethylarginine; HT: hemorrhagic transformation.

**Table 4:** New candidate inflammatory biomarkers potentially useful in stroke diagnosis, response to treatment, and outcome.

Author	Year	Study Design	Biomarker	Outcome	Results
Schulze et al. [201]	2013	Prospective observational (114 IS patients)	HMGB1	3-month mRS	Plasma HMGB1 weakly correlated with infarct volume and stroke severity at day 3 after IS. However, HMGB1 failed to independently predict long-term outcome.
Huang et al. [202]	2013	Prospective observational (338 IS patients)	HMGB1	1-year mRS	HMGB1 was independently associate with worse clinical outcome (OR 2.21 [95% CI 1.13–4.20]; *p* = 0.002) with 71.4% sensitivity and 83.7% specificity (AUC, 0.83). Furthermore, in a combined model, HMGB1 significantly improved the AUC of NIHSS score to 0.929 (*p* < 0.001).
Sapojnikova et al. [203]	2014	Case-control (42 IS patients and 32 healthy controls)	HMGB1	GOS	The increased HMGB1 levels and plasma MMP-9 are associated with a poor functional outcome and significantly correlated with each other (*p* < 0.05).
Marousi et al. [204]	2010	Prospective observational (82 IS patients)	Adiponectin	mRS at 1 and 6 months	Higher Adiponectin was indicative worse outcome on month 1 (OR = 1.14 [95% CI 1.01–1.29]; *p* = 0.031). However, adiponectin failed to predict IS severity, infarct size, recurrent IS, mortality, state, disability or functional outcome at 6 months.
Kuwashiro et al. [205]	2013	Case-control (171 IS patients and 171 healthy controls)	Adiponectin	3-month mRS	As compared to controls, average adiponectin values at onset were significantly lower and higher in patients with ATBI (*p* = 0.047) and CE (*p* = 0.008) IS, respectively. At onset adiponectin correlated with NIHSS (*r* = 0.420, *p* = 0.003) and was higher in patients with worse long-term outcome (*p* = 0.007).
Carbone et al. [132]	2015	Prospective observational (35 non-obese ATBI patients)	Adiponectin Leptin	3-month mRS	Serum leptin and leptin/adiponectin ratio at day 1 inversely correlated with both radiological and clinical parameters. Leptin/adiponectin ratio also independently predicted worse neurological outcome (OR = 0.15 [95% CI 0.03–0.83]; *p* = 0.030) and the occurrence of HT (OR = 0.08 [95% CI 0.01–0.81]; *p* = 0.028).

mRS: modified Rankin Scale; HMGB1: high-mobility group box-1; IS: ischemic stroke; OR: odds ratio; CI: confidence interval; AUC: area under the curve; NIHSS: National Institute of Health Stroke Scale; GOS: Glasgow outcome scale; MMP: matrix metalloproteinase; ATBI: atherothrombotic stroke; CE: cardio-embolic; HT: hemorrhagic transformation.

**Table 5:** Anti-inflammatory treatments in ischemic stroke: evidence from preclinical studies.

Study	Year	Treatment	Sample Size	Outcome
IL-1Ra				
Garcia et al. [256]	1995	13 with pMCAO and treated with IL-1Ra; 13 with pMCAO and treated with CSE buffer or placebo group (*n* = 13); 2 sham-operated animals treated with IL-1Ra or CSE (*n* = 2)	30 outbred male Wistar rats and fed Agway rat chow during the 4–6 quarantine days	IL-1Ra in rats with pMCAO significantly decreased the number of necrotic neurons both at 24 h and 7 days after the arterial occlusion (*p* < 0.0001). Neurological scores were also significantly improved with and a non-significant decrease in the number of PMN leukocytes in the ischemic hemisphere was observed.
Yamasaki et al. [247]	1995	60 min of tMCAO followed by reperfusion; first IL-1β, then anti-IL1β was injected	120 adult male Wistar rats	tMCAO induced an increase in brain water content, necrosis, and neutrophilic infiltration in the cortex perfused by the MCA and the DCP and VCP. rIL-1β into the left lateral ventricle immediately after reperfusion markedly enhanced ischemic brain edema formation and infarction size in MCA zone, DCP, and VCP in a dose-dependent manner (*p* < 0.01). Anti-IL-1β attenuated the post-ischemic increase of brain water content and decreased the infarction size (*p* < 0.01). The number of neutrophils infiltrating the ischemic area decreased with anti-IL-1β.
Relton et al. [236]	1996	MCAO or sham surgery. Animals were injected subcutaneously with either vehicle or rIL-1Ra at 0, 4, 8, 12, and 18 h after ischemia. In separate experiments, initial treatment was delayed until 30 min, 1 h, or 4 h after ischemia and treatments were repeated until 18 h	Male Sprague-Dawley rats	rIL-1Ra significantly inhibited infarct size by 46% at 24 h (*p* < 0.05), cerebral edema formation by 49% at 24 h (*p* < 0.05). Infarction inhibition by rIL-1Ra was dependent on dose and time of administration.
Pradillo et al. [234]	2012	Lean and Cp rats received placebo or IL-1Ra (25 and 12.5 mg/kg) subcutaneously at reperfusion and 6 h later and allocated to different groups: lean + tMCAO + placebo; lean + tMCAO + IL-1Ra; Cp + tMCAO + placebo; and Cp + tMCAO + IL-1Ra. For the delayed administration study, animals were injected subcutaneously with placebo or IL-1Ra at 3 h of reperfusion and again 3 h later	Male, lean and Cp rats	IL-1Ra at reperfusion resulted in a 50% reduction of infarct volume as measured by MRI both in lean and Cp compared with placebo-treated animals (*p* < 0.05). IL-1Ra decreased the number of MMP-9-positive neutrophils when compared with placebo (*p* < 0.05). In both lean and Cp rats, IL-1Ra largely reduced the microglial activation compared with the placebo-treated groups (*p* < 0.05). In 16-month-old lean rats, delayed IL-1Ra significantly reduced the number of MMP-9-positive blood vessels and the number of MMP-9-positive neutrophils when compared with the placebo group (*p* < 0.05).
Statins				
Endres et al. [219]	1998	After tMCAO followed by reperfusion, mice were injected subcutaneously with 0.1 mL of activated simvastatin or lovastatin (0.2–20 mg/kg) or a corresponding volume of PBS once daily for 3 or 14 days	Not declared	In a concentration-dependent manner, simvastatin for 14 days reduced cerebral infarct size by 18, 27 and 46% (*p* < 0.05) and increased NOS activity (*p* < 0.05). Simvastatin 20 mg/kg increased basal hemispheric CBF by 31% (*p* < 0.05). Lovastatin 20 mg/kg daily for 14 days also decreased cerebral infarct size and neurological deficits, even if to a lesser extent than simvastatin.
Kawashima et al. [222]	2003	Two groups, one statin-treated (cerivastatin 2 mg/kg by gavage once daily) and another vehicle-treated	Stroke-prone spontaneously hypertensive rats (4 weeks of age)	The incidence of stroke and stroke size decreased (*p* < 0.01). High-dose statin treatment delayed early death and reduced the occurrence of stroke-associated symptoms (*p* < 0.01) and decreased stroke-associated infiltration of inflammatory cells (*p* < 0.05). Statin treatment increased eNOS protein levels and eNOS activity (*p* < 0.05). Superoxide production was reduced in statin-treated rats (*p* < 0.01).
Amin-Hanjani et al. [211]	2001	Two groups: mevastatin at a dose of 2 or 20 mg/kg daily and a corresponding concentration of vehicle for 7, 14, or 28 days before tMCAO	Wild-type male mice and eNOS-deficient male mice	Mevastatin increased levels of eNOS mRNA and protein, reduced infarct size, and improved neurological deficits in a dose- and time-dependent manner especially with 14- and 28-day high-dose treatment (26% and 37% infarct reduction, respectively, *p* < 0.05). Cholesterol levels were reduced only after 28 days of treatment (*p* < 0.05), but did not correlate with infarct reduction. Baseline absolute cerebral blood flow was 30% higher after 14-day high-dose treatment (*p* < 0.05).
Prinz et al. [235]	2008	After tMCAO followed by reperfusion, mice were treated with intravenously or intraperitoneally rosuvastatin given up to 6 h after MCAO (0.02–20 mg/kg)	Wild-type mice aged 6 to 8 weeks	Intravenous rosuvastatin significantly reduced lesion size up to 4 h after MCAO in doses as low as 0.2 mg/kg (*p* < 0.05). Intraperitoneal administration provided protection only on reperfusion at a dose of 20 mg/kg (*p* < 0.05). Lesion protection was evident 5 days after brain ischemia and was associated with functional improvements at 2.0 mg/kg dose (*p* < 0.05). Neuroprotection with intravenous rosuvastatin was achieved with peak plasma concentrations <0.5 ng/mL and was associated with increased levels of phosphorylated Akt kinase and eNOS in the vasculature (*p* < 0.05).
Asahi et al. [212]	2005	Heterologous blood clots were used to induce MCAO after long-term simvastatin (20 mg/kg), atorvastatin (20 mg/kg) or vehicle treatment subcutaneously	Male SV-129 mice and male C57Bl/6 mice	In wild-type mice, both simvastatin and atorvastatin reduced ischemic lesions and residual clot after 14 days (*p* < 0.05). In eNOS knockout mice, atorvastatin reduced the volume of ischemic tissue and improved neurologic outcomes after arterial occlusion (*p* < 0.05). Both statins did not have protective effects in t-PA knockout mice after embolic focal ischemia, but only in a filament model where focal ischemia was achieved via mechanical occlusion (*p* < 0.05).
Chen et al. [215]	2003	24 h after MCAO, rats were fed atorvastatin (1, 3 or 8 mg/kg) daily for 7 days. Rats were also treated with simvastatin 1 mg/kg with the same protocol	48 Adult male Wistar rats	Rats treated with 1 and 3 mg/kg atorvastatin and 1 mg/kg simvastatin improved functional recovery (*p* < 0.05). VEGF production within the ischemic boundary area at 14 days after stroke increased in the 1 mg/kg atorvastatin group (*p* < 0.05) as well as cyclic guanosine monophosphate, angiogenesis, neurogenesis, and synaptophysin levels (*p* < 0.05).
Sironi et al. [242]	2003	Two groups of rats were treated with vehicle alone or simvastatin for 3 days before MCAO, while other two groups underwent MCAO and were treated with vehicle or simvastatin at 3 and 25 h after the induction of the injury. The brain infarct size was evaluated using MRI	Male Sprague-Dawley rats	Treatment with simvastatin (20 mg/kg) after MCAO prevented the increase in brain infarct volume occurring at 24 h and induced a 46.6% reduction after 48 h (*p* < 0.01). The neuroprotective effects of simvastatin were paralleled by an increase in eNOS immunoreactivity, detectable in the brain of simvastatin-treated rats.
Reuter et al. [238]	2015	Cultured hBMECs pretreated with simvastatin and subjected to OGD	hBMECs	Simvastatin significantly blocked the expression of MMP-2 under OGD (*p* < 0.004). MMP-9 synthesis rate was low and unaffected by simvastatin treatment, while the gene expression and protein secretion of TIMP-1 and TIMP-2 were both strongly induced (*p* < 0.001).
Fingolimod (FTY720)				
Rolland et al. [239]	2013	Fingolimod was given intraperitoneally at a dose of 1 mg/kg as single dose 1 h after ICH induction or daily administration 1, 24, and 48 h after ICH induction	103 male CD-1 mice and 28 male Sprague-Dawley rats	Fingolimod enhanced neurological functions and reduced brain edema at 24 and 72 h following experimental ICH in CD-1 mice (*p* < 0.05). Fewer lymphocytes were found in blood and brain samples of treated animals (*p* < 0.05). Fingolimod decreased ICAM-1, IFN-γ, IL-17 levels 72 h after ICH (*p* < 0.05). Treated Sprague-Dawley rats showed less spatial and motor learning deficits along with significantly reduced brain atrophy and neuronal cell loss within the basal ganglia (*p* < 0.05).
Campos et al. [214]	2013	3 cohorts: pMCAO not treated with t-PA; tMCAO followed by early (30 min after thrombin) t-PA administration; and tMCAO followed by delayed (3 h after thrombin) t-PA administration. Each of these cohort received fingolimod at different time points	C57BL/6 male mice	Fingolimod reduced neurological deficits and infarct volume after in situ thromboembolic MCAO (*p* < 0.05). Combination of fingolimod and t-PA improved neurological outcomes of the thrombolytic therapy and the risk of hemorrhagic transformation associated with delayed administration of t-PA (*p* < 0.05).
Donepezil				
Wang et al. [244]	2014	3 groups: the sham operation group (SO), the model group (MG) and the treatment group (TG). Pathological appearance of the hippocampal CA1 region and calpain I and CDK5/p25 expression were observed on the 4th, 6th and 8th week from I/R surgery	250 3-month old male mice	At each postoperative time point, the normal neuron count of the hippocampal CA1 region in the treatment group increased significantly (*p* < 0.05), whereas calpain I and CDK5/p25 expression, SOD activity and MDA content were significantly lower than those in the model group (*p* < 0.05).
Min et al. [231]	2012	After transient global ischemia, donepezil (5 mg/kg once a day) was administered intragastrically for 21 days	Male Mongolian gerbils	Donepezil significantly inhibited delayed neuronal death in the hippocampal CA1 region (*p* < 0.01). Memory impairment was significantly improved by donepezil treatment (*p* < 0.05–0.01). Western blot analysis showed that donepezil treatment prevented reductions in p-CaMKII and p-CREB protein levels in the hippocampus (*p* < 0.01).
Yuan et al. [252]	2011	Cultured cells were exposed to both OGD and electrophysiological experiment	HEK293 cells from a human embryonic kidney cell line	Donepezil showed to attenuate OGD-induced apoptosis in Kv2.1/HEK293 cells and to inhibit Kv2.1 currents in a dose-dependent manner under normoxic condition (*p* < 0.01). Donepezil further inhibited Kv2.1 currents after OGD treatment (*p* < 0.05).
Akasofu et al. [210]	2008	Prolonged opening of sodium channels with veratridine led to depolarization-induced neuronal cell injury, which was prevented by 0.1 µM tetrodotoxin	Cortical cell cultures from fetal rats of the Wistar strain	Pre-treatment with donepezil (0.1–10 µM) for 1 day significantly decreased cell death and increased cell viability in a concentration-dependent manner (*p* < 0.05). At 0.1–10 µM, donepezil concentration-dependently decreased the veratridine-induced increase of calcium concentration, whilst at 10 µM it reduced the veratridine-induced increase of sodium concentration (*p* < 0.05 for both).
Lee et al. [225]	2007	After permanent ligation of bilateral common carotid arteries, rats were administered cilostazol (30 mg/kg/day orally) and donepezil (0.3 mg/kg/day intraperitoneally)	Rats	Concurrent treatment with cilostazol and donepezil prevented neuropathological alterations in the white matter by activation of phosphorylated CREB and Bcl-2, resulting in improvement of spatial learning memory (*p* < 0.05).
Citalopram				
Espinera et al. [220]	2013	After focal ischemic stroke, citalopram 10 mg/kg was injected intraperitoneally 24 h after stroke and then daily for 7, 14, 21, or 28 days	Adult male C57 mice	Citalopram had no significant effect on infarct formation or edema 3 days after stroke, but enhanced sensorimotor functional recovery after 14 days (*p* < 0.05). Citalopram improved neuroblast proliferation and migration (*p* < 0.01) as well as neurogenesis (*p* < 0.05) and peri-infarction vessel density (*p* < 0.05) in the post-ischemic brain.
Kronenberg et al. [223]	2012	Mice were subjected to 30-min MCAO/reperfusion and serial MRI scans; a subset of animals received citalopram from day 7 after MCAO	Male 129/SV mice	Delayed citalopram reversed the behavioral phenotype blocked the degeneration of dopaminergic midbrain neurons, and attenuated striatal atrophy after 4 months (*p* < 0.05).
Natalizumab				
Becker et al. [213]	2001	Rats underwent 3 h of MCAO followed by 45 h of reperfusion. 2 h after ischemia, one group received anti-α4 integrin antibody intraperitoneally and another an isotype control antibody	Male Lewis rats	Neurological deficits were less frequent in treated rats at 24 (*p* < 0.01) and 48 h (*p* = 0.01) after ischemia. White blood cell count was higher in treated rats (*p* < 0.01) with a lymphocyte/monocyte predominance. Infarction volume was reduced in treated animals (*p* = 0.012).
Relton et al. [237]	2001	Rats underwent 1-h MCAO followed by 23-h reperfusion. 24 h before MCAO were injected intravenously with anti-α4 integrin antibody (2.5 mg/kg) or isotype control antibody	Male spontaneously hypertensive rats or Sprague-Dawley rats	Treated animals showed reduced total infarct volume (*p* < 0.05–0.01). Moreover, treatment reduced brain myeloperoxidase activity (*p* < 0.05). No significant difference in white blood cell count was observed. Leukocyte counts were elevated in TA-2-treated rats.
Liesz et al. [226]	2011	24 h before or 3 h after ischemia, mice were administered 300 mg of CD49d-specific monoclonal antibody intraperitoneally after; control animals received rat IgG2b isotype control monoclonal antibody	Male mice C57BL/6J aged 10–12 weeks	VLA-4 blockade improved outcome after 7 days from MCAO via the inhibition of cerebral leukocyte invasion and neurotoxic cytokine production (*p* < 0.01). VLA-4 inhibition reduced the post-ischemic VCAM-1 up-regulation (*p* < 0.01).
Langhauser et al. [224]	2014	24 h before or 3 h after cerebral ischemia (both tMCAO and pMCAO), mice were treated with 300 μg of a monoclonal antibody anti-CD49d	Male C57Bl/6 mice	VLA-4 blocking reduced T cell and neutrophil invasion after 5 days following MCAO and inhibited the up-regulation of VCAM-1 (*p* < 0.05). Anti-CD49d antibody could not influence stroke outcome positively, irrespective of the model or the time point investigated.
Neumann et al. [232]	2015	After focal cerebral ischemia was induced by pMCAO, anti-CD49d treatment was administered intravenously	LysM-eGFP mice	The systemic blockade of VLA-4 resulted in reduction of adherence of neutrophils (*p* < 0.05) and inhibition of their infiltration (*p* < 0.01) 24 h after focal ischemia. Moreover, anti-VLA-4 treatment improved neurological outcome and reduced infarct volume at day 3 after stroke (*p* < 0.05).
Llovera et al. [230]	2015	After cMCAO (for small lesions confined to the cortex) or fMCAO (for lesions in the cortex and subcortical structures) was assessed, anti-CD49d treatment was administered intraperitoneally 3 h after stroke induction	315 male C57BL/6J mice	Anti-CD49d treatment reduced infarct volume (*p* < 0.05) and leukocytes invasion into the ischemic brain (*p* < 0.001) after 7 days from cMCAO (*p* < 0.05). After fMCAO, mice had fewer cerebral leukocytes than after cMCAO (*p* < 0.001), but anti-CD49d treatment did not affect leukocyte invasion after fMCAO.
Cyclosporine A				
Uchino et al. [243]	1998	CsA was given intraperitoneally daily for 1 week before and 1 week after forebrain ischemia of 7 or 10 min duration	Rats	Systemically administered CsA ameliorated the damage to the CA1 sector of the hippocampus due to transient ischemia (*p* < 0.001).
Cho et al. [217]	2013	Rats underwent MCAO and then randomly treated by intracarotid CsA 10 mg/kg 20 min before MCAO (pre-treatment group); intracarotid CsA 10 mg/kg immediately after reperfusion (post-treatment); and intracarotid saline immediately after reperfusion	27 Sprague-Dawley rats	On day 1, a significant reduction of infarct size in the pre-treatment group compared to the post-treatment (*p* < 0.004) was evaluated. A significant reduction of microglial cell count in the pre-treatment group compared to either saline or post-treatment groups was found (*p* < 0.001).
Yu et al. [250]	2004	Rats underwent MCAO then were randomly treated with either: low dose CsA, MP, low dose CsA plus MP, high dose CsA, or vehicle	Adult Sprague-Dawley rats	Animals receiving high dose CsA alone exhibited a minor motor asymmetry and less neurologic deficits 3 days after stroke (*p* < 0.0001) as well as those receiving low dose CsA and MP treatment but only on day 1 post-stroke (*p* <0.005). Animals receiving high dose CsA alone exhibited significantly (*p* < 0.0001).
Yuen et al. [253]	2011	Rats were equally divided into sham control, intraperitoneal physiological saline (at 0.5/24/48 h after stroke), CsA (20 mg/kg at 0.5/24 h intraperitoneally), EPO (5000 IU/kg at 0.5/24/48 h, subcutaneously), combined CsA and EPO after occlusion of distal left internal carotid artery	50 adult-male Sprague-Dawley rats	On day 21, improvement in neurological function was found in CsA and EPO group (*p* < 0.05) and was higher when the combined treatment was administered (*p* < 0.004). Attenuation of inflammatory response, apoptosis, and oxidative stress was found with combined therapy with CsA and EPO (*p* < 0.05).
Edaravone				
Fujiwara et al. [221]	2016	Before 90-min MCAO followed by reperfusion, rats were randomly assigned to intravenous vehicle or intravenous edaravone 3 mg/kg	Male Sprague-Dawley rats	Edaravone decreased infarct volume and edema formation and IL-1β and MMP-9 levels 3 h after ischemia levels (*p* < 0.05). Edaravone was shown to reduce levels of many other pro-inflammatory cytokines.
Yamashita et al. [248]	2015	Thrombolysis was evaluated by using a He-Ne-laser-induced thrombosis model in mesenteric microvessels. 3 experimental groups (placebo, alteplase 0.6 mg/kg, alteplase 0.6 mg/kg + edaravone 10.5 mg/kg)	Male Wistar–ST rats	In the alteplase group, thrombus volume decreased (*p* < 0.01) after 20 min. In the alteplase+edaravone group, thrombus volume was more evident (*p* < 0.001).
Wu et al. [245]	2014	Rats were subjected to tMCAO and then administered edaravone 2.4 mg/kg; a subset of these animals were administered both edaravone 2.4 mg/kg and borneol 0.6 mg/kg	Sprague-Dawley rats	Edaravone was demonstrated to scavenge free radicals. Edavarone and borneol reduced the infarct area (*p* < 0.001) and the effect was increased when drugs were administered synergistically (*p* < 0.001).

IL-1Ra: interleukin-1 receptor antagonist; pMCAO: permanent middle cerebral artery occlusion; CV: cardiovascular; PMN: polymorphonuclear; tMCAO: transient middle cerebral artery occlusion; DCP: dorsal area of caudate putamen; VCP: ventral area of the caudate putamen; rIL-1β: recombinant interleukin-1β; MRI: magnetic resonance imaging; MMP: metalloproteinase; CBF: cerebral blood flow; eNOS: endothelial nitric oxide synthase; t-PA: tissue-type plasminogen activator; VEGF: vascular endothelial growth factor; hBMEC: human brain microvascular endothelial cells; OGD: oxygen glucose deprivation; ICH: intracerebral hemorrhage; ICAM-1: intercellular adhesion molecule-1; IFN-γ: interferon-γ; IL: interleukin; I/R: ischemia/reperfusion; CDK5: cyclin-dependent kinase 5; SOD: superoxide dismutase; MDA: malondialdehyde; CaMKII: calmodulin-dependent protein kinase II; CREB: cyclic adenosine monophosphate responsive element binding protein; Kv channels: voltage-gated potassium channels; VCAM-1: vascular cell adhesion molecule-1; VLA-4: very late antigen-4; LysM–EGFP: lysozyme M promoter driving expression of enhanced green fluorescent protein; cMCAO: coagulation of the distal middle cerebral artery; fMCAO: occlusion of the middle cerebral artery with an endovascular filament; CsA: cyclosporine A; MP: methylprednisolone; EPO: erythropoietin.

**Table 6:** Randomized clinical trials in ischemic stroke.

Study	Year	Treatment	Sample Size	Outcome
IL-1Ra				
Emsley et al. [283]	2005	Within 6 h of the stroke onset, patients were randomized to rhIL-1ra (intravenously by a 100 mg loading dose over 60 s, followed by a 2 mg/kg/h infusion over 72 h.) or placebo.	34 patients (17 rhIL-1Ra, 17 placebo)	Peripheral total white blood cell and neutrophil count, CRP, and IL-6 and neutrophil counts were lower in the rhIL-1ra-treated were lower in the treated group. The drug was safe and well tolerated.
Smith et al. [294]	2012	Blood samples prior to treatment initiation, at 24 h and 5 to 7 days. LPS stimulation was made to assess cytokine production by leukocytes.	34 patients (17 rhIL-1Ra, 17 placebo)	Induction of TNF-α (*p* < 0.001), IL-1β (*p* < 0.005), IL-6, IL-8, and IL-10 (*p* < 0.02) by LPS was reduced in patients at admission. At 24 h, for patients treated with IL-1Ra, induction of TNF-α, IL-6 and IL-10 was greater than in the placebo group (*p* < 0.05). At 5 to 7 day, TNF-α and IL-1β induction remained suppressed only in the placebo group (p < 0.05). Plasma cortisol concentrations were elevated at admission in patients compared to controls but decreased at 24 h in treated patients (*p* < 0.05) and inversely correlated (*p* < 0.001) with either TNF-α or IL-1β induction at admission.
Statins				
Scandinavian Simvastatin Survival Study (4S) [277]	1994	Patients with angina pectoris or previous MI and serum cholesterol 5.5–8.0 mmol/L on a lipid-lowering diet were randomized to double-blind treatment with simvastatin or placebo.	4444 patients (2221 simvastatin, 2223 placebo)	Over 5.4 years, simvastatin improved lipid profile, with few adverse effects. The relative risk of death in the simvastatin group was 0.70 (95% CI 0.58–0.85, *p* = 0.0003). In a *post* hoc analysis, simvastatin was demonstrated to reduce by 30% the rate of strokes and transient ischemic attacks.
Plehn et al. [290]	1999	Enrolled patients: 21–75 years old who had experienced a myocardial infarction within the past 3 to 20 months, total cholesterol <240 mg/dL, LDL cholesterol between 115 and 174 mg/dL, and fasting triglycerides <350 mg/dL during 4 weeks of treatment.	4159 patients (2081 pravastatin 40 mg daily and 2078 placebo)	Compared with placebo, pravastatin lowered total and LDL cholesterol, and triglycerides by 20%, 32%, and 14%, respectively. A total of 128 strokes (52 on pravastatin, 76 on placebo) and 216 strokes or TIAs (92 on pravastatin, 124 on placebo) were observed, representing a 32% reduction (95% CI, 4%–52%, *p* = 0.03) in all-cause stroke and 27% reduction in stroke or TIA (95% CI, 4%–44%, *p* = 0.02). No increase in hemorrhagic stroke with pravastatin was found.
Montaner et al. [288]	2008	Simvastatin (40 mg/day for the first week followed by a dose of 20 mg/day until day 90) or placebo were given at 3–12 h from symptom onset.	60 patients (30 simvastatin, 30 placebo)	Simvastatin-treated group presented greater improvements at several time points (*p* = 0.01). Simvastatin treatment and low temperatures were the only independent predictors of a great improvement by day 90 (OR 10.3, CI 2.05–52.2, *p* = 0.005 and OR 0.13, CI 0.02–0.70, *p* = 0.017, respectively).
Sever et al. [292]	2003	Hypertensive patients aged 40–79 years with at least 3 other cardiovascular risk factors.	10305 (5168 atorvastatin 10 mg daily and 5137 placebo)	Treatment was stopped after a median follow-up of 3.3 years. In the atorvastatin group, less primary events occurred (HR 0.64, 95% CI 0.50–0.83, *p* = 0.0005), especially in the first year of follow-up. Fatal and non-fatal stroke (*p* = 0.024), total cardiovascular events and total coronary events (*p* = 0.0005) were also lowered.
Amarenco et al. [278]	2006	Patients with previous stroke or TIA within one to six months, LDL cholesterol levels of 100 to 190 mg/dL, and no known coronary heart disease.	4731 patients (2365 atorvastatin 80 mg daily and 2366 placebo)	During 4.9 years, 265 patients under atorvastatin and 311 under placebo had a fatal or non-fatal stroke (5-year absolute reduction in risk, 2.2%; adjusted HR 0.84, 95% CI, 0.71–0.99, *p* = 0.03; unadjusted *p* = 0.05). The 5-year absolute reduction in the risk of major cardiovascular events was 3.5% (HR, 0.80, 95% CI, 0.69–0.92, *p* = 0.002). No difference in mortality rate was seen.
Shepherd et al. [293]	2002	Patients aged 70–82 years with a history of or risk factors for vascular disease.	5804 patients (2891 pravastatin 40 mg daily and 2913 placebo)	Pravastatin lowered LDL cholesterol and reduced the incidence of the primary endpoint (HR 0.85, 95% CI 0.74–0.97, *p* = 0.014). Coronary heart disease death and non-fatal MI risk was also reduced (*p* = 0.006). Stroke risk was unaffected (*p* = 0.8) as well as for TIA (*p* = 0.051). New cancer diagnosis were more frequent in pravastatin group (*p* = 0.020). Mortality from coronary heart disease was lower in the pravastatin group (*p* = 0.043). No significant effect on cognitive function or disability was found.
Ridker et al. [291]	2008	Apparently healthy men and women with LDL cholesterol levels of less than 130 mg/dL and hs-CRP levels of 2.0 mg/L or higher.	17802 patients (8901 rosuvastatin 20 mg daily and 8901 placebo)	Rosuvastatin reduced LDL cholesterol levels and hs-CRP levels. Rates of occurrence of the combined primary end point (MI, stroke, arterial revascularization, hospitalization for unstable angina, or death from cardiovascular causes) were 0.77 for rosuvastatin (HR 0.56, 95% CI: 0.46–0.69, *p* < 0.00001; HR for stroke 0.52, 95% CI 0.34–0.79, *p* = 0.002).
Donepezil				
Barrett et al. [280]	2011	Adults with ischemic stroke treated within 24 h after onset of symptoms.	33 patients receiving donepezil 5 mg daily for 30 days followed by an increase to 10 mg/day for 60 days	15 participants had a favorable clinical outcome (NIHSS score ≤1 at day 90) (*p* < 0.001).
Cyclosporine A				
Nighoghossian et al. [289]	2015	Patients aged 18–85 years with an anterior-circulation stroke and eligible for thrombolytic therapy and evaluation of infarct volume on MRI at 30 days.	127 patients (61 CsA 2 mg/kg and 66 saline)	The reduction of infarct volume in CsA-treated patients was not significant (*p* = 0.18). In patients with proximal occlusion and effective recanalization, infarct volume decreased in CsA-treated group (*p* = 0.009).
Edaravone				
Edaravone Acute Infarction Study Group [282]	2003	Patients with acute ischemic stroke within 72 h from symptom onset.	250 patients (125 edaravone 30 mg twice a day for 14 days and 125 placebo)	A significant improvement in functional outcome evaluated by the mRS was observed in the edaravone group (*p* = 0.039).
Kaste et al. [284]	2013	Patients with acute ischemic stroke within 24 h from stroke onset.	36 patients (12 edaravone with loading dose 0.08 mg/kg + 0.2 mg/kg/h; 13 edaravone loading dose 0.16 mg/kg + 0.4 mg/kg/h; 11 placebo)	Both doses of the new formulation and dosing regimen were well tolerated and showed clinical improvement based on NIHSS score.
Takenaka et al. [295]	2014	Patients admitted to hospital for cerebral infarction within 3 h after the onset of infarction.	48 patients (20 edaravone before rt-PA and 28 edaravone and rt-PA simultaneously)	NIHSS before rt-PA showed a statistically significant improvement after rt-PA administration (*p* < 0.001). The mRS at 90 days also improved.

rhIL-1Ra: recombinant human IL-1 receptor antagonist. LPS: lipopolysaccharide. TNF-α: tumor necrosis factor-α. IL: interleukin. MI: myocardial infarction. LDL: low density lipoprotein. TIA: transient ischemic attack. CI: confidence interval. OR: odds ratio. HR: hazard ratio. Hs-CRP: high sensitivity C-reactive protein. NIHSS: National Institute of Health Stroke Scale. CsA: cyclosporine A. MRI: magnetic resonance imaging. mRS: modified Rankin scale. rt-PA: recombinant tissue-type plasminogen activator.

## References

[1] Warlow C., Sudlow C., Dennis M., Wardlaw J., Sandercock P. (2003). Stroke. Lancet.

[2] Lloyd-Jones D., Adams R.J., Brown T.M., Carnethon M., Dai S., de Simone G., Ferguson T.B., Ford E., Furie K., Writing Group Members (2010). Heart disease and stroke statistics—2010 Update: A report from the american heart association. Circulation.

[3] Jin R., Yang G., Li G. (2010). Inflammatory mechanisms in ischemic stroke: Role of inflammatory cells. J. Leukoc. Biol..

[4] Petrovic-Djergovic D., Goonewardena S.N., Pinsky D.J. (2016). Inflammatory disequilibrium in stroke. Circ. Res..

[5] Yellon D.M., Hausenloy D.J. (2007). Myocardial reperfusion injury. N. Engl. J. Med..

[6] Eltzschig H.K., Eckle T. (2011). Ischemia and reperfusion—From mechanism to translation. Nat. Med..

[7] Chen G.Y., Nunez G. (2010). Sterile inflammation: Sensing and reacting to damage. Nat. Rev. Immunol..

[8] Kim J., Song T.J., Park J.H., Lee H.S., Nam C.M., Nam H.S., Kim Y.D., Heo J.H. (2012). Different prognostic value of white blood cell subtypes in patients with acute cerebral infarction. Atherosclerosis.

[9] Tsai N.W., Chang W.N., Shaw C.F., Jan C.R., Lu C.H. (2010). Leucocyte apoptosis in patients with acute ischaemic stroke. Clin. Exp. Pharmacol. Physiol..

[10] Garcia J.H., Liu K.F., Bree M.P. (1996). Effects of CD11b/18 monoclonal antibody on rats with permanent middle cerebral artery occlusion. Am. J. Pathol..

[11] Lopes Pinheiro M.A., Kooij G., Mizee M.R., Kamermans A., Enzmann G., Lyck R., Schwaninger M., Engelhardt B., de Vries H.E. (2016). Immune cell trafficking across the barriers of the central nervous system in multiple sclerosis and stroke. Biochim. Biophys. Acta.

[12] Perez-de-Puig I., Miro-Mur F., Ferrer-Ferrer M., Gelpi E., Pedragosa J., Justicia C., Urra X., Chamorro A., Planas A.M. (2015). Neutrophil recruitment to the brain in mouse and human ischemic stroke. Acta Neuropathol..

[13] Enzmann G., Mysiorek C., Gorina R., Cheng Y.J., Ghavampour S., Hannocks M.J., Prinz V., Dirnagl U., Endres M., Prinz M. (2013). The neurovascular unit as a selective barrier to polymorphonuclear granulocyte (PMN) infiltration into the brain after ischemic injury. Acta Neuropathol..

[14] Taichman N.S., Young S., Cruchley A.T., Taylor P., Paleolog E. (1997). Human neutrophils secrete vascular endothelial growth factor. J. Leukoc. Biol..

[15] Christoffersson G., Vagesjo E., Vandooren J., Liden M., Massena S., Reinert R.B., Brissova M., Powers A.C., Opdenakker G., Phillipson M. (2012). VEGF-A recruits a proangiogenic MMP-9-delivering neutrophil subset that induces angiogenesis in transplanted hypoxic tissue. Blood.

[16] Cauwe B., Martens E., Proost P., Opdenakker G. (2009). Multidimensional degradomics identifies systemic autoantigens and intracellular matrix proteins as novel gelatinase B/MMP-9 substrates. Integr. Biol..

[17] Segel G.B., Halterman M.W., Lichtman M.A. (2011). The paradox of the neutrophil’s role in tissue injury. J. Leukoc. Biol..

[18] Akopov S.E., Simonian N.A., Grigorian G.S. (1996). Dynamics of polymorphonuclear leukocyte accumulation in acute cerebral infarction and their correlation with brain tissue damage. Stroke.

[19] Hernandez L.A., Grisham M.B., Twohig B., Arfors K.E., Harlan J.M., Granger D.N. (1987). Role of neutrophils in ischemia-reperfusion-induced microvascular injury. Am. J. Physiol..

[20] Rorvig S., Honore C., Larsson L.I., Ohlsson S., Pedersen C.C., Jacobsen L.C., Cowland J.B., Garred P., Borregaard N. (2009). Ficolin-1 is present in a highly mobilizable subset of human neutrophil granules and associates with the cell surface after stimulation with fmlp. J. Leukoc. Biol..

[21] Jickling G.C., Liu D., Ander B.P., Stamova B., Zhan X., Sharp F.R. (2015). Targeting neutrophils in ischemic stroke: Translational insights from experimental studies. J. Cereb. Blood Flow Metab..

[22] Davalos D., Grutzendler J., Yang G., Kim J.V., Zuo Y., Jung S., Littman D.R., Dustin M.L., Gan W.B. (2005). ATP mediates rapid microglial response to local brain injury in vivo. Nat. Neurosci..

[23] Prinz M., Priller J. (2014). Microglia and brain macrophages in the molecular age: From origin to neuropsychiatric disease. Nat. Rev. Neurosci..

[24] Liu Z., Fan Y., Won S.J., Neumann M., Hu D., Zhou L., Weinstein P.R., Liu J. (2007). Chronic treatment with minocycline preserves adult new neurons and reduces functional impairment after focal cerebral ischemia. Stroke.

[25] Hoehn B.D., Palmer T.D., Steinberg G.K. (2005). Neurogenesis in rats after focal cerebral ischemia is enhanced by indomethacin. Stroke.

[26] Kim B.J., Kim M.J., Park J.M., Lee S.H., Kim Y.J., Ryu S., Kim Y.H., Yoon B.W. (2009). Reduced neurogenesis after suppressed inflammation by minocycline in transient cerebral ischemia in rat. J. Neurol. Sci..

[27] Faustino J.V., Wang X., Johnson C.E., Klibanov A., Derugin N., Wendland M.F., Vexler Z.S. (2011). Microglial cells contribute to endogenous brain defenses after acute neonatal focal stroke. J. Neurosci..

[28] Breckwoldt M.O., Chen J.W., Stangenberg L., Aikawa E., Rodriguez E., Qiu S., Moskowitz M.A., Weissleder R. (2008). Tracking the inflammatory response in stroke in vivo by sensing the enzyme myeloperoxidase. Proc. Natl. Acad. Sci. USA.

[29] Kaito M., Araya S., Gondo Y., Fujita M., Minato N., Nakanishi M., Matsui M. (2013). Relevance of distinct monocyte subsets to clinical course of ischemic stroke patients. PLoS ONE.

[30] Swirski F.K., Nahrendorf M., Etzrodt M., Wildgruber M., Cortez-Retamozo V., Panizzi P., Figueiredo J.L., Kohler R.H., Chudnovskiy A., Waterman P. (2009). Identification of splenic reservoir monocytes and their deployment to inflammatory sites. Science.

[31] Offner H., Subramanian S., Parker S.M., Wang C., Afentoulis M.E., Lewis A., Vandenbark A.A., Hurn P.D. (2006). Splenic atrophy in experimental stroke is accompanied by increased regulatory T cells and circulating macrophages. J. Immunol..

[32] Vendrame M., Gemma C., Pennypacker K.R., Bickford P.C., Davis Sanberg C., Sanberg P.R., Willing A.E. (2006). Cord blood rescues stroke-induced changes in splenocyte phenotype and function. Exp. Neurol..

[33] Ajmo C.T., Vernon D.O., Collier L., Hall A.A., Garbuzova-Davis S., Willing A., Pennypacker K.R. (2008). The spleen contributes to stroke-induced neurodegeneration. J. Neurosci. Res.

[34] Dotson A.L., Wang J., Saugstad J., Murphy S.J., Offner H. (2015). Splenectomy reduces infarct volume and neuroinflammation in male but not female mice in experimental stroke. J. Neuroimmunol..

[35] Ostrowski R.P., Schulte R.W., Nie Y., Ling T., Lee T., Manaenko A., Gridley D.S., Zhang J.H. (2012). Acute splenic irradiation reduces brain injury in the rat focal ischemic stroke model. Transl. Stroke Res..

[36] Kim E., Yang J., Beltran C.D., Cho S. (2014). Role of spleen-derived monocytes/macrophages in acute ischemic brain injury. J. Cereb. Blood Flow Metab..

[37] Perego C., Fumagalli S., de Simoni M.G. (2011). Temporal pattern of expression and colocalization of microglia/macrophage phenotype markers following brain ischemic injury in mice. J. Neuroinflamm..

[38] Gliem M., Mausberg A.K., Lee J.I., Simiantonakis I., van Rooijen N., Hartung H.P., Jander S. (2012). Macrophages prevent hemorrhagic infarct transformation in murine stroke models. Ann. Neurol..

[39] Chu H.X., Broughton B.R., Kim H.A., Lee S., Drummond G.R., Sobey C.G. (2015). Evidence that Ly6C_hi_ monocytes are protective in acute ischemic stroke by promoting M2 macrophage polarization. Stroke.

[40] Kleinschnitz C., Schwab N., Kraft P., Hagedorn I., Dreykluft A., Schwarz T., Austinat M., Nieswandt B., Wiendl H., Stoll G. (2010). Early detrimental T-cell effects in experimental cerebral ischemia are neither related to adaptive immunity nor thrombus formation. Blood.

[41] Becker K., Kindrick D., McCarron R., Hallenbeck J., Winn R. (2003). Adoptive transfer of myelin basic protein-tolerized splenocytes to naive animals reduces infarct size: A role for lymphocytes in ischemic brain injury?. Stroke.

[42] Schroeter M., Jander S., Witte O.W., Stoll G. (1994). Local immune responses in the rat cerebral cortex after middle cerebral artery occlusion. J. Neuroimmunol..

[43] Yilmaz G., Arumugam T.V., Stokes K.Y., Granger D.N. (2006). Role of T lymphocytes and interferon-γ in ischemic stroke. Circulation.

[44] Shichita T., Sugiyama Y., Ooboshi H., Sugimori H., Nakagawa R., Takada I., Iwaki T., Okada Y., Iida M., Cua D.J. (2009). Pivotal role of cerebral interleukin-17-producing gammadeltat cells in the delayed phase of ischemic brain injury. Nat. Med..

[45] Li G.Z., Zhong D., Yang L.M., Sun B., Zhong Z.H., Yin Y.H., Cheng J., Yan B.B., Li H.L. (2005). Expression of interleukin-17 in ischemic brain tissue. Scand. J. Immunol..

[46] Liesz A., Suri-Payer E., Veltkamp C., Doerr H., Sommer C., Rivest S., Giese T., Veltkamp R. (2009). Regulatory T cells are key cerebroprotective immunomodulators in acute experimental stroke. Nat. Med..

[47] Ren X., Akiyoshi K., Dziennis S., Vandenbark A.A., Herson P.S., Hurn P.D., Offner H. (2011). Regulatory B cells limit cns inflammation and neurologic deficits in murine experimental stroke. J. Neurosci..

[48] Lambertsen K.L., Biber K., Finsen B. (2012). Inflammatory cytokines in experimental and human stroke. J. Cereb. Blood Flow Metab..

[49] Lambertsen K.L., Meldgaard M., Ladeby R., Finsen B. (2005). A quantitative study of microglial-macrophage synthesis of tumor necrosis factor during acute and late focal cerebral ischemia in mice. J. Cereb. Blood Flow Metab..

[50] Clausen B.H., Lambertsen K.L., Meldgaard M., Finsen B. (2005). A quantitative in situ hybridization and polymerase chain reaction study of microglial-macrophage expression of interleukin-1β mRNA following permanent middle cerebral artery occlusion in mice. Neuroscience.

[51] Lambertsen K.L., Clausen B.H., Babcock A.A., Gregersen R., Fenger C., Nielsen H.H., Haugaard L.S., Wirenfeldt M., Nielsen M., Dagnaes-Hansen F. (2009). Microglia protect neurons against ischemia by synthesis of tumor necrosis factor. J. Neurosci..

[52] Murray K.N., Parry-Jones A.R., Allan S.M. (2015). Interleukin-1 and acute brain injury. Front. Cell. Neurosci..

[53] Luheshi N.M., Kovacs K.J., Lopez-Castejon G., Brough D., Denes A. (2011). Interleukin-1α expression precedes IL-1β after ischemic brain injury and is localised to areas of focal neuronal loss and penumbral tissues. J. Neuroinflamm..

[54] Herx L.M., Yong V.W. (2001). Interleukin-1β is required for the early evolution of reactive astrogliosis following CNS lesion. J. Neuropathol. Exp. Neurol..

[55] Thornton P., Pinteaux E., Allan S.M., Rothwell N.J. (2008). Matrix metalloproteinase-9 and urokinase plasminogen activator mediate interleukin-1-induced neurotoxicity. Mol. Cell. Neurosci..

[56] Allen C., Thornton P., Denes A., McColl B.W., Pierozynski A., Monestier M., Pinteaux E., Rothwell N.J., Allan S.M. (2012). Neutrophil cerebrovascular transmigration triggers rapid neurotoxicity through release of proteases associated with decondensed DNA. J. Immunol..

[57] Amantea D., Micieli G., Tassorelli C., Cuartero M.I., Ballesteros I., Certo M., Moro M.A., Lizasoain I., Bagetta G. (2015). Rational modulation of the innate immune system for neuroprotection in ischemic stroke. Front. Neurosci..

[58] Grilli M., Barbieri I., Basudev H., Brusa R., Casati C., Lozza G., Ongini E. (2000). Interleukin-10 modulates neuronal threshold of vulnerability to ischaemic damage. Eur. J. Neurosci..

[59] Frenkel D., Huang Z., Maron R., Koldzic D.N., Moskowitz M.A., Weiner H.L. (2005). Neuroprotection by IL-10-producing MOG CD4^+^ T cells following ischemic stroke. J. Neurol. Sci..

[60] Yan J., Greer J.M., McCombe P.A. (2012). Prolonged elevation of cytokine levels after human acute ischaemic stroke with evidence of individual variability. J. Neuroimmunol..

[61] Doyle K.P., Cekanaviciute E., Mamer L.E., Buckwalter M.S. (2010). TGFβ signaling in the brain increases with aging and signals to astrocytes and innate immune cells in the weeks after stroke. J. Neuroinflamm..

[62] Zaremba J., Losy J. (2001). Early TNF-α levels correlate with ischaemic stroke severity. Acta Neurol. Scand..

[63] Beridze M., Sanikidze T., Shakarishvili R., Intskirveli N., Bornstein N.M. (2011). Selected acute phase CSF factors in ischemic stroke: Findings and prognostic value. BMC Neurol..

[64] Waje-Andreassen U., Krakenes J., Ulvestad E., Thomassen L., Myhr K.M., Aarseth J., Vedeler C.A. (2005). IL-6: An early marker for outcome in acute ischemic stroke. Acta Neurol. Scand..

[65] Huang J., Li Y., Tang Y., Tang G., Yang G.Y., Wang Y. (2013). CXCR4 antagonist AMD3100 protects blood-brain barrier integrity and reduces inflammatory response after focal ischemia in mice. Stroke.

[66] Ruscher K., Kuric E., Liu Y., Walter H.L., Issazadeh-Navikas S., Englund E., Wieloch T. (2013). Inhibition of CXCL12 signaling attenuates the postischemic immune response and improves functional recovery after stroke. J. Cereb. Blood Flow Metab..

[67] Wang Y., Huang J., Li Y., Yang G.Y. (2012). Roles of chemokine CXCL12 and its receptors in ischemic stroke. Curr. Drug Targets.

[68] Shyu W.C., Lin S.Z., Yen P.S., Su C.Y., Chen D.C., Wang H.J., Li H. (2008). Stromal cell-derived factor-1α promotes neuroprotection, angiogenesis, and mobilization/homing of bone marrow-derived cells in stroke rats. J. Pharmacol. Exp. Ther..

[69] Denes A., Ferenczi S., Halasz J., Kornyei Z., Kovacs K.J. (2008). Role of CX3CR1 (fractalkine receptor) in brain damage and inflammation induced by focal cerebral ischemia in mouse. J. Cereb. Blood Flow Metab..

[70] Cipriani R., Villa P., Chece G., Lauro C., Paladini A., Micotti E., Perego C., de Simoni M.G., Fredholm B.B., Eusebi F. (2011). CX3CL1 is neuroprotective in permanent focal cerebral ischemia in rodents. J. Neurosci..

[71] Tang Z., Gan Y., Liu Q., Yin J.X., Liu Q., Shi J., Shi F.D. (2014). CX3CR1 deficiency suppresses activation and neurotoxicity of microglia/macrophage in experimental ischemic stroke. J. Neuroinflamm..

[72] Donohue M.M., Cain K., Zierath D., Shibata D., Tanzi P.M., Becker K.J. (2012). Higher plasma fractalkine is associated with better 6-month outcome from ischemic stroke. Stroke.

[73] Rosito M., Lauro C., Chece G., Porzia A., Monaco L., Mainiero F., Catalano M., Limatola C., Trettel F. (2014). Trasmembrane chemokines CX3CL1 and CXCL16 drive interplay between neurons, microglia and astrocytes to counteract pMCAO and excitotoxic neuronal death. Front. Cell. Neurosci..

[74] Schilling M., Strecker J.K., Ringelstein E.B., Schabitz W.R., Kiefer R. (2009). The role of cc chemokine receptor 2 on microglia activation and blood-borne cell recruitment after transient focal cerebral ischemia in mice. Brain Res..

[75] Che X., Ye W., Panga L., Wu D.C., Yang G.Y. (2001). Monocyte chemoattractant protein-1 expressed in neurons and astrocytes during focal ischemia in mice. Brain Res..

[76] Strecker J.K., Minnerup J., Schutte-Nutgen K., Gess B., Schabitz W.R., Schilling M. (2013). Monocyte chemoattractant protein-1-deficiency results in altered blood-brain barrier breakdown after experimental stroke. Stroke.

[77] Yan Y.P., Sailor K.A., Lang B.T., Park S.W., Vemuganti R., Dempsey R.J. (2007). Monocyte chemoattractant protein-1 plays a critical role in neuroblast migration after focal cerebral ischemia. J. Cereb. Blood Flow Metab..

[78] Strecker J.K., Minnerup J., Gess B., Ringelstein E.B., Schabitz W.R., Schilling M. (2011). Monocyte chemoattractant protein-1-deficiency impairs the expression of IL-6, IL-1β and G-CSF after transient focal ischemia in mice. PLoS ONE.

[79] Schuette-Nuetgen K., Strecker J.K., Minnerup J., Ringelstein E.B., Schilling M. (2012). MCP-1/CCR-2-double-deficiency severely impairs the migration of hematogenous inflammatory cells following transient cerebral ischemia in mice. Exp. Neurol..

[80] Carbone F., Teixeira P.C., Braunersreuther V., Mach F., Vuilleumier N., Montecucco F. (2015). Pathophysiology and treatments of oxidative injury in ischemic stroke: Focus on the phagocytic NADPH oxidase 2. Antioxid. Redox Signal..

[81] Lambeth J.D. (2004). Nox enzymes and the biology of reactive oxygen. Nat. Rev. Immunol..

[82] Wang Q., Tang X.N., Yenari M.A. (2007). The inflammatory response in stroke. J. Neuroimmunol..

[83] Vallet P., Charnay Y., Steger K., Ogier-Denis E., Kovari E., Herrmann F., Michel J.P., Szanto I. (2005). Neuronal expression of the NADPH oxidase NOX4, and its regulation in mouse experimental brain ischemia. Neuroscience.

[84] Drummond G.R., Sobey C.G. (2014). Endothelial NADPH oxidases: Which NOX to target in vascular disease?. Trends Endocrinol. Metab..

[85] Douglas G., Bendall J.K., Crabtree M.J., Tatham A.L., Carter E.E., Hale A.B., Channon K.M. (2012). Endothelial-specific NOX2 overexpression increases vascular superoxide and macrophage recruitment in ApoE^−/−^ mice. Cardiovasc. Res..

[86] Ray R., Murdoch C.E., Wang M., Santos C.X., Zhang M., Alom-Ruiz S., Anilkumar N., Ouattara A., Cave A.C., Walker S.J. (2011). Endothelial NOX4 NADPH oxidase enhances vasodilatation and reduces blood pressure in vivo. Arterioscler. Thromb. Vasc. Biol..

[87] Walder C.E., Green S.P., Darbonne W.C., Mathias J., Rae J., Dinauer M.C., Curnutte J.T., Thomas G.R. (1997). Ischemic stroke injury is reduced in mice lacking a functional NADPH oxidase. Stroke.

[88] Thomas S.R., Witting P.K., Drummond G.R. (2008). Redox control of endothelial function and dysfunction: Molecular mechanisms and therapeutic opportunities. Antioxid. Redox Signal..

[89] Craige S.M., Chen K., Pei Y., Li C., Huang X., Chen C., Shibata R., Sato K., Walsh K., Keaney J.F. (2011). NADPH oxidase 4 promotes endothelial angiogenesis through endothelial nitric oxide synthase activation. Circulation.

[90] Kleinschnitz C., Grund H., Wingler K., Armitage M.E., Jones E., Mittal M., Barit D., Schwarz T., Geis C., Kraft P. (2010). Post-stroke inhibition of induced NADPH oxidase type 4 prevents oxidative stress and neurodegeneration. PLoS Biol..

[91] Lakhan S.E., Kirchgessner A., Hofer M. (2009). Inflammatory mechanisms in ischemic stroke: Therapeutic approaches. J. Transl. Med..

[92] Yang Z.J., Xie Y., Bosco G.M., Chen C., Camporesi E.M. (2010). Hyperbaric oxygenation alleviates MCAO-induced brain injury and reduces hydroxyl radical formation and glutamate release. Eur. J. Appl. Physiol..

[93] Cheret C., Gervais A., Lelli A., Colin C., Amar L., Ravassard P., Mallet J., Cumano A., Krause K.H., Mallat M. (2008). Neurotoxic activation of microglia is promoted by a NOX1-dependent NADPH oxidase. J. Neurosci..

[94] Kim H.S., Cho I.H., Kim J.E., Shin Y.J., Jeon J.H., Kim Y., Yang Y.M., Lee K.H., Lee J.W., Lee W.J. (2008). Ethyl pyruvate has an anti-inflammatory effect by inhibiting ROS-dependent STAT signaling in activated microglia. Free Radic. Biol. Med..

[95] Kaspar J.W., Niture S.K., Jaiswal A.K. (2009). Nrf2:INrf2 (Keap1) signaling in oxidative stress. Free Radic. Biol. Med..

[96] Rizzo M.T., Leaver H.A. (2010). Brain endothelial cell death: Modes, signaling pathways, and relevance to neural development, homeostasis, and disease. Mol. Neurobiol..

[97] Liesz A., Dalpke A., Mracsko E., Antoine D.J., Roth S., Zhou W., Yang H., Na S.Y., Akhisaroglu M., Fleming T. (2015). DAMP signaling is a key pathway inducing immune modulation after brain injury. J. Neurosci..

[98] Zhang Q., Raoof M., Chen Y., Sumi Y., Sursal T., Junger W., Brohi K., Itagaki K., Hauser C.J. (2010). Circulating mitochondrial damps cause inflammatory responses to injury. Nature.

[99] Maeda A., Fadeel B. (2014). Mitochondria released by cells undergoing TNF-α-induced necroptosis act as danger signals. Cell Death Dis..

[100] Kawai T., Akira S. (2010). The role of pattern-recognition receptors in innate immunity: Update on toll-like receptors. Nat. Immunol..

[101] Hyakkoku K., Hamanaka J., Tsuruma K., Shimazawa M., Tanaka H., Uematsu S., Akira S., Inagaki N., Nagai H., Hara H. (2010). Toll-like receptor 4 (TLR4), but not TLR3 or TLR9, knock-out mice have neuroprotective effects against focal cerebral ischemia. Neuroscience.

[102] Ceruti S., Villa G., Genovese T., Mazzon E., Longhi R., Rosa P., Bramanti P., Cuzzocrea S., Abbracchio M.P. (2009). The P2Y-like receptor GPR17 as a sensor of damage and a new potential target in spinal cord injury. Brain.

[103] Yang F., Wang Z., Wei X., Han H., Meng X., Zhang Y., Shi W., Li F., Xin T., Pang Q. (2014). NLRP3 deficiency ameliorates neurovascular damage in experimental ischemic stroke. J. Cereb. Blood Flow Metab..

[104] Weismann D., Binder C.J. (2012). The innate immune response to products of phospholipid peroxidation. Biochim. Biophys. Acta.

[105] Abe T., Shimamura M., Jackman K., Kurinami H., Anrather J., Zhou P., Iadecola C. (2010). Key role of CD36 in toll-like receptor 2 signaling in cerebral ischemia. Stroke.

[106] Zhong Z., Zhai Y., Liang S., Mori Y., Han R., Sutterwala F.S., Qiao L. (2013). TRPM2 links oxidative stress to NLRP3 inflammasome activation. Nat. Commun..

[107] Iyer S.S., He Q., Janczy J.R., Elliott E.I., Zhong Z., Olivier A.K., Sadler J.J., Knepper-Adrian V., Han R., Qiao L. (2013). Mitochondrial cardiolipin is required for NLRP3 inflammasome activation. Immunity.

[108] Lotze M.T., Tracey K.J. (2005). High-mobility group box 1 protein (HMGB1): Nuclear weapon in the immune arsenal. Nat. Rev. Immunol..

[109] Rouhiainen A., Kuja-Panula J., Wilkman E., Pakkanen J., Stenfors J., Tuominen R.K., Lepantalo M., Carpen O., Parkkinen J., Rauvala H. (2004). Regulation of monocyte migration by amphoterin (HMGB1). Blood.

[110] Hu J., Liu B., Zhao Q., Jin P., Hua F., Zhang Z., Liu Y., Zan K., Cui G., Ye X. (2016). Bone marrow stromal cells inhibits HMGB1-mediated inflammation after stroke in type 2 diabetic rats. Neuroscience.

[111] Shichita T., Hasegawa E., Kimura A., Morita R., Sakaguchi R., Takada I., Sekiya T., Ooboshi H., Kitazono T., Yanagawa T. (2012). Peroxiredoxin family proteins are key initiators of post-ischemic inflammation in the brain. Nat. Med..

[112] Loser K., Vogl T., Voskort M., Lueken A., Kupas V., Nacken W., Klenner L., Kuhn A., Foell D., Sorokin L. (2010). The Toll-like receptor 4 ligands Mrp8 and Mrp14 are crucial in the development of autoreactive CD8^+^ T cells. Nat. Med..

[113] Qiang X., Yang W.L., Wu R., Zhou M., Jacob A., Dong W., Kuncewitch M., Ji Y., Yang H., Wang H. (2013). Cold-inducible RNA-binding protein (CIRP) triggers inflammatory responses in hemorrhagic shock and sepsis. Nat. Med..

[114] Yang Y., Liu B., Dai J., Srivastava P.K., Zammit D.J., Lefrancois L., Li Z. (2007). Heat shock protein GP96 is a master chaperone for toll-like receptors and is important in the innate function of macrophages. Immunity.

[115] Ehrenstein M.R., Notley C.A. (2010). The importance of natural IgM: Scavenger, protector and regulator. Nat. Rev. Immunol..

[116] Vargas M.E., Watanabe J., Singh S.J., Robinson W.H., Barres B.A. (2010). Endogenous antibodies promote rapid myelin clearance and effective axon regeneration after nerve injury. Proc. Natl. Acad. Sci. USA.

[117] Bornstein N.M., Aronovich B., Korczyn A.D., Shavit S., Michaelson D.M., Chapman J. (2001). Antibodies to brain antigens following stroke. Neurology.

[118] Soussan L., Tchernakov K., Bachar-Lavi O., Yuvan T., Wertman E., Michaelson D.M. (1994). Antibodies to different isoforms of the heavy neurofilament protein (NF-H) in normal aging and Alzheimer’s disease. Mol. Neurobiol..

[119] Dambinova S.A., Khounteev G.A., Izykenova G.A., Zavolokov I.G., Ilyukhina A.Y., Skoromets A.A. (2003). Blood test detecting autoantibodies to *N*-methyl-d-aspartate neuroreceptors for evaluation of patients with transient ischemic attack and stroke. Clin. Chem..

[120] Kimura A., Sakurai T., Yamada M., Koumura A., Hayashi Y., Tanaka Y., Hozumi I., Takemura M., Seishima M., Inuzuka T. (2012). Elevated anti-heat shock protein 60 antibody titer is related to white matter hyperintensities. J. Stroke Cerebrovasc. Dis..

[121] Becker K.J., Kalil A.J., Tanzi P., Zierath D.K., Savos A.V., Gee J.M., Hadwin J., Carter K.T., Shibata D., Cain K.C. (2011). Autoimmune responses to the brain after stroke are associated with worse outcome. Stroke.

[122] Shibata D., Cain K., Tanzi P., Zierath D., Becker K. (2012). Myelin basic protein autoantibodies, white matter disease and stroke outcome. J. Neuroimmunol..

[123] Schoppet M., Sattler A.M., Schaefer J.R., Herzum M., Maisch B., Hofbauer L.C. (2003). Increased osteoprotegerin serum levels in men with coronary artery disease. J. Clin. Endocrinol. Metab..

[124] Osako M.K., Nakagami H., Koibuchi N., Shimizu H., Nakagami F., Koriyama H., Shimamura M., Miyake T., Rakugi H., Morishita R. (2010). Estrogen inhibits vascular calcification via vascular RANKL system: Common mechanism of osteoporosis and vascular calcification. Circ. Res..

[125] Maruyama K., Takada Y., Ray N., Kishimoto Y., Penninger J.M., Yasuda H., Matsuo K. (2006). Receptor activator of NF-κB ligand and osteoprotegerin regulate proinflammatory cytokine production in mice. J. Immunol..

[126] Ferrari-Lacraz S., Ferrari S. (2011). Do RANKL inhibitors (denosumab) affect inflammation and immunity?. Osteoporos. Int..

[127] Ustundag M., Orak M., Guloglu C., Tamam Y., Sayhan M.B., Kale E. (2011). The role of serum osteoprotegerin and S-100 protein levels in patients with acute ischaemic stroke: Determination of stroke subtype, severity and mortality. J. Int. Med. Res..

[128] Jensen J.K., Ueland T., Atar D., Gullestad L., Mickley H., Aukrust P., Januzzi J.L. (2010). Osteoprotegerin concentrations and prognosis in acute ischaemic stroke. J. Intern. Med..

[129] Guldiken B., Guldiken S., Turgut B., Turgut N., Demir M., Celik Y., Arikan E., Tugrul A. (2007). Serum osteoprotegerin levels in patients with acute atherothrombotic stroke and lacunar infarct. Thromb. Res..

[130] Kim B.J., Lee S.H., Ryu W.S., Kim C.K., Yoon B.W. (2012). Adipocytokines and ischemic stroke: Differential associations between stroke subtypes. J. Neurol. Sci..

[131] Zhang F., Wang S., Signore A.P., Chen J. (2007). Neuroprotective effects of leptin against ischemic injury induced by oxygen-glucose deprivation and transient cerebral ischemia. Stroke.

[132] Carbone F., Burger F., Roversi G., Tamborino C., Casetta I., Seraceni S., Trentini A., Padroni M., Bertolotto M., Dallegri F. (2015). Leptin/adiponectin ratio predicts poststroke neurological outcome. Eur. J. Clin. Investig..

[133] Valerio A., Dossena M., Bertolotti P., Boroni F., Sarnico I., Faraco G., Chiarugi A., Frontini A., Giordano A., Liou H.C. (2009). Leptin is induced in the ischemic cerebral cortex and exerts neuroprotection through NF-κB/c-Rel-dependent transcription. Stroke.

[134] Zhang J., Deng Z., Liao J., Song C., Liang C., Xue H., Wang L., Zhang K., Yan G. (2013). Leptin attenuates cerebral ischemia injury through the promotion of energy metabolism via the PI3K/Akt pathway. J. Cereb. Blood Flow Metab..

[135] Avraham Y., Davidi N., Lassri V., Vorobiev L., Kabesa M., Dayan M., Chernoguz D., Berry E., Leker R.R. (2011). Leptin induces neuroprotection neurogenesis and angiogenesis after stroke. Curr. Neurovasc. Res..

[136] Zhang J.Y., Si Y.L., Liao J., Yan G.T., Deng Z.H., Xue H., Wang L.H., Zhang K. (2012). Leptin administration alleviates ischemic brain injury in mice by reducing oxidative stress and subsequent neuronal apoptosis. J. Trauma Acute Care Surg..

[137] Efstathiou S.P., Tsioulos D.I., Tsiakou A.G., Gratsias Y.E., Pefanis A.V., Mountokalakis T.D. (2005). Plasma adiponectin levels and five-year survival after first-ever ischemic stroke. Stroke.

[138] Shen L., Miao J., Yuan F., Zhao Y., Tang Y., Wang Y., Zhao Y., Yang G.Y. (2013). Overexpression of adiponectin promotes focal angiogenesis in the mouse brain following middle cerebral artery occlusion. Gene Ther..

[139] Miao J., Shen L.H., Tang Y.H., Wang Y.T., Tao M.X., Jin K.L., Zhao Y.J., Yang G.Y. (2013). Overexpression of adiponectin improves neurobehavioral outcomes after focal cerebral ischemia in aged mice. CNS Neurosci. Ther..

[140] Scatena M., Liaw L., Giachelli C.M. (2007). Osteopontin: A multifunctional molecule regulating chronic inflammation and vascular disease. Arterioscler. Thromb. Vasc. Biol..

[141] Mendioroz M., Fernandez-Cadenas I., Rosell A., Delgado P., Domingues-Montanari S., Ribo M., Penalba A., Quintana M., Alvarez-Sabin J., Montaner J. (2011). Osteopontin predicts long-term functional outcome among ischemic stroke patients. J. Neurol..

[142] Carbone F., Vuilleumier N., Burger F., Roversi G., Tamborino C., Casetta I., Seraceni S., Trentini A., Padroni M., Dallegri F. (2015). Serum osteopontin levels are upregulated and predict disability after an ischaemic stroke. Eur. J. Clin. Investig..

[143] Meller R., Stevens S.L., Minami M., Cameron J.A., King S., Rosenzweig H., Doyle K., Lessov N.S., Simon R.P., Stenzel-Poore M.P. (2005). Neuroprotection by osteopontin in stroke. J. Cereb. Blood Flow Metab..

[144] Doyle K.P., Yang T., Lessov N.S., Ciesielski T.M., Stevens S.L., Simon R.P., King J.S., Stenzel-Poore M.P. (2008). Nasal administration of osteopontin peptide mimetics confers neuroprotection in stroke. J. Cereb. Blood Flow Metab..

[145] Gliem M., Krammes K., Liaw L., van Rooijen N., Hartung H.P., Jander S. (2015). Macrophage-derived osteopontin induces reactive astrocyte polarization and promotes re-establishment of the blood brain barrier after ischemic stroke. Glia.

[146] Dassan P., Keir G., Brown M.M. (2009). Criteria for a clinically informative serum biomarker in acute ischaemic stroke: A review of S100B. Cerebrovasc. Dis..

[147] Ye H., Wang L., Yang X.K., Fan L.P., Wang Y.G., Guo L. (2015). Serum S100B levels may be associated with cerebral infarction: A meta-analysis. J. Neurol. Sci..

[148] Kazmierski R., Michalak S., Wencel-Warot A., Nowinski W.L. (2012). Serum tight-junction proteins predict hemorrhagic transformation in ischemic stroke patients. Neurology.

[149] Tsai N.W., Chang Y.T., Huang C.R., Lin Y.J., Lin W.C., Cheng B.C., Su C.M., Chiang Y.F., Chen S.F., Huang C.C. (2014). Association between oxidative stress and outcome in different subtypes of acute ischemic stroke. BioMed Res. Int..

[150] Lorente L., Martin M.M., Abreu-Gonzalez P., Ramos L., Argueso M., Sole-Violan J., Riano-Ruiz M., Jimenez A. (2015). Serum malondialdehyde levels in patients with malignant middle cerebral artery infarction are associated with mortality. PLoS ONE.

[151] Bharosay A., Bharosay V.V., Varma M., Saxena K., Sodani A., Saxena R. (2012). Correlation of brain biomarker neuron specific enolase (NSE) with degree of disability and neurological worsening in cerebrovascular stroke. Indian J. Clin. Biochem..

[152] Singh H.V., Pandey A., Shrivastava A.K., Raizada A., Singh S.K., Singh N. (2013). Prognostic value of neuron specific enolase and IL-10 in ischemic stroke and its correlation with degree of neurological deficit. Clin. Chim. Acta.

[153] Zaheer S., Beg M., Rizvi I., Islam N., Ullah E., Akhtar N. (2013). Correlation between serum neuron specific enolase and functional neurological outcome in patients of acute ischemic stroke. Ann. Indian Acad. Neurol..

[154] Park S.Y., Kim M.H., Kim O.J., Ahn H.J., Song J.Y., Jeong J.Y., Oh S.H. (2013). Plasma heart-type fatty acid binding protein level in acute ischemic stroke: Comparative analysis with plasma S100B level for diagnosis of stroke and prediction of long-term clinical outcome. Clin. Neurol. Neurosurg..

[155] Kim B.J., Kim Y.J., Ahn S.H., Kim N.Y., Kang D.W., Kim J.S., Kwon S.U. (2014). The second elevation of neuron-specific enolase peak after ischemic stroke is associated with hemorrhagic transformation. J. Stroke Cerebrovasc. Dis..

[156] Lu K., Xu X., Cui S., Wang F., Zhang B., Zhao Y. (2015). Serum neuron specific enolase level as a predictor of prognosis in acute ischemic stroke patients after intravenous thrombolysis. J. Neurol. Sci..

[157] Haupt W.F., Chopan G., Sobesky J., Liu W.C., Dohmen C. (2016). Prognostic value of somatosensory evoked potentials, neuron-specific enolase, and S100 for short-term outcome in ischemic stroke. J. Neurophysiol..

[158] Ozkan A.K., Yemisci O.U., Saracgil Cosar S.N., Oztop P., Turhan N. (2013). Can high-sensitivity C-reactive protein and ferritin predict functional outcome in acute ischemic stroke? A prospective study. Top. Stroke Rehabil..

[159] Taheraghdam A., Aminnejad S., Pashapour A., Rikhtegar R., Ghabili K. (2013). Is there a correlation between hs-CRP levels and functional outcome of ischemic stroke?. Pak. J. Med. Sci..

[160] VanGilder R.L., Davidov D.M., Stinehart K.R., Huber J.D., Turner R.C., Wilson K.S., Haney E., Davis S.M., Chantler P.D., Theeke L. (2014). C-reactive protein and long-term ischemic stroke prognosis. J. Clin. Neurosci..

[161] Karlinski M., Bembenek J., Grabska K., Kobayashi A., Baranowska A., Litwin T., Czlonkowska A. (2014). Routine serum C-reactive protein and stroke outcome after intravenous thrombolysis. Acta Neurol. Scand..

[162] Pandey A., Shrivastava A.K., Saxena K. (2014). Neuron specific enolase and c-reactive protein levels in stroke and its subtypes: Correlation with degree of disability. Neurochem. Res..

[163] Li Y.M., Liu X.Y. (2015). Serum levels of procalcitonin and high sensitivity C-reactive protein are associated with long-term mortality in acute ischemic stroke. J. Neurol. Sci..

[164] Rocco A., Ringleb P.A., Grittner U., Nolte C.H., Schneider A., Nagel S. (2015). Follow-up C-reactive protein level is more strongly associated with outcome in stroke patients than admission levels. Neurol. Sci..

[165] Deng W.J., Shen R.L., Li M., Teng J.F. (2015). Relationship between procalcitonin serum levels and functional outcome in stroke patients. Cell Mol. Neurobiol..

[166] Wang C., Gao L., Zhang Z.G., Li Y.Q., Yang Y.L., Chang T., Zheng L.L., Zhang X.Y., Man M.H., Li L.H. (2016). Procalcitonin is a stronger predictor of long-term functional outcome and mortality than high-sensitivity C-reactive protein in patients with ischemic stroke. Mol. Neurobiol..

[167] Matsuo R., Ago T., Hata J., Wakisaka Y., Kuroda J., Kuwashiro T., Kitazono T., Kamouchi M. (2016). Fukuoka Stroke Registry Investigators. Plasma C-reactive protein and clinical outcomes after acute ischemic stroke: A prospective observational study. PLoS ONE.

[168] Geng H.H., Wang X.W., Fu R.L., Jing M.J., Huang L.L., Zhang Q., Wang X.X., Wang P.X. (2016). The relationship between C-reactive protein level and discharge outcome in patients with acute ischemic stroke. Int. J. Environ. Res. Public Health.

[169] Whiteley W., Jackson C., Lewis S., Lowe G., Rumley A., Sandercock P., Wardlaw J., Dennis M., Sudlow C. (2009). Inflammatory markers and poor outcome after stroke: A prospective cohort study and systematic review of interleukin-6. PLoS Med..

[170] Park S.Y., Kim J., Kim O.J., Kim J.K., Song J., Shin D.A., Oh S.H. (2013). Predictive value of circulating interleukin-6 and heart-type fatty acid binding protein for three months clinical outcome in acute cerebral infarction: Multiple blood markers profiling study. Crit. Care.

[171] Bustamante A., Sobrino T., Giralt D., Garcia-Berrocoso T., Llombart V., Ugarriza I., Espadaler M., Rodriguez N., Sudlow C., Castellanos M. (2014). Prognostic value of blood interleukin-6 in the prediction of functional outcome after stroke: A systematic review and meta-analysis. J. Neuroimmunol..

[172] Pusch G., Debrabant B., Molnar T., Feher G., Papp V., Banati M., Kovacs N., Szapary L., Illes Z. (2015). Early dynamics of P-selectin and interleukin 6 predicts outcomes in ischemic stroke. J. Stroke Cerebrovasc. Dis..

[173] Lehmann M.F., Kallaur A.P., Oliveira S.R., Alfieri D.F., Delongui F., de Sousa Parreira J., de Araujo M.C., Rossato C., de Almeida J.T., Pelegrino L.M. (2015). Inflammatory and metabolic markers and short-time outcome in patients with acute ischemic stroke in relation to toast subtypes. Metab. Brain Dis..

[174] Worthmann H., Tryc A.B., Dirks M., Schuppner R., Brand K., Klawonn F., Lichtinghagen R., Weissenborn K. (2015). Lipopolysaccharide binding protein, interleukin-10, interleukin-6 and C-reactive protein blood levels in acute ischemic stroke patients with post-stroke infection. J. Neuroinflamm..

[175] Fahmi R.M., Elsaid A.F. (2016). Infarction size, interleukin-6, and their interaction are predictors of short-term stroke outcome in young egyptian adults. J. Stroke Cerebrovasc. Dis..

[176] Rodriguez-Yanez M., Castellanos M., Sobrino T., Brea D., Ramos-Cabrer P., Pedraza S., Castineiras J.A., Serena J., Davalos A., Castillo J. (2013). Interleukin-10 facilitates the selection of patients for systemic thrombolysis. BMC Neurol..

[177] Ashour W., Al-Anwar A.D., Kamel A.E., Aidaros M.A. (2016). Predictors of early infection in cerebral ischemic stroke. J. Med. Life.

[178] Carbone F., Vuilleumier N., Bertolotto M., Burger F., Galan K., Roversi G., Tamborino C., Casetta I., Seraceni S., Trentini A. (2015). Treatment with recombinant tissue plasminogen activator (r-TPA) induces neutrophil degranulation in vitro via defined pathways. Vasc. Pharmacol..

[179] Inzitari D., Giusti B., Nencini P., Gori A.M., Nesi M., Palumbo V., Piccardi B., Armillis A., Pracucci G., Bono G. (2013). MMP9 variation after thrombolysis is associated with hemorrhagic transformation of lesion and death. Stroke.

[180] Wiseman S., Marlborough F., Doubal F., Webb D.J., Wardlaw J. (2014). Blood markers of coagulation, fibrinolysis, endothelial dysfunction and inflammation in lacunar stroke versus non-lacunar stroke and non-stroke: Systematic review and meta-analysis. Cerebrovasc. Dis..

[181] Liu L.B., Li M., Zhuo W.Y., Zhang Y.S., Xu A.D. (2015). The role of hs-CRP, d-dimer and fibrinogen in differentiating etiological subtypes of ischemic stroke. PLoS ONE.

[182] Yuan W., Shi Z.H. (2014). The relationship between plasma D-dimer levels and outcome of Chinese acute ischemic stroke patients in different stroke subtypes. J. Neural Transm..

[183] Yang X.Y., Gao S., Ding J., Chen Y., Zhou X.S., Wang J.E. (2014). Plasma d-dimer predicts short-term poor outcome after acute ischemic stroke. PLoS ONE.

[184] Richard S., Lagerstedt L., Burkhard P.R., Debouverie M., Turck N., Sanchez J.C. (2015). E-selectin and vascular cell adhesion molecule-1 as biomarkers of 3-month outcome in cerebrovascular diseases. J. Inflamm..

[185] Wang J., Ning R., Wang Y. (2016). Plasma d-dimer level, the promising prognostic biomarker for the acute cerebral infarction patients. J. Stroke Cerebrovasc. Dis..

[186] Hsu P.J., Chen C.H., Yeh S.J., Tsai L.K., Tang S.C., Jeng J.S. (2016). High plasma d-dimer indicates unfavorable outcome of acute ischemic stroke patients receiving intravenous thrombolysis. Cerebrovasc. Dis..

[187] Jin R., Yang G., Li G. (2010). Molecular insights and therapeutic targets for blood-brain barrier disruption in ischemic stroke: Critical role of matrix metalloproteinases and tissue-type plasminogen activator. Neurobiol. Dis..

[188] Kapural M., Krizanac-Bengez L., Barnett G., Perl J., Masaryk T., Apollo D., Rasmussen P., Mayberg M.R., Janigro D. (2002). Serum S-100β as a possible marker of blood-brain barrier disruption. Brain Res..

[189] Steiner J., Bernstein H.G., Bielau H., Berndt A., Brisch R., Mawrin C., Keilhoff G., Bogerts B. (2007). Evidence for a wide extra-astrocytic distribution of S100B in human brain. BMC Neurosci..

[190] Cherubini A., Ruggiero C., Polidori M.C., Mecocci P. (2005). Potential markers of oxidative stress in stroke. Free Radic. Biol. Med..

[191] Wunderlich M.T., Hanhoff T., Goertler M., Spener F., Glatz J.F., Wallesch C.W., Pelsers M.M. (2005). Release of brain-type and heart-type fatty acid-binding proteins in serum after acute ischaemic stroke. J. Neurol..

[192] Wunderlich M.T., Lins H., Skalej M., Wallesch C.W., Goertler M. (2006). Neuron-specific enolase and tau protein as neurobiochemical markers of neuronal damage are related to early clinical course and long-term outcome in acute ischemic stroke. Clin. Neurol. Neurosurg..

[193] Isgro M.A., Bottoni P., Scatena R. (2015). Neuron-specific enolase as a biomarker: Biochemical and clinical aspects. Adv. Exp. Med. Biol..

[194] Kaptoge S., di Angelantonio E., Lowe G., Pepys M.B., Thompson S.G., Collins R., Danesh J., Emerging Risk Factors Collaboration (2010). C-reactive protein concentration and risk of coronary heart disease, stroke, and mortality: An individual participant meta-analysis. Lancet.

[195] Di Napoli M., Schwaninger M., Cappelli R., Ceccarelli E., di Gianfilippo G., Donati C., Emsley H.C., Forconi S., Hopkins S.J., Masotti L. (2005). Evaluation of C-reactive protein measurement for assessing the risk and prognosis in ischemic stroke: A statement for health care professionals from the crp pooling project members. Stroke.

[196] Bustamante A., Simats A., Vilar-Bergua A., Garcia-Berrocoso T., Montaner J. (2016). Blood/brain biomarkers of inflammation after stroke and their association with outcome: From C-reactive protein to damage-associated molecular patterns. Neurotherapeutics.

[197] Doll D.N., Barr T.L., Simpkins J.W. (2014). Cytokines: Their role in stroke and potential use as biomarkers and therapeutic targets. Aging Dis..

[198] Perini F., Morra M., Alecci M., Galloni E., Marchi M., Toso V. (2001). Temporal profile of serum anti-inflammatory and pro-inflammatory interleukins in acute ischemic stroke patients. Neurol. Sci..

[199] Turner R.J., Sharp F.R. (2016). Implications of MMP9 for blood brain barrier disruption and hemorrhagic transformation following ischemic stroke. Front. Cell. Neurosci..

[200] Singh V., Roth S., Veltkamp R., Liesz A. (2016). HMGB1 as a key mediator of immune mechanisms in ischemic stroke. Antioxid. Redox Signal..

[201] Schulze J., Zierath D., Tanzi P., Cain K., Shibata D., Dressel A., Becker K. (2013). Severe stroke induces long-lasting alterations of high-mobility group box 1. Stroke.

[202] Huang J.M., Hu J., Chen N., Hu M.L. (2013). Relationship between plasma high-mobility group box-1 levels and clinical outcomes of ischemic stroke. J. Crit. Care.

[203] Sapojnikova N., Kartvelishvili T., Asatiani N., Zinkevich V., Kalandadze I., Gugutsidze D., Shakarishvili R., Tsiskaridze A. (2014). Correlation between MMP-9 and extracellular cytokine HMGB1 in prediction of human ischemic stroke outcome. Biochim. Biophys. Acta.

[204] Marousi S.G., Theodorou G.L., Karakantza M., Zampakis P., Papathanasopoulos P., Ellul J. (2010). Acute post-stroke adiponectin in relation to stroke severity, progression and 6 month functional outcome. Neurol. Res..

[205] Kuwashiro T., Ago T., Kamouchi M., Matsuo R., Hata J., Kuroda J., Fukuda K., Sugimori H., Fukuhara M., Awano H. (2014). Significance of plasma adiponectin for diagnosis, neurological severity and functional outcome in ischemic stroke—Research for biomarkers in ischemic stroke (REBIOS). Metabolism.

[206] Vogelgesang A., May V.E., Grunwald U., Bakkeboe M., Langner S., Wallaschofski H., Kessler C., Broker B.M., Dressel A. (2010). Functional status of peripheral blood T-cells in ischemic stroke patients. PLoS ONE.

[207] Yang Q.W., Lu F.L., Zhou Y., Wang L., Zhong Q., Lin S., Xiang J., Li J.C., Fang C.Q., Wang J.Z. (2011). HMBG1 mediates ischemia—Reperfusion injury by TRIF-adaptor independent toll-like receptor 4 signaling. J. Cereb. Blood Flow Metab..

[208] Kanhai D.A., Kranendonk M.E., Uiterwaal C.S., van der Graaf Y., Kappelle L.J., Visseren F.L. (2013). Adiponectin and incident coronary heart disease and stroke. A systematic review and meta-analysis of prospective studies. Obes. Rev..

[209] Arregui M., Buijsse B., Fritsche A., di Giuseppe R., Schulze M.B., Westphal S., Isermann B., Boeing H., Weikert C. (2014). Adiponectin and risk of stroke: Prospective study and meta-analysis. Stroke.

[210] Akasofu S., Sawada K., Kosasa T., Hihara H., Ogura H., Akaike A. (2008). Donepezil attenuates excitotoxic damage induced by membrane depolarization of cortical neurons exposed to veratridine. Eur. J. Pharmacol..

[211] Amin-Hanjani S., Stagliano N.E., Yamada M., Huang P.L., Liao J.K., Moskowitz M.A. (2001). Mevastatin, an HMG-CoA reductase inhibitor, reduces stroke damage and upregulates endothelial nitric oxide synthase in mice. Stroke.

[212] Asahi M., Huang Z., Thomas S., Yoshimura S., Sumii T., Mori T., Qiu J., Amin-Hanjani S., Huang P.L., Liao J.K. (2005). Protective effects of statins involving both eNOS and tPA in focal cerebral ischemia. J. Cereb. Blood Flow Metab..

[213] Becker K., Kindrick D., Relton J., Harlan J., Winn R. (2001). Antibody to the α4 integrin decreases infarct size in transient focal cerebral ischemia in rats. Stroke.

[214] Campos F., Qin T., Castillo J., Seo J.H., Arai K., Lo E.H., Waeber C. (2013). Fingolimod reduces hemorrhagic transformation associated with delayed tissue plasminogen activator treatment in a mouse thromboembolic model. Stroke.

[215] Chen J., Zhang Z.G., Li Y., Wang Y., Wang L., Jiang H., Zhang C., Lu M., Katakowski M., Feldkamp C.S. (2003). Statins induce angiogenesis, neurogenesis, and synaptogenesis after stroke. Ann. Neurol..

[216] Chen W., Guo Y., Yang W., Zheng P., Zeng J., Tong W. (2015). Protective effect of ginsenoside Rb1 on integrity of blood-brain barrier following cerebral ischemia. Exp. Brain Res..

[217] Cho T.H., Aguettaz P., Campuzano O., Charriaut-Marlangue C., Riou A., Berthezene Y., Nighoghossian N., Ovize M., Wiart M., Chauveau F. (2013). Pre- and post-treatment with cyclosporine A in a rat model of transient focal cerebral ischaemia with multimodal MRI screening. Int. J. Stroke.

[218] Dong X., Zheng L., Lu S., Yang Y. (2015). Neuroprotective effects of pretreatment of ginsenoside Rb1 on severe cerebral ischemia-induced injuries in aged mice: Involvement of anti-oxidant signaling. Geriatr. Gerontol. Int..

[219] Endres M., Laufs U., Huang Z., Nakamura T., Huang P., Moskowitz M.A., Liao J.K. (1998). Stroke protection by 3-hydroxy-3-methylglutaryl (HMG)-CoA reductase inhibitors mediated by endothelial nitric oxide synthase. Proc. Natl. Acad. Sci. USA.

[220] Espinera A.R., Ogle M.E., Gu X., Wei L. (2013). Citalopram enhances neurovascular regeneration and sensorimotor functional recovery after ischemic stroke in mice. Neuroscience.

[221] Fujiwara N., Som A.T., Pham L.D., Lee B.J., Mandeville E.T., Lo E.H., Arai K. (2016). A free radical scavenger edaravone suppresses systemic inflammatory responses in a rat transient focal ischemia model. Neurosci. Lett..

[222] Kawashima S., Yamashita T., Miwa Y., Ozaki M., Namiki M., Hirase T., Inoue N., Hirata K., Yokoyama M. (2003). HMG-CoA reductase inhibitor has protective effects against stroke events in stroke-prone spontaneously hypertensive rats. Stroke.

[223] Kronenberg G., Balkaya M., Prinz V., Gertz K., Ji S., Kirste I., Heuser I., Kampmann B., Hellmann-Regen J., Gass P. (2012). Exofocal dopaminergic degeneration as antidepressant target in mouse model of poststroke depression. Biol. Psychiatry.

[224] Langhauser F., Kraft P., Gob E., Leinweber J., Schuhmann M.K., Lorenz K., Gelderblom M., Bittner S., Meuth S.G., Wiendl H. (2014). Blocking of α4 integrin does not protect from acute ischemic stroke in mice. Stroke.

[225] Lee J.H., Park S.Y., Shin Y.W., Kim C.D., Lee W.S., Hong K.W. (2007). Concurrent administration of cilostazol with donepezil effectively improves cognitive dysfunction with increased neuroprotection after chronic cerebral hypoperfusion in rats. Brain Res..

[226] Liesz A., Zhou W., Mracsko E., Karcher S., Bauer H., Schwarting S., Sun L., Bruder D., Stegemann S., Cerwenka A. (2011). Inhibition of lymphocyte trafficking shields the brain against deleterious neuroinflammation after stroke. Brain.

[227] Lin M., Sun W., Gong W., Ding Y., Zhuang Y., Hou Q. (2015). Ginsenoside Rg1 protects against transient focal cerebral ischemic injury and suppresses its systemic metabolic changes in cerabral injury rats. Acta Pharm. Sin. B.

[228] Liu L., Zhu L., Zou Y., Liu W., Zhang X., Wei X., Hu B., Chen J. (2014). Panax notoginseng saponins promotes stroke recovery by influencing expression of Nogo-A, NgR and p75NGF, in vitro and in vivo. Biol. Pharm. Bull..

[229] Liu X.Y., Zhou X.Y., Hou J.C., Zhu H., Wang Z., Liu J.X., Zheng Y.Q. (2015). Ginsenoside Rd promotes neurogenesis in rat brain after transient focal cerebral ischemia via activation of PI3K/Akt pathway. Acta Pharmacol. Sin..

[230] Llovera G., Hofmann K., Roth S., Salas-Perdomo A., Ferrer-Ferrer M., Perego C., Zanier E.R., Mamrak U., Rex A., Party H. (2015). Results of a preclinical randomized controlled multicenter trial (pRCT): Anti-CD49d treatment for acute brain ischemia. Sci. Transl. Med..

[231] Min D., Mao X., Wu K., Cao Y., Guo F., Zhu S., Xie N., Wang L., Chen T., Shaw C. (2012). Donepezil attenuates hippocampal neuronal damage and cognitive deficits after global cerebral ischemia in gerbils. Neurosci. Lett..

[232] Neumann J., Riek-Burchardt M., Herz J., Doeppner T.R., Konig R., Hutten H., Etemire E., Mann L., Klingberg A., Fischer T. (2015). Very-late-antigen-4 (VLA-4)-mediated brain invasion by neutrophils leads to interactions with microglia, increased ischemic injury and impaired behavior in experimental stroke. Acta Neuropathol..

[233] Onetti Y., Dantas A.P., Perez B., Cugota R., Chamorro A., Planas A.M., Vila E., Jimenez-Altayo F. (2015). Middle cerebral artery remodeling following transient brain ischemia is linked to early postischemic hyperemia: A target of uric acid treatment. Am. J. Physiol. Heart Circ. Physiol..

[234] Pradillo J.M., Denes A., Greenhalgh A.D., Boutin H., Drake C., McColl B.W., Barton E., Proctor S.D., Russell J.C., Rothwell N.J. (2012). Delayed administration of interleukin-1 receptor antagonist reduces ischemic brain damage and inflammation in comorbid rats. J. Cereb. Blood Flow Metab..

[235] Prinz V., Laufs U., Gertz K., Kronenberg G., Balkaya M., Leithner C., Lindauer U., Endres M. (2008). Intravenous rosuvastatin for acute stroke treatment: An animal study. Stroke.

[236] Relton J.K., Martin D., Thompson R.C., Russell D.A. (1996). Peripheral administration of interleukin-1 receptor antagonist inhibits brain damage after focal cerebral ischemia in the rat. Exp. Neurol..

[237] Relton J.K., Sloan K.E., Frew E.M., Whalley E.T., Adams S.P., Lobb R.R. (2001). Inhibition of α4 integrin protects against transient focal cerebral ischemia in normotensive and hypertensive rats. Stroke.

[238] Reuter B., Rodemer C., Grudzenski S., Meairs S., Bugert P., Hennerici M.G., Fatar M. (2015). Effect of simvastatin on mmps and timps in human brain endothelial cells and experimental stroke. Transl. Stroke Res..

[239] Rolland W.B., Lekic T., Krafft P.R., Hasegawa Y., Altay O., Hartman R., Ostrowski R., Manaenko A., Tang J., Zhang J.H. (2013). Fingolimod reduces cerebral lymphocyte infiltration in experimental models of rodent intracerebral hemorrhage. Exp. Neurol..

[240] Romanos E., Planas A.M., Amaro S., Chamorro A. (2007). Uric acid reduces brain damage and improves the benefits of rt-PA in a rat model of thromboembolic stroke. J. Cereb. Blood Flow Metab..

[241] Shi X., Yu W., Liu L., Liu W., Zhang X., Yang T., Chai L., Lou L., Gao Y., Zhu L. (2016). Panax notoginseng saponins administration modulates pro-/anti-inflammatory factor expression and improves neurologic outcome following permanent MCAO in rats. Metab. Brain Dis..

[242] Sironi L., Cimino M., Guerrini U., Calvio A.M., Lodetti B., Asdente M., Balduini W., Paoletti R., Tremoli E. (2003). Treatment with statins after induction of focal ischemia in rats reduces the extent of brain damage. Arterioscler. Thromb. Vasc. Biol..

[243] Uchino H., Elmer E., Uchino K., Li P.A., He Q.P., Smith M.L., Siesjo B.K. (1998). Amelioration by cyclosporin A of brain damage in transient forebrain ischemia in the rat. Brain Res..

[244] Wang T., Lv P., Jin W., Zhang H., Lang J., Fan M. (2014). Protective effect of donepezil hydrochloride on cerebral ischemia/reperfusion injury in mice. Mol. Med. Rep..

[245] Wu H.Y., Tang Y., Gao L.Y., Sun W.X., Hua Y., Yang S.B., Zhang Z.P., Liao G.Y., Zhou Q.G., Luo C.X. (2014). The synergetic effect of edaravone and borneol in the rat model of ischemic stroke. Eur. J. Pharmacol..

[246] Xie C.L., Li J.H., Wang W.W., Zheng G.Q., Wang L.X. (2015). Neuroprotective effect of ginsenoside-Rg1 on cerebral ischemia/reperfusion injury in rats by downregulating protease-activated receptor-1 expression. Life Sci..

[247] Yamasaki Y., Matsuura N., Shozuhara H., Onodera H., Itoyama Y., Kogure K. (1995). Interleukin-1 as a pathogenetic mediator of ischemic brain damage in rats. Stroke.

[248] Yamashita T., Sato T., Sakamoto K., Ishii H., Yamamoto J. (2015). The free-radical scavenger edaravone accelerates thrombolysis with alteplase in an experimental thrombosis model. Thromb. Res..

[249] Yang L.X., Zhang X., Zhao G. (2016). Ginsenoside Rd attenuates DNA damage by increasing expression of DNA glycosylase endonuclease VIII-like proteins after focal cerebral ischemia. Chin. Med. J..

[250] Yu G., Hess D.C., Borlongan C.V. (2004). Combined cyclosporine-a and methylprednisolone treatment exerts partial and transient neuroprotection against ischemic stroke. Brain Res..

[251] Yu Z.F., Bruce-Keller A.J., Goodman Y., Mattson M.P. (1998). Uric acid protects neurons against excitotoxic and metabolic insults in cell culture, and against focal ischemic brain injury in vivo. J. Neurosci. Res..

[252] Yuan H., Wang W.P., Feng N., Wang L., Wang X.L. (2011). Donepezil attenuated oxygen-glucose deprivation insult by blocking Kv2.1 potassium channels. Eur. J. Pharmacol..

[253] Yuen C.M., Sun C.K., Lin Y.C., Chang L.T., Kao Y.H., Yen C.H., Chen Y.L., Tsai T.H., Chua S., Shao P.L. (2011). Combination of cyclosporine and erythropoietin improves brain infarct size and neurological function in rats after ischemic stroke. J. Transl. Med..

[254] Sobowale O.A., Parry-Jones A.R., Smith C.J., Tyrrell P.J., Rothwell N.J., Allan S.M. (2016). Interleukin-1 in stroke: From bench to bedside. Stroke.

[255] Relton J.K., Rothwell N.J. (1992). Interleukin-1 receptor antagonist inhibits ischaemic and excitotoxic neuronal damage in the rat. Brain Res. Bull..

[256] Garcia J.H., Liu K.F., Relton J.K. (1995). Interleukin-1 receptor antagonist decreases the number of necrotic neurons in rats with middle cerebral artery occlusion. Am. J. Pathol..

[257] Loddick S.A., Rothwell N.J. (1996). Neuroprotective effects of human recombinant interleukin-1 receptor antagonist in focal cerebral ischaemia in the rat. J. Cereb. Blood Flow Metab..

[258] Boutin H., LeFeuvre R.A., Horai R., Asano M., Iwakura Y., Rothwell N.J. (2001). Role of IL-1α and IL-1β in ischemic brain damage. J. Neurosci..

[259] Banwell V., Sena E.S., Macleod M.R. (2009). Systematic review and stratified meta-analysis of the efficacy of interleukin-1 receptor antagonist in animal models of stroke. J. Stroke Cerebrovasc. Dis..

[260] McCann S.K., Cramond F., Macleod M.R., Sena E.S. (2016). Systematic review and meta-analysis of the efficacy of interleukin-1 receptor antagonist in animal models of stroke: An update. Transl. Stroke Res..

[261] Maysami S., Wong R., Pradillo J.M., Denes A., Dhungana H., Malm T., Koistinaho J., Orset C., Rahman M., Rubio M. (2016). A cross-laboratory preclinical study on the effectiveness of interleukin-1 receptor antagonist in stroke. J. Cereb. Blood Flow Metab..

[262] Singh N., Hopkins S.J., Hulme S., Galea J.P., Hoadley M., Vail A., Hutchinson P.J., Grainger S., Rothwell N.J., King A.T. (2014). The effect of intravenous interleukin-1 receptor antagonist on inflammatory mediators in cerebrospinal fluid after subarachnoid haemorrhage: A phase II randomised controlled trial. J. Neuroinflamm..

[263] Laufs U., Gertz K., Dirnagl U., Bohm M., Nickenig G., Endres M. (2002). Rosuvastatin, a new HMG-CoA reductase inhibitor, upregulates endothelial nitric oxide synthase and protects from ischemic stroke in mice. Brain Res..

[264] Baryan H.K., Allan S.M., Vail A., Smith C.J. (2012). Systematic review and meta-analysis of the efficacy of statins in experimental stroke. Int. J. Stroke.

[265] Krauth D., Anglemyer A., Philipps R., Bero L. (2014). Nonindustry-sponsored preclinical studies on statins yield greater efficacy estimates than industry-sponsored studies: A meta-analysis. PLoS Biol..

[266] Cohen J.A., Chun J. (2011). Mechanisms of fingolimod’s efficacy and adverse effects in multiple sclerosis. Ann. Neurol..

[267] Liu J., Zhang C., Tao W., Liu M. (2013). Systematic review and meta-analysis of the efficacy of sphingosine-1-phosphate (S1P) receptor agonist FTY720 (fingolimod) in animal models of stroke. Int. J. Neurosci..

[268] Smith C.J., Denes A., Tyrrell P.J., di Napoli M. (2015). Phase II anti-inflammatory and immune-modulating drugs for acute ischaemic stroke. Expert Opin. Investig. Drugs.

[269] Yoshiyama Y., Kojima A., Ishikawa C., Arai K. (2010). Anti-inflammatory action of donepezil ameliorates tau pathology, synaptic loss, and neurodegeneration in a tauopathy mouse model. J. Alzheimers Dis..

[270] Wang S.S., Wang Y.G., Chen H.Y., Wu Z.P., Xie H.G. (2013). Expression of genes encoding cytokines and corticotropin releasing factor are altered by citalopram in the hypothalamus of post-stroke depression rats. Neuroendocrinol. Lett..

[271] Dhami K.S., Churchward M.A., Baker G.B., Todd K.G. (2013). Fluoxetine and citalopram decrease microglial release of glutamate and d-serine to promote cortical neuronal viability following ischemic insult. Mol. Cell. Neurosci..

[272] Osman M.M., Lulic D., Glover L., Stahl C.E., Lau T., van Loveren H., Borlongan C.V. (2011). Cyclosporine-A as a neuroprotective agent against stroke: Its translation from laboratory research to clinical application. Neuropeptides.

[273] Chauhan A., Sharma U., Jagannathan N.R., Reeta K.H., Gupta Y.K. (2011). Rapamycin protects against middle cerebral artery occlusion induced focal cerebral ischemia in rats. Behav. Brain Res..

[274] Okamura K., Tsubokawa T., Johshita H., Miyazaki H., Shiokawa Y. (2014). Edaravone, a free radical scavenger, attenuates cerebral infarction and hemorrhagic infarction in rats with hyperglycemia. Neurol. Res..

[275] Sun Y.Y., Morozov Y.M., Yang D., Li Y., Dunn R.S., Rakic P., Chan P.H., Abe K., Lindquist D.M., Kuan C.Y. (2014). Synergy of combined tPA-edaravone therapy in experimental thrombotic stroke. PLoS ONE.

[276] Dohare P., Hyzinski-Garcia M.C., Vipani A., Bowens N.H., Nalwalk J.W., Feustel P.J., Keller R.W., Jourd’heuil D., Mongin A.A. (2014). The neuroprotective properties of the superoxide dismutase mimetic tempol correlate with its ability to reduce pathological glutamate release in a rodent model of stroke. Free Radic. Biol. Med..

[277] Scandinavian Simvastatin Survival Study Group (1994). Randomised trial of cholesterol lowering in 4444 patients with coronary heart disease: The Scandinavian simvastatin survival study (4S). Lancet.

[278] Amarenco P., Bogousslavsky J., Callahan A., Goldstein L.B., Hennerici M., Rudolph A.E., Sillesen H., Simunovic L., Szarek M., Welch K.M. (2006). High-dose atorvastatin after stroke or transient ischemic attack. N. Engl. J. Med..

[279] Amaro S., Obach V., Cervera A., Urra X., Gomez-Choco M., Planas A.M., Chamorro A. (2009). Course of matrix metalloproteinase-9 isoforms after the administration of uric acid in patients with acute stroke: A proof-of-concept study. J. Neurol..

[280] Barrett K.M., Brott T.G., Brown R.D., Carter R.E., Geske J.R., Graff-Radford N.R., McNeil R.B., Meschia J.F. (2011). Mayo Acute Stroke Trial for Enhancing Recovery Study Group. Enhancing recovery after acute ischemic stroke with donepezil as an adjuvant therapy to standard medical care: Results of a phase IIA clinical trial. J. Stroke Cerebrovasc. Dis..

[281] Chamorro A., Amaro S., Castellanos M., Segura T., Arenillas J., Marti-Fabregas J., Gallego J., Krupinski J., Gomis M., Canovas D. (2014). Safety and efficacy of uric acid in patients with acute stroke (URICO-ICTUS): A randomised, double-blind phase 2b/3 trial. Lancet Neurol..

[282] Edaravone Acute Infarction Study Group (2003). Effect of a novel free radical scavenger, edaravone (MCI-186), on acute brain infarction. Randomized, placebo-controlled, double-blind study at multicenters. Cerebrovasc. Dis..

[283] Emsley H.C., Smith C.J., Georgiou R.F., Vail A., Hopkins S.J., Rothwell N.J., Tyrrell P.J., Acute Stroke I. (2005). A randomised phase II study of interleukin-1 receptor antagonist in acute stroke patients. J. Neurol. Neurosurg. Psychiatry.

[284] Kaste M., Murayama S., Ford G.A., Dippel D.W., Walters M.R., Tatlisumak T. (2013). MCI-186 study group. Safety, tolerability and pharmacokinetics of MCI-186 in patients with acute ischemic stroke: New formulation and dosing regimen. Cerebrovasc. Dis..

[285] Liu X., Wang L., Wen A., Yang J., Yan Y., Song Y., Liu X., Ren H., Wu Y., Li Z. (2012). Ginsenoside-rd improves outcome of acute ischaemic stroke—A randomized, double-blind, placebo-controlled, multicenter trial. Eur. J. Neurol..

[286] Liu X., Xia J., Wang L., Song Y., Yang J., Yan Y., Ren H., Zhao G. (2009). Efficacy and safety of ginsenoside-Rd for acute ischaemic stroke: A randomized, double-blind, placebo-controlled, phase II multicenter trial. Eur. J. Neurol..

[287] Llull L., Laredo C., Renu A., Perez B., Vila E., Obach V., Urra X., Planas A., Amaro S., Chamorro A. (2015). Uric acid therapy improves clinical outcome in women with acute ischemic stroke. Stroke.

[288] Montaner J., Chacon P., Krupinski J., Rubio F., Millan M., Molina C.A., Hereu P., Quintana M., Alvarez-Sabin J. (2008). Simvastatin in the acute phase of ischemic stroke: A safety and efficacy pilot trial. Eur. J. Neurol..

[289] Nighoghossian N., Berthezene Y., Mechtouff L., Derex L., Cho T.H., Ritzenthaler T., Rheims S., Chauveau F., Bejot Y., Jacquin A. (2015). Cyclosporine in acute ischemic stroke. Neurology.

[290] Plehn J.F., Davis B.R., Sacks F.M., Rouleau J.L., Pfeffer M.A., Bernstein V., Cuddy T.E., Moye L.A., Piller L.B., Rutherford J. (1999). Reduction of stroke incidence after myocardial infarction with pravastatin: The Cholesterol and Recurrent Events (CARE) study. The care investigators. Circulation.

[291] Ridker P.M., Danielson E., Fonseca F.A., Genest J., Gotto A.M., Kastelein J.J., Koenig W., Libby P., Lorenzatti A.J., MacFadyen J.G. (2008). Rosuvastatin to prevent vascular events in men and women with elevated C-reactive protein. N. Engl. J. Med..

[292] Sever P.S., Dahlof B., Poulter N.R., Wedel H., Beevers G., Caulfield M., Collins R., Kjeldsen S.E., Kristinsson A., McInnes G.T. (2003). Prevention of coronary and stroke events with atorvastatin in hypertensive patients who have average or lower-than-average cholesterol concentrations, in the Anglo-Scandinavian Cardiac Outcomes Trial—Lipid Lowering Arm (ASCOT-LLA): A multicentre randomised controlled trial. Lancet.

[293] Shepherd J., Blauw G.J., Murphy M.B., Bollen E.L., Buckley B.M., Cobbe S.M., Ford I., Gaw A., Hyland M., Jukema J.W. (2002). Pravastatin in elderly individuals at risk of vascular disease (PROSPER): A randomised controlled trial. Lancet.

[294] Smith C.J., Emsley H.C., Udeh C.T., Vail A., Hoadley M.E., Rothwell N.J., Tyrrell P.J., Hopkins S.J. (2012). Interleukin-1 receptor antagonist reverses stroke-associated peripheral immune suppression. Cytokine.

[295] Takenaka K., Kato M., Yamauti K., Hayashi K. (2014). Simultaneous administration of recombinant tissue plasminogen activator and edaravone in acute cerebral ischemic stroke patients. J. Stroke Cerebrovasc. Dis..

[296] Ridker P.M., Thuren T., Zalewski A., Libby P. (2011). Interleukin-1β inhibition and the prevention of recurrent cardiovascular events: Rationale and design of the canakinumab anti-inflammatory thrombosis outcomes study (CANTOS). Am. Heart J..

[297] Montecucco F., Mach F. (2009). Update on statin-mediated anti-inflammatory activities in atherosclerosis. Semin. Immunopathol..

[298] Collins R., Armitage J., Parish S., Sleight P., Peto R. (2004). Heart Protection Study Collaborative Group. Effects of cholesterol-lowering with simvastatin on stroke and other major vascular events in 20,536 people with cerebrovascular disease or other high-risk conditions. Lancet.

[299] Montaner J., Bustamante A., Garcia-Matas S., Martinez-Zabaleta M., Jimenez C., de la Torre J., Rubio F.R., Segura T., Masjuan J., Canovas D. (2016). Combination of thrombolysis and statins in acute stroke is safe: Results of the STARS randomized trial (stroke treatment with acute reperfusion and simvastatin). Stroke.

[300] White H.D., Simes R.J., Anderson N.E., Hankey G.J., Watson J.D., Hunt D., Colquhoun D.M., Glasziou P., MacMahon S., Kirby A.C. (2000). Pravastatin therapy and the risk of stroke. N. Engl. J. Med..

[301] Waters D.D., Schwartz G.G., Olsson A.G., Zeiher A., Oliver M.F., Ganz P., Ezekowitz M., Chaitman B.R., Leslie S.J., Stern T. (2002). Effects of atorvastatin on stroke in patients with unstable angina or non-q-wave myocardial infarction: A myocardial ischemia reduction with aggressive cholesterol lowering (MIRACL) substudy. Circulation.

[302] Athyros V.G., Tziomalos K., Gossios T.D., Griva T., Anagnostis P., Kargiotis K., Pagourelias E.D., Theocharidou E., Karagiannis A., Mikhailidis D.P. (2010). Safety and efficacy of long-term statin treatment for cardiovascular events in patients with coronary heart disease and abnormal liver tests in the Greek Atorvastatin and Coronary Heart Disease Evaluation (GREACE) study: A post-hoc analysis. Lancet.

[303] Byington R.P., Jukema J.W., Salonen J.T., Pitt B., Bruschke A.V., Hoen H., Furberg C.D., Mancini G.B. (1995). Reduction in cardiovascular events during pravastatin therapy. Pooled analysis of clinical events of the pravastatin atherosclerosis intervention program. Circulation.

[304] Heo J.H., Song D., Nam H.S., Kim E.Y., Kim Y.D., Lee K.Y., Lee K.J., Yoo J., Kim Y.N., Lee B.C. (2016). Effect and safety of rosuvastatin in acute ischemic stroke. J. Stroke.

[305] Chung J.W., Hwang J., Lee M.J., Cha J., Bang O.Y. (2016). Previous statin use and high-resolution magnetic resonance imaging characteristics of intracranial atherosclerotic plaque: The intensive statin treatment in acute ischemic stroke patients with intracranial atherosclerosis study. Stroke.

[306] Moonis M., Kane K., Schwiderski U., Sandage B.W., Fisher M. (2005). HMG-CoA reductase inhibitors improve acute ischemic stroke outcome. Stroke.

[307] Stead L.G., Vaidyanathan L., Kumar G., Bellolio M.F., Brown R.D., Suravaram S., Enduri S., Gilmore R.M., Decker W.W. (2009). Statins in ischemic stroke: Just low-density lipoprotein lowering or more?. J. Stroke Cerebrovasc. Dis..

[308] Ni Chroinin D., Asplund K., Asberg S., Callaly E., Cuadrado-Godia E., Diez-Tejedor E., di Napoli M., Engelter S.T., Furie K.L., Giannopoulos S. (2013). Statin therapy and outcome after ischemic stroke: Systematic review and meta-analysis of observational studies and randomized trials. Stroke.

[309] Blanco M., Nombela F., Castellanos M., Rodriguez-Yanez M., Garcia-Gil M., Leira R., Lizasoain I., Serena J., Vivancos J., Moro M.A. (2007). Statin treatment withdrawal in ischemic stroke: A controlled randomized study. Neurology.

[310] Squizzato A., Romualdi E., Dentali F., Ageno W. (2011). Statins for acute ischemic stroke. Cochrane Database Syst. Rev..

[311] Hong K.S., Lee J.S. (2015). Statins in acute ischemic stroke: A systematic review. J. Stroke.

[312] Bonaventura A., Montecucco F., Dallegri F. (2016). Update on the effects of treatment with recombinant tissue-type plasminogen activator (rt-PA) in acute ischemic stroke. Expert Opin. Biol. Ther..

[313] Meseguer E., Mazighi M., Lapergue B., Labreuche J., Sirimarco G., Gonzalez-Valcarcel J., Lavallee P.C., Cabrejo L., Guidoux C., Klein I.F. (2012). Outcomes after thrombolysis in AIS according to prior statin use: A registry and review. Neurology.

[314] Martinez-Ramirez S., Delgado-Mederos R., Marin R., Suarez-Calvet M., Sainz M.P., Alejaldre A., Vidal-Jordana A., Marti-Vilalta J.L., Marti-Fabregas J. (2012). Statin pretreatment may increase the risk of symptomatic intracranial haemorrhage in thrombolysis for ischemic stroke: Results from a case-control study and a meta-analysis. J. Neurol..

[315] Ward M.D., Jones D.E., Goldman M.D. (2014). Overview and safety of fingolimod hydrochloride use in patients with multiple sclerosis. Expert Opin. Drug Saf..

[316] Yoon S.Y., Kim J.K., An Y.S., Kim Y.W. (2015). Effect of donepezil on wernicke aphasia after bilateral middle cerebral artery infarction: Subtraction analysis of brain F-18 fluorodeoxyglucose positron emission tomographic images. Clin. Neuropharmacol..

[317] Chang W.H., Park Y.H., Ohn S.H., Park C.H., Lee P.K., Kim Y.H. (2011). Neural correlates of donepezil-induced cognitive improvement in patients with right hemisphere stroke: A pilot study. Neuropsychol. Rehabil..

[318] Kraglund K.L., Mortensen J.K., Grove E.L., Johnsen S.P., Andersen G. (2015). Talos: A multicenter, randomized, double-blind, placebo-controlled trial to test the effects of citalopram in patients with acute stroke. Int. J. Stroke.

[319] Simats A., Garcia-Berrocoso T., Montaner J. (2016). Natalizumab: A new therapy for acute ischemic stroke?. Expert Rev. Neurother..

[320] Kern R., Nagayama M., Toyoda K., Steiner T., Hennerici M.G., Shinohara Y. (2013). Comparison of the European and Japanese guidelines for the management of ischemic stroke. Cerebrovasc. Dis..

[321] Shinohara Y., Saito I., Kobayashi S., Uchiyama S. (2009). Edaravone (radical scavenger) versus sodium ozagrel (antiplatelet agent) in acute noncardioembolic ischemic stroke (EDO trial). Cerebrovasc. Dis..

[322] Isahaya K., Yamada K., Yamatoku M., Sakurai K., Takaishi S., Kato B., Hirayama T., Hasegawa Y. (2012). Effects of edaravone, a free radical scavenger, on serum levels of inflammatory biomarkers in acute brain infarction. J. Stroke Cerebrovasc. Dis..

[323] Tsuruoka A., Atsumi C., Mizukami H., Imai T., Hagiwara Y., Hasegawa Y. (2014). Effects of edaravone, a free radical scavenger, on circulating levels of MMP-9 and hemorrhagic transformation in patients with intravenous thrombolysis using low-dose alteplase. J. Stroke Cerebrovasc. Dis..

[324] Zheng J., Chen X. (2016). Edaravone offers neuroprotection for acute diabetic stroke patients. Ir. J. Med. Sci..

[325] Satani N., Savitz S.I. (2016). Is immunomodulation a principal mechanism underlying how cell-based therapies enhance stroke recovery?. Neurotherapeutics.

[326] Azodi S., Jacobson S. (2016). Cytokine therapies in neurological disease. Neurotherapeutics.

